# Digital logic circuits in yeast with CRISPR-dCas9 NOR gates

**DOI:** 10.1038/ncomms15459

**Published:** 2017-05-25

**Authors:** Miles W. Gander, Justin D. Vrana, William E. Voje, James M. Carothers, Eric Klavins

**Affiliations:** 1 Department of Electrical Engineering, University of Washington, Seattle, Washington 98195, USA; 2 Department of Bioengineering, University of Washington, Seattle, Washington 98195, USA; 3 Department of Chemical Engineering, University of Washington, Seattle, Washington 98195, USA; 4 Center for Synthetic Biology, University of Washington, Seattle, Washington 98195, USA

## Abstract

Natural genetic circuits enable cells to make sophisticated digital decisions. Building equally complex synthetic circuits in eukaryotes remains difficult, however, because commonly used components leak transcriptionally, do not arbitrarily interconnect or do not have digital responses. Here, we designed *dCas9-Mxi1-*based NOR gates in *Saccharomyces cerevisiae* that allow arbitrary connectivity and large genetic circuits. Because we used the chromatin remodeller *Mxi1*, our gates showed minimal leak and digital responses. We built a combinatorial library of NOR gates that directly convert guide RNA (gRNA) inputs into gRNA outputs, enabling the gates to be ‘wired' together. We constructed logic circuits with up to seven gRNAs, including repression cascades with up to seven layers. Modelling predicted the NOR gates have effectively zero transcriptional leak explaining the limited signal degradation in the circuits. Our approach enabled the largest, eukaryotic gene circuits to date and will form the basis for large, synthetic, cellular decision-making systems.

## Abstract (web summary)

The leakiness of commonly used genetic components can make the construction of complex synthetic circuits difficult. Here the authors construct NOR gate architecture, using dCas9 fused to the chromatin remodeller Mxi1, that can be wired together into complex circuits.

Living cells make decisions based on information processing genetic programmes. Many of these programmes execute digital functions^1^,^2^,^3^,^4^,^5^,^6^,^7^,^8^. The capability to build synthetic digital systems in living cells could allow engineers to build novel decision-making regulatory networks for use in a variety of applications^9^, ranging from gene therapies that modify cell state based on sensed information^10^,^11^ to entirely new developmental programmes for tissue engineering^12^,^13^. In electronics a compositional approach has allowed the construction of digital circuits of great complexity to be quickly designed and implemented. Here, we have developed set of low-variability genetic parts that can be routinely composed to create large digital circuits in yeast cells.

Genetic components that implement simple logical operations, which in principle could be interconnected to form complex logic functions, have been demonstrated^14^,^15^,^16^,^17^,^18^,^19^,^20^,^21^,^22^,^23^. DNA-binding domains (DBDs) such as zinc fingers and TALEs (transcription activator-like effectors) have been used to construct libraries of transcription factors in eukaryotes^19^,^24^,^25^,^26^,^27^. However, scaling with DBDs in eukaryotes has been difficult because of challenges in synthesizing libraries of orthogonal parts^28^,^29^. Libraries of DBD-based parts have been shown in prokaryotes, but extensive part characterization and computer-aided design (CAD) was necessary to identify part combinations that yielded functional logic circuits^22^. Recently, programmable and orthogonal CRISPR-dCas9 transcription factors have been employed^18^,^20^,^30^,^31^,^32^,^33^,^34^ to build up to five component circuits using *dCas9*-mediated repression in prokaryotes^18^. Transcriptional repression in these circuits is likely due to steric hindrance of RNA polymerase by *dCas9*. Although *dCas9* allows for programmable interconnections, its response function is leaky leading to signal degradation when layered^18^. Site-specific recombinases have been employed in genetic circuits as a means to reduce leak^35^,^36^,^37^, but there are a limited number of such enzymes restricting the scalability of this approach. Here, we address these issues, advancing the art of engineering living digital circuits by focusing on two main engineering goals.

First, we built a universal, single-gene NOR logic gate; the NOR gates are functionally complete^38^ and as such can be composed to implement any logic function. Crucially, the input and output signals of our gates have the same molecular types while still being programmable so that, as in electronics, gates can be wired together. To achieve this, we made use of the CRISPR-dCas9 system: the signals in our framework are guide RNAs (gRNAs) whose sequences specifically match up to programmable target sequences on our NOR gate promoters.

Second, we required a consistent ‘OFF' state for our NOR gates. To achieve this, we used the chromatin remodelling repression domain *Mxi1* to take advantage of the eukaryotic cell's ability to repress gene expression, by fusing this domain to *dCas9* (ref. 30). The *Mxi1* domain is thought to recruit histone deacetylases^39^,^40^, and with it we observed strong transcriptional repression in our circuits. The strong and consistent ‘OFF' behaviour we observe with our NOR gates is a key factor that allows them to be composed into larger circuits by minimizing accumulation of transcriptional leak with every added layer. A mathematical model of our NOR gates predicts that they have effectively no transcriptional leak in their OFF states. We show that with low leak there exist parameters that allow our NOR gates to be composed without significant signal degradation. More importantly, we show experimentally that we can build a variety of digital logic circuits composed of up to five NOR gates and seven internal gRNA wires, as well as cascades of gates with up to seven layers that still have digital responses according to our specifications.

In summary, we developed low-variability single-gene NOR gates that can be regularly interconnected into arbitrary topologies that implement large digital circuits in yeast cells. Neither meticulous characterization of individual parts nor sophisticated design tools were necessary to find combinations of NOR gates that conferred functional circuits. Because the technology is essentially generic and easy to rewire, it can in principle be used to implement arbitrary internal logic for a variety of synthetic cellular decision-making systems, such as those being explored for diagnostics^11^,^41^, therapeutics^41^,^42^ and development^43^,^44^.

## Results

### NOR gate architecture

**Figure 1:**
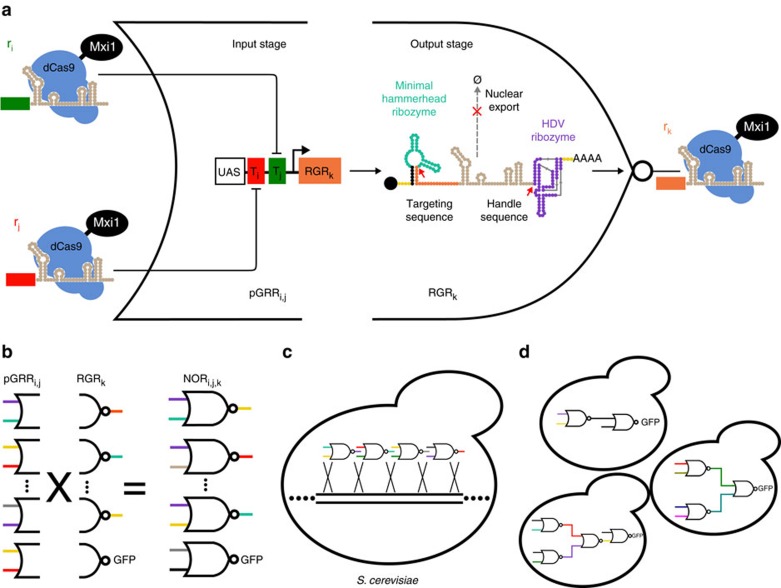
Schematic of the NOR gate architecture and circuit composition. (**a**) A NOR gate input stage consists of a Pol II pGRR promoter that is fully repressed by the binding of either one or both of its cognate gRNA-dCas9-Mxi1 complexes. The output stage of the NOR gate is a gRNA transcript, flanked by self-cleaving ribozymes (RGR). Cleavage sites are indicated by red arrows. The cleavage of the ribozymes prevents nuclear export of the gRNA, indicated by dotted grey arrow. (**b**) The process of NOR gate library construction. Our library consists of a set of 400 two-input pGRR promoters and 20 RGR outputs for a total of 8,000 possible NOR gates. (**c**) Genomically integrating NOR gates into *S. cerevisiae*. (**d**) Arbitrary circuits are constructed by integrating multiple NOR gates into a single strain.

We built a universal, single-gene logic gate, in our case a NOR gate (Fig. 1a). The NOR gate outputs are then gRNAs that match the target sequences on other NOR gate promoters (Fig. 1b). Our NOR gates are genomically integrated into yeast cells (Fig. 1c). We avoided using RNA polymerase (Pol) III promoters to express gRNAs^20^,^30^,^31^,^34^ because they have low expression levels relative to Pol II promoters and are more difficult to engineer^45^,^46^. By programming the NOR gate input target sequences and output gRNA sequences in a set of gates, we were able to construct a variety of circuit topologies (Fig. 1d).

**Figure 2:**
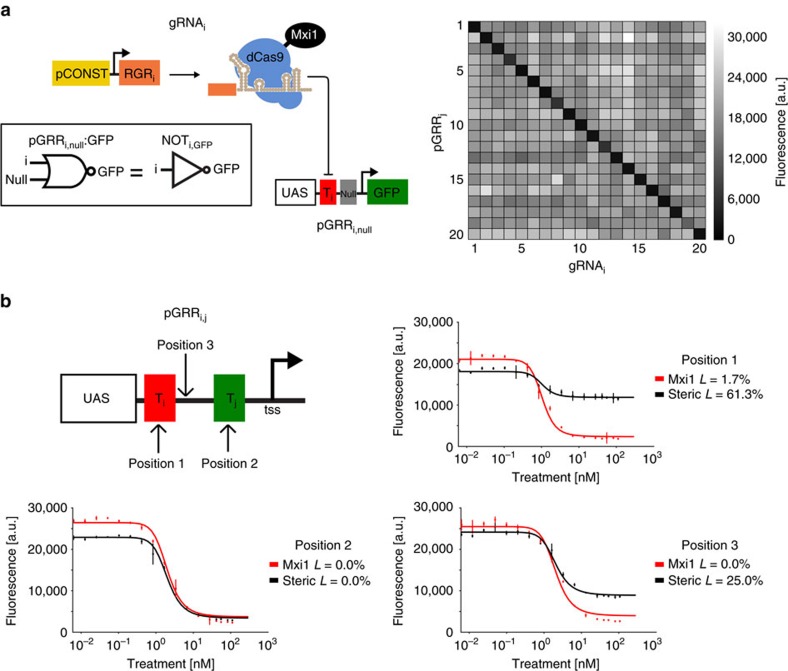
Orthogonality and repression via *dCas9-Mxi1*. (**a**) A constitutive promoter drives expression of gRNAs paired with a combinatorial library of cognate promoters. Orthogonality of the gRNA guide sequences was tested by crossing the 20 pGRR_i,null_ promoters, each expressing *GFP*, with the 20 gRNA_i_, creating 400 different strains of yeast. Fluorescence values of each strain were measured using flow cytometry. Fluorescence values from one biological replicate are displayed in the matrix. (**b**) Dose response curves are shown for repression via *dCas9-Mxi1* and *dCas9* repression via steric hindrance of pGRR driving GFP at three separate positions in the promoter. The three positions are annotated on the pGRR promoter representation. At all three positions, at maximal induction, *dCas9-Mxi1* represses the promoter to a lower fluorescence level than *dCas9* alone. Model fits predicted the parameter value *L*, representing transcriptional leak, for all curves. At all three positions the predicted *L* value is as small or smaller for *dCas9-Mix1* than for steric repression. Error bars represent the s.d. of three biological replicates measured over three separate experiments.

Second, we required a consistent ‘OFF' state for our NOR gates that corresponded to complete or near complete repression of the output promoter (Supplementary Fig. 1). we used the chromatin remodelling repression domain *Mxi1* to take advantage of the eukaryotic cell's ability to repress gene expression, by fusing this domain to *dCas9* (ref. 30) (Fig. 2a). When compared with a number of repression domains, *Mxi1* showed the strongest repression (Supplementary Fig. 2). Our results suggest that such repression provides a significantly improved and more consistent ‘OFF' signal compared with repression via steric hindrance (Fig. 2b), in which *dCas9* is interfering with transcriptional initiation, but is not remodelling chromatin. A mathematical model of our NOR gates, fit to both steady-state and time response data, predicts them have effectively zero transcriptional leak in their OFF states. Additionally, the model predicts that repression via steric hindrance leaks more than repression via *dCas9-Mxi1* (Fig. 2b).

Our approach allowed for the construction of the largest eukaryotic gene circuits, to the best of our knowledge, ever demonstrated (Table 1).

The gate NOR_i,j,k_, with input signals r_i_ and r_j_ and output r_k_, consists of a gRNA-responsive Pol II promoter (pGRR_i,j_) input stage, driving an output stage, ribozyme-flanked gRNA (RGR_k_) (Fig. 1a). According to NOR logic, r_k_ is high only when both r_i_ and r_j_ are low. A signal, r_i_, is defined as a gRNA complexed with a *dCas9-Mix1* fusion protein that confers strong transcriptional repression when bound to DNA^30^. The gRNA signals are distinguished by their unique 5′ guide sequence. A 20-component library of signals defining r_1_–r_20_ was used in this work (Supplementary Table 1). The pGRR_i,j_ promoter contains two, 20 base-pair (bp) target sites that match r_i_ and r_j_ respectively. Since we designed 20 signals, there are 20^3^=8,000 total NOR gates in the set. A NOR_i,j,k_ functions as a NOT_j,k_ if the pGRR_i,j_ contains two identical target sites, if the pGRR_i,j_ contains only one target site from the 20 component library (pGRR_i,null_) or if r_i_ is simply not used in the circuit. A target sequence of ‘null' refers to a pGRR that contains a target sequence that does not match any gRNA used in the containing circuit.

### Input stage promoter design

The pGRR_i,j_ promoter is tightly repressed when gRNA-*dCas9-Mxi1* is bound to one or both of its two 20 bp target sites. The core region of the pGRR_i,j_, the minimal pCYC1 promoter, was chosen based on its successful use with *dCas9* in the past^32^. Because the promoter has relatively low expression levels and we wanted its output to have a strong ON output when not repressed, an upstream activating sequence (UAS) from the strong pGPD promoter^47^ was added, forming the base pGRR promoter. The UAS increased the unrepressed expression level of the pGRR output by approximately threefold while maintaining the same OFF state expression level in the presence of r_i_ and r_j_, further separating the digital ON and digital OFF levels (Supplementary Fig. 3a). A pGRR promoter map highlighting all relevant sequence features is included in Supplementary Fig. 4. A library of 11 pGRR_i,j_ promoters, with i and j chosen from the 20 guide sequences, showed limited expression variability when driving *GFP*, with an ∼18% s.d. from the mean (Supplementary Fig. 3b) Of the 20 pGRR_i,null_:*GFP* constructs (i ranging from 1 to 20), 16 were repressed to or near the level of *Saccharomyces cerevisiae* autofluorescence in the presence of the corresponding signal r_i_ (Supplementary Fig. 1).

### Output stage RNA design

Two different RNA pol II expression methods were used in this work (Supplementary Fig. 5). The first was an RGR design utilizing a 5′ minimal hammerhead ribozyme (mHH) and a 3′ hepatitis delta virus ribozyme (HDV), flanking the gRNA^48^. The second was an ‘insulated' RGR (iRGR) with the mHH replaced by an avocado sunblotch viroid (ASBV) ribozyme. Both designs are intended to post-transcriptionally remove nuclear export signals, the 5′ cap and 3′ poly-A tail^49^,^50^. It has been shown that RNA device folding can be insulated from surrounding sequence context through computational sequence selection^51^,^52^. Ten guide sequences were chosen for the RGR architecture that were computationally predicted to confer proper folding of the mHH 5′ ribozyme. Ten more guide sequences were chosen for the iRGR context whose ASBV 5′ ribozyme is predicted to fold properly regardless of guide sequence. We observed similar levels of *dCas9-Mxi1-*mediated repression with gRNAs expressed from both iRGR and RGR constructs (Supplementary Fig. 6). Interestingly, RGR transcripts lacking a 5′ ribozyme also showed *dCas9-Mxi1-*mediated repression. These results are consistent with previous studies that indicate a majority of 5′ extended gRNA target sequences are processed to 20 nucleotides^53^. No significant crosstalk was observed when all r_1–10_ (RGR design) and r_11–20_ (iRGR design) were paired with all pGRR_1-20,null_:*GFP* among noncognate pairs (Fig. 2a and Supplementary Fig. 7). Out of 20 total RGRs (RGR_1–10_ and iRGR_11–20_) when targeted to their cognate pGRR_1-20,null_:*GFP* constructs, 16 repressed fluorescence to or near the level of autofluorescence for *S. cerevisiae* (Supplementary Fig. 1).

### Logic circuits

**Figure 3:**
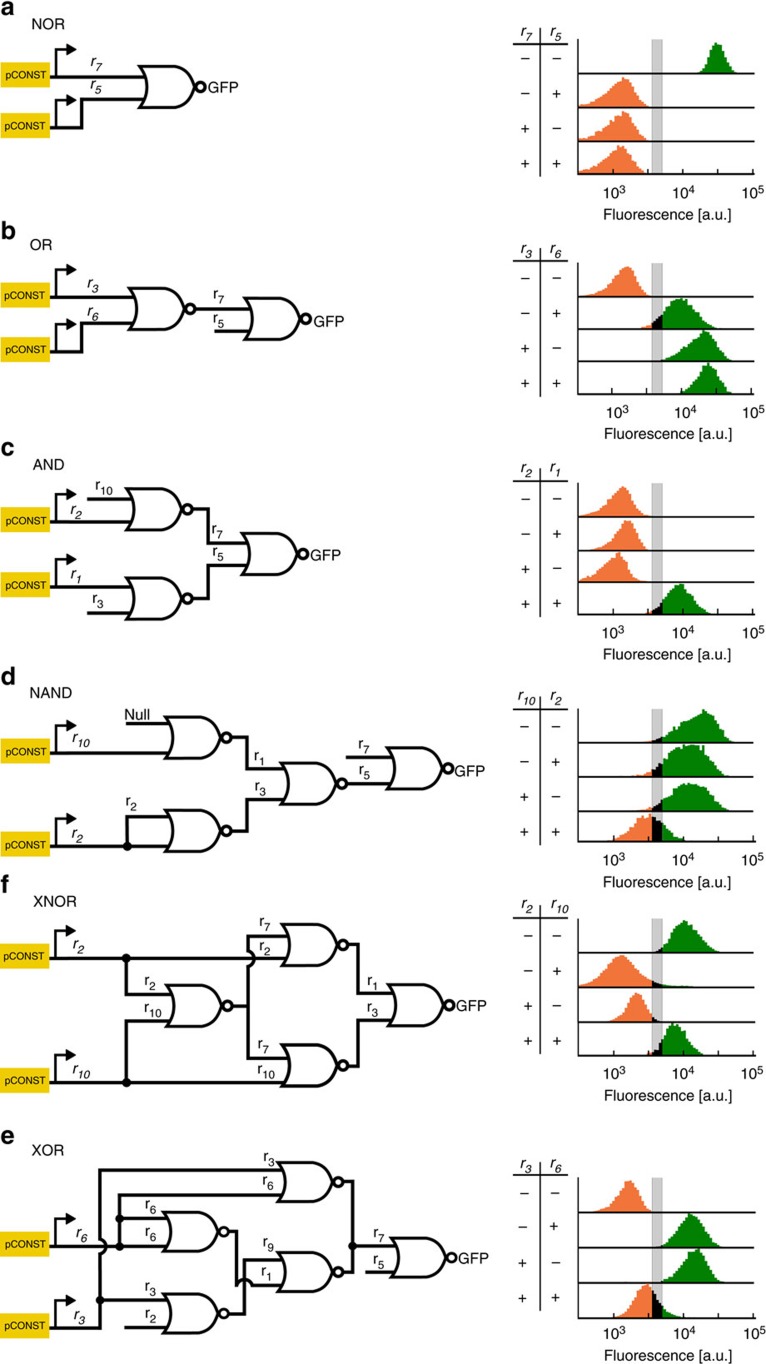
NOR gate-based logic circuits. (**a**–**f**) Six different two-input logic circuits constructed by interconnecting NOR gates. For each of the four input possibilities (−−, −+, +−, and ++), a distinct strain was constructed with the corresponding inputs expressed off of constitutive promoters (for logical +), or not integrated at all (for logical −). Fluorescence values were collected using flow cytometry of cells growing in log phase. The histograms represent population fraction from three different biological replicates measured during a single experiment and were normalized so that area sums to unity. Fluorescence population ratios of the circuits are included in the Supplementary Table 3.

As a demonstration of the complex circuits possible with our NOR gates, six two-input, one-output digital logic circuits were built by integrating up to five NOR gate cassettes into various selectable loci in the yeast genome (Fig. 3a–f). The output of each circuit was made observable by having the last NOR gate drive the expression of *GFP*. The circuits were constructed from the 16 guide sequences of the 20-component library that exhibited the strongest repression (Supplementary Fig. 1). The truth table for each gate was experimentally obtained by constructing four separate strains, one for each pair of possible input values, in which the corresponding gRNA input signals were expressed from constitutive promoters (Supplementary Table 2).

We observed fluorescence intensity differences in the digital ON and OFF states in various circuits. To distinguish circuit state, value bands for digital ON, OFF and Undefined, fluorescence values were determined with the 16 guide sequences and their cognate pGRR promoters used in circuit construction (Supplementary Fig. 8). For the state of a circuit to be considered ON or OFF we specified that a majority of cell population fall in the expected fluorescence band. Population fraction tables for all circuits can be found in Supplementary Table 3.

Circuits containing different NOR gate variants can exhibit a range of behaviours. For example, 15 versions of the XOR, from Fig. 3e, constructed using different NOR gates exhibited a range of performance (Supplementary Fig. 9). We hypothesize that circuit performance variations are due to expression differences in the pGRR promoters and repression efficiency variations of the gRNA in the individual NOR gates of the circuit.

### Cascades

**Figure 4:**
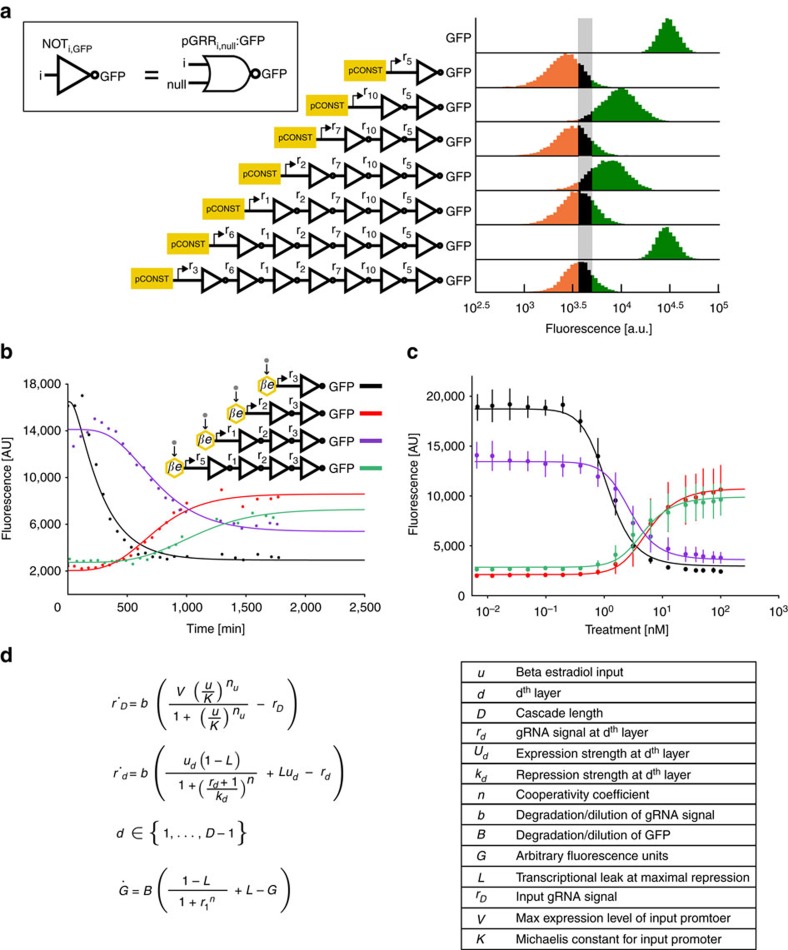
Repression cascade characterization. (**a**) Repression cascades of one to seven gRNAs. Cascades were created with sequential genomic integrations of NOT gates. The final output of each cascade is a NOT gate that expresses *GFP*. Each NOT gate represses the output of a subsequent NOT gate. Cascades with an even number of layers express a high level of *GFP*, creating a digital ON output, and odd depth cascades express low levels of *GFP*, creating a digital OFF output. Fluorescence measurements were taken using flow cytometry. The histograms represent population fraction from three different biological replicates measured during a single experiment and were normalized so that area sums to unity. Fluorescence population ratios of the circuits are included in Supplementary Table 3. (**b**) Temporal dynamics for cascades of one to four gRNAs. Expression of the input gRNA was induced with β-estradiol. A model of the cascade, in which each layer is treated as a Hill function, was used to fit the data. The plot shows the data from one biological replicate. As the number of layers in the cascade increases, signal degradation and increased time to steady state is observed. (**c**) The steady-state response function for the four inducible cascades. Error bars represent the s.d. of three biological replicates measured over three separate experiments. (**d**) A representation of the model. The model was used to generate the fits for the steady-state and kinetic inducible cascade experiments.

To test the limits of size and complexity our NOR gate circuits can achieve inverter cascades of depth one through seven were composed with NOT gates (Fig. 4a). The cascade of depth *D* was made by the addition of a NOT gate to repress the input stage of the depth *D*–1 cascade. Each successive addition of a NOT gate inverter resulted in switching the behaviour of the output *GFP* expression. As seen previously with the two-input logic circuits, there is considerable variability within the ON and OFF states. However, circuits that are expected to exhibit ON or OFF behaviour are clearly distinguishable from one another according to our digital ON and OFF specification. As cascade depth increased the fluorescence levels of the OFF states for all of the odd depth cascades increased. Similarly, except for the cascade of depth 6, as cascade depth increased the fluorescence levels of the ON states decreased. This suggests a gradual degradation of circuit function as the number of layers increased. Similar behaviour was also observed for other repression cascades that were constructed (Supplementary Fig. 10). Alternative versions of 6 gRNA cascades were constructed and showed variability in their levels of ON (Supplementary Fig. 11).

To investigate the temporal characteristics of the inverter cascades, we analysed the kinetics of cascades of depth one through four. A *β*-estradiol-inducible promoter^54^ was used to activate transcription of the input gRNA and *GFP* expression was periodically measured over the course of ∼30 h of log phase growth (Fig. 4b). With increasing cascade depth, a clear delay in output response was evident, with the cascades reaching half-maximal expression at 4.1±0.5, 10.8±1.0, 12.0±1.2 and 17.8±1.0 h (residual s.d. deviation) for cascades of depth one through four respectively. The dose response curves of the four cascades were also measured after passaging cells over 5 days (Fig. 4c). Consistent with the steady-state cascades, the induction of a gRNA targeting the input of the cascade switched the output of the cascade from OFF to ON (even depth cascades) or from ON to OFF (odd depth cascades). Some signal degradation with successive layers was observed (Fig. 4c), suggesting a limit to the possible depth of the cascades.

### Mathematical modelling

A kinetic model was constructed to capture the behaviour of our synthetic cascades. The model combines successive Hill functions to represent simple transcription and repression associated with each gRNA-*dCas9-Mxi1* signal. The parameters *v*_*d*_ and *k*_*d*_ roughly capture expression and repression strengths of the promoters driving each gRNA-*dCas9-Mxi1* signal, *r*_*d*_. The parameter *L* represents the transcriptional leak as a percentage of the maximal expression of a given gate when maximally repressed parameters *n* and *b* capture the cooperativity of repression. Degradation/dilution of gRNA-*dCas9-Mxi1* signals respectively (Fig. 4d). The steady-state dose response and kinetic time course for inducible cascade data were both fit to the model (Fig. 4b,c). Due to the different growth conditions of the steady-state and kinetic cascade experiments, two separate model fits were generated for each experiment. As inducible cascades were built in such a way that they shared many of the same pGRR and gRNA components (Fig. 4b), parameters for the one-, two-, three- and four-layer cascades were shared between the models and fit simultaneously. To address potential model identifiability issues parameter values were constrained based on published biological values (Supplementary Table 4). The fitting results were found to correlate well with the experimental data. The measured ∼18% s.d. from the mean for the promoter strength values matches well with the ∼24% s.d. from the mean of the promoter strength parameters, *v*_*d*_ (Supplementary Table 4).

Model fits of the steady-state and time course data predict the transcriptional leak of repression due to *dcas9-Mxi1*, the value of *L*, to be effectively zero, *L=*0.6±0.1% (s.d.), equivalent to the production of roughly one transcript every 5 to 10 cell divisions. The reported value of *L* was calculated as the average of the predicted transcriptional leak from the model fits from Fig. 2b. To demonstrate the ability of *dCas9-Mxi1* to decrease transcriptional leak compared with steric repression via *dCas9*, gRNA dose response curves of repression at three pGRR promoter target site positions were performed using *dCas9* and *dCas9-Mxi1* (Fig. 2b). At maximal induction, *dCas9-Mxi1* represses the promoter to a lower fluorescence level than *dCas9* alone at all three positions. Repression via steric hindrance showed promoter positional variations in predicted leak parameter values. The observed positional variation is consistent with previous results^32^. In all three positions *dCas9-Mxi1* was predicted to have the same or lower leak parameter *L*. These data indicate that in the context of our NOR gates, *dCas9-Mxi1* confers stronger and more consistent repression than *dCas9* alone. Alternative plots comparing *dCas9* and *dCas9-Mxi1* repression as a function of inducible promoter activation driving gRNA are included in Supplementary Fig. 12.

**Figure 5:**
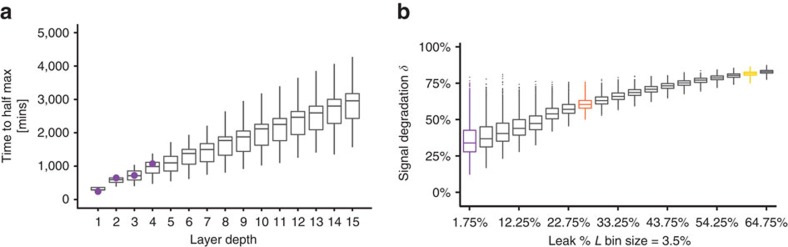
Model predictions and analysis of repression cascades. (**a**) Simulations of time to half-maximal response using the model. Increasingly layered cascades show a positive linear relationship between circuit time to half-maximal response and circuit depth, with a slope of 184.9±0.2 (s.e.m.) min layer^−1^. The first four data points highlighted in purple are experimental data from Fig. 4b. (**b**) Signal degradation, *δ*, in a cascade increases as transcriptional leak of the gates increase. Boxplots of *δ* values were plotted with binned values of the leak parameter *L*. At values of *L* <1.75% the spread of performance of the cascades is significantly larger. The bin containing the steady-state experimentally predicted value of *dCas9-Mxi1*, *L=*0.6±0.1% (s.d.), is highlighted in purple. The bins highlighted in orange and yellow contain the predicted *L* values for the steric repression measurements in Fig. 2 of position 1, *L*=25.0%, and position 3, *L*=61.3%, respectively.

The temporal responses of the cascades were predicted from simulations using randomly sampled parameters within the range of the model fit. Parameter values for kinetic simulations were resampled from the model fit using the kinetic time course experimental data. Response times were found to rise linearly (*r*^2^=0.83) with increasing circuit depth. Linear regression analysis estimated the slope of the increase in response time per layer to be equal to 184.9±0.2 (s.e.m.) min layer^−1^ (Fig. 5a), consistent with our experimental results. Response delay was found to depend primarily on the degradation/dilution rate *b* of gRNA-*dCas9-Mxi1* (Supplementary Fig. 13) that controls the overall timescale of the dynamics.

To extrapolate the model to predict the effect of leak on signal degradation for deeper cascades, cascades of various lengths were simulated, with increasing values of *L*, using randomly sampled parameter sets within the range of dose response experimental fits. Dynamic range of a cascade length *D*, *ρ*_*D*_, was calculated for each cascade. Here dynamic range is defined as the log fold change of the maximal and minimal response of a cascade, 
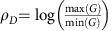
. A log-linear relationship was found between *ρ*_*D*_ and *D*. This relationship was used to calculate the signal degradation, *δ*, representing the percent loss in dynamic range per each additional layer (Fig. 5b).

Signal degradation was found to be largely dependent on the transcriptional leak parameter, *L* (Fig. 5b and Supplementary Fig. 12). As leak increases, *δ*, on average, increases. At values of *L* >80%, the median value of *δ* trends to ∼80%. At values of *L* <1.75%, the spread of performance of the cascades is significantly larger. In this range the performance of the cascade is more sensitive to other parameters in the model. Our estimate of leak from the dose response experiments, *L=* 0.6±0.1% (s.d.), falls within the sensitive range, indicating the importance of utilizing well-performing NOR gates in large circuits built using our architecture. In addition, these data show the significance of reducing NOR gate leak when constructing larger circuits.

## Discussion

We introduced a class of *dCas9*-based modular genetic NOR gates that behave digitally, have low variability and show minimal retroactivity or effects on cell growth. These features made these gates relatively easy to combine into Boolean logic circuits that are among the largest ever built in any organism. In particular, we found that most circuits in Figs 3 and 4 required that only a handful of gate combinations be screened to identify a functional design, and others required only one.

Table 1 compares our technology with selected published circuits. We measured circuit complexity with a combination of two metrics: the number of gates and the number of connections among gates, allowing us to locate circuits in a two-dimensional plot (Supplementary Fig. 14). We can calculate a complexity score using the two metrics, complexity=(gates^2^+connections^2^)^1/2^. For example, the XOR gate had five gates and four connections, producing a complexity of (5^2^+4^2^)^1/2^=6.4, while the cascade has a complexity of (7^2^+1^2^)^1/2^=9.2. These complexities compare well with gene circuits developed in *Escherichia coli*, for example. Our NOR gates enabled extremely simple design and construction of large gene circuits. Before genetic circuits can be made much larger, however, many factors that influence the size and complexity of synthetic genetic circuits must be addressed.

First, the gates in any framework must be well behaved. Gates can suffer from retroactivity, where a downstream gate affects the behaviour of upstream gates to which it is not connected by design^55^,^56^,^57^. In this case it is quite difficult to design large circuits even with CAD because we may not know the source of the retroactivity, how to model it or how to design with it. In addition, gates can be highly variable, where the outputs levels of one gate do not match the input levels of the next. Electrical engineers call this an impedance mismatch. A recent paper^22^ addressed retroactivity by adding insulators to their gates. By meticulously characterizing the performance each gate, and using CAD, they were able to select compatible subsets of parts out of which they constructed circuits as large as those demonstrated here, despite gate variability. Not all of the circuits predicted to work by the CAD tool functioned correctly, possibly due to residual retroactive effects, requiring the circuits to be screened for function. In contrast, our gates are considerably less variable and do not seem to be confounded by retroactive effects, at least in circuits with complexities <9.0. In such a case and when circuit sizes are small (<20 components) circuits are easy to design by hand since any subset of components from a library is likely to yield a functional circuit. Thus, in our case, the design problem is easy enough that extensive part characterization and CAD tools were not necessary at the circuit level (even though CAD tools such as standard DNA editors and secondary structure predictors for RNA were used at the sequence level).

Second, the host organism presents many unique challenges. Each organism can be thought of as a different computer operating system. Promoters, for example, in *E. coli* are ∼60 bp of DNA long, and transcriptional regulation is a fairly well-understood process^58^. In contrast, the size of promoter and regulatory regions vary widely and can range from 250 bp to 10 kb in yeast and other eukaryotes. Transcriptional regulation in eukaryotes is complex, involving a variety of mechanisms including chromatin remodelling^59^,^60^,^61^,^62^,^63^, and understanding it remains a highly active area of research^64^. Therefore, unfortunately, any genetic circuit technology designed for one kingdom of life is unlikely to be easily ‘ported' to another, especially those built on transcriptional or translational processes. Thus, directly comparing circuit architectures between organisms, as we did between yeast and *E. co*li in Table 1, is difficult. Nevertheless, we believe that because CRISPR-dCas9 functions in mammalian cells^20^,^30^,^31^,^32^,^34^,^48^, and the human *Mxi1* repression domain has been used in synthetic contexts to regulate transcription in human cells^30^,^39^,^40^, our NOR gates could be ported into mammalian cells, with difficulties of strain engineering likely dominating.

Third, the method by which circuits are constructed and the genetic tractability of the host affects progress toward building large circuits. For example, the circuits we present here are all singly integrated into the yeast genome, because plasmid-based systems exhibit cell-to-cell variation in copy number. That made the process of building and testing strains slow, costly and cumbersome and in fact limited our ability to build circuits much larger than those shown here. Larger circuits and large libraries of circuit variants will require that we develop, for example, one-pot assembly methods for large DNA constructs^65^. Depending on the technology, such assemblies may be more or less difficult to harness. For example, our circuits currently benefit from the fact that the gates are integrated into disparate genetic locations that decreases the possibility of interference between gates due to chromatin remodelling^62^,^66^ and of yeast's tendency to recombine nearby homologous regions^67^.

The success or failure of different approaches to building bigger circuits may depend on how well behaved, insulated, simple and scalable the input low-level devices and gates are. In addition, relaxing the requirement that circuits be digital, so that analogue or mixed analogue/digital circuits can be used when appropriate, will likely open up the design space, further increasing the size of the circuits we can build so that one day they can match the size and performance of natural genetic circuits.

## Methods

### Construction of yeast strains

Yeast transformations were carried out using a standard lithium acetate protocol^68^. Yeast cells were made competent by growing 50 ml cultures in rich media to log growth phase, then spinning down the cells and washing with H_2_0. Next, linearized DNA, salmon sperm donor DNA, 50% polyethylene glycol and 1 M LiOAc were combined with 50 μl of competent cells and the mixture was heat shocked at 42 °C for 15 min. The cells were then spun down, supernatant was removed and they were resuspended in H_2_O and then plated on selective agar media. Transformations were done into MATa W303-1A and MATalpha W303-1B background strains. Matings of the MATa and MATalpha were performed by coculturing both mating types and plating the culture onto selective agar media. All strains and sequences used in this work are detailed in the Supplementary Data 1.

### RNA design

RGR and iRGR sequences were computationally designed to enable the 5′ hammerhead ribozymes to fold into their target, functionally active, structures. ViennaRNA (RNAfold 2.1.9) was used to simulate long timescale (thermodynamic equilibrium) at an input temperature of 37 °C. Kinefold (kinefold_long_static_bianary 20060404) was used to simulate short timescale folding (cotranscriptional folding) with inputs of low and high polymerization rates of 25 and 50 nt s^−1^ respectively, helix minimum free energy=6.346 kcal mol^−1^ and folded without pseudoknots or entanglements. A total of 12 Kinefold simulations were run for each candidate sequence and agglomerated to generate average folding trace data.

Ribozyme target structures needed for both viennaRNA and Kinefold simulation evaluation were determined by folding ribozyme sequences (Minimal HH: 5′- NNNNNNCTGATGAGTCCGTGAGGACGAAACGAGTAAGCTCGTCNNNNNN-3′ ASBV1: 5′-GGGACGGGCCATCATCTATCCCTGAAGAGAC GAAGGCTTCGGCCAAGTCGAAACGGAAACGTCGGATAGTCGCCCGTCCC-3′) using RNAfold and Kinefold (melt and anneal of 1 min), respectively. RGR targeting sequences and iRGR insulating sequences were screened in specific 5′ promoter contexts (pGAL1min: 5′-AGTATCAACAAAAAATTGTTAATATACCTCTATACT TTAACGTCAAGGAGAAAAAACTATACGGATTCTAGAACTAGTGGATCTACAAA-3′, pAHD1: 5′-CAAGCTATACCAAGCATACAATCAACTATCTCATATACAGGATTCTAGAA CTAGTGGATCTACAAA-3′, pCYC1: 5′-ACTATACTTCTATAGACACACAAACACAAATACACACACTAATCTAGATATTGGATTCT AGAACTAGTGGATCTACAAA-3′) and in the 3′ context of the targeting sequence and the gRNA handle sequence (gRNA handle: 5′-GTTTTAGAGCTAGAAATAGCAAGTTAAAATAAGGCTAGTCCGTTATCAACTTGAAAAAG TGGCACCGAGTCGGTGCTTTT-3′).

Randomly generated 20 bp candidate targeting sequences for RGR, of which the most 5′ 6 bp defined the closing stem of the minimal HH ribozyme, were folded in the context of each promoter to confirm that the target structure was present in the MFE structure (viennaRNA) and that the target structure was present at >90% in the RNA folding trace at both low and high polymerase rates (Kinefold). Targeting sequences that enabled correct folding in the context of each promoter were considered successful. For iRGRs, randomly generated 5′ and 3′ insulating sequences were designed for each of the three promoter types and were screened for function in the same manner. However, to select for the most robust insulating sequences, each was screened against 75 randomly generated and 10 randomly generated 20 bp guide sequences using viennaRNA and Kinefold, respectively.

### Cytometry

Fluorescence intensity was measured with a BD Accuri C6 flow cytometer equipped with a CSampler plate adapter using excitation wavelengths of 488 and 640 nm and an emission detection filter at 533 nm (FL1 channel). A total of 10,000 events above a 400,000 FSC-H threshold (to exclude debris) were recorded for each sample with and core size of 22 mm using the Accuri C6 CFlow Sampler software. Cytometry data were exported as FCS 3.0 files and processed using the flowCore R software package and custom R scripts (Supplementary Software 1) to obtain the mean FL1-A value at each data point.

### Data collection for orthogonality matrix

Cytometry readings were taken with cultures inoculated into synthetic complete with cells from freshly struck out on agar. Colonies were picked from plates and grown for 3 h at 30 °C before reads were taken.

### Data collection for logic circuits and static cascades

Cytometry measurements were taken on cells grown in cultures diluted 1:1,000 from saturated culture for 16 h at 30 °C.

### Data collection for inducible cascades

Cells from saturated culture were diluted 1:100 into fresh media with a Beta Estradiol (*βe*) concentration of 100 nm. Cytometry measurements were taken over an ∼30 h period. During the time course, cells were periodically diluted to keep them in log growth phase. Experimental data collected for steady state were measured for four strains, each containing four different *βe*-inducible cascades. Each of the four strains was induced with 18 different doses of *βe* ranging from 0 to 100 μM in a single batch of 72 cultures. Cells were diluted every 8–15 h to prevent culture saturation. Steady-state fluorescence readings were taken after 5 days when the cultures were in log phase.

### Model description

A deterministic model of our system was described by three ordinary differential equations characterizing transcription, degradation and repression. The gRNA-*dCas9-Mxi1* and green fluorescent protein (GFP) molecular constituents were modelled as follows:


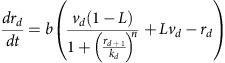



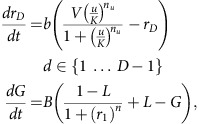


*r*_*d*_ is the concentration of the *d*_th_ gRNA-*dCas9-Mxi1*, *d* ranges from 1 to *D*−1, where *D* is the number of layers in the cascade; *r*_*D*_ is the input gRNA driven by the inducible promoter; *v*_*d*_ is the promoter strength driving each *r*_*d*_ in terms of the maximum steady-state concentration of gRNA from the promoter; *G* is the measurable normalized concentration of GFP; *b* is the degradation/dilution rate of all *r*_*d*_; *B* is the degradation/dilution for GFP; *k*_***d***_ is the repression strength of *r*_*d*_ to its cognate promoter, in terms of the number of repressors required to suppress a promoter to half strength; to its cognate promoter is modelled with *k*_*d*_, the number of repressors required to suppress a promoter to half-strength; and *n* is a Hill coefficient. For the transfer function, *V*, *K*, *n*_*u*_ respectively represent the maximum transcription, Michaelis–Menten constant and Hill coefficient of the inducible promoter; *u* is the input *βe* in μM. Concentration is rescaled as the Michaelis–Menten constant or the number of gRNAs required to suppress a NOT gate to half-maximal. Note that the model makes the assumptions that (1) there is no crosstalk between gRNA components, (2) *Mxi1* represses transcription completely with no transcriptional leak and (3) *dCas9-Mxi1* bind quickly and irreversibly to gRNA.

### Fitting procedure

Parameters were optimized using differential evolution followed by minimization using the BFGS (Broyden–Fletcher–Goldfarb–Shanno) algorithm^69^. For the steady-state experiments, optimal parameter fits for the parameters *v*_0_^ss^*−v*_3_^ss^, *k*_0_^ss^*−k*_3_^ss^, *V*^ss^, *n*^ss^ were generated from three separate experiments. For each of the three experiments, 17 parameter fits were generated using differential evolution/BFGS and means were calculated for a total of 51 steady-state parameter sets. The means from each experiment were used to determine the experimental error (*σ*) for estimating each parameter (Supplementary Table 4). For the kinetics experiments, five parameter fits for *v*_0_^kinetics^*−v*_3_^kinetics^, *k*_0_^kinetics^*−k*_3_^kinetics^, *b*, *B*, *V*^kinetic*s*^, *n*^kinetics^ were generated from a single experiment (Supplementary Table 4). As there were only data for a single kinetics experiment, experimental errors for the kinetic parameter values were not calculated. Parameters *K* and *n*_*u*_ were determined in a separate experiment by driving a YFP with the pGALZ4 β-estradiol inducible; this promoter is the same promoter used in the inducible cascades. The kinetics and steady-state parameter sets were resampled in downstream analyses to generate Monte Carlo simulations of longer repression cascades (Supplementary Software 1).

### Model predictions

Long repression cascades of 1 to 11 (*D* ∈ {1 … 11}) layers were simulated using the system of ordinary differential equations. Parameters for simulated cascades were generated by resampling parameter sets generated during the fitting procedure. For the kinetic model predictions, 10,000 simulated cascades were generated by resampling parameters from 5 parameter sets estimated from the kinetics experiment. The time-to-half max of *GFP* (*G*) was calculated for each cascade length *D* and plotted in Fig. 5a. For the signal degradation (*δ*) predictions in Fig. 5b, 100,000 simulated cascades of length *D*=7 were simulated by resampling parameters from the 51 parameter sets estimated from the 3 steady-state experiments. To compare *L* versus *δ*, *L* was sampled from a uniform distribution between 0 and 1. Signal degradation (*δ*) was calculated as the percent change in dynamic range per additional layer. The dynamic range at each layer *d* in a cascade of length *D* was calculated as:


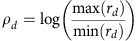


Dynamic range was found to have a log-linear relationship with the length of the cascade, and hence the average slope between *d* versus log(*ρ*_*d*_) was calculated using linear regression for each of the 100,000 simulations of cascades of length D by:


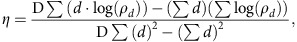


with *D*=7. With *η* being the change in log(*ρ*_*d*_) with each additional layer, the percent loss in dynamic range per layer or signal degradation *δ* is calculated as


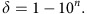


Values for *L* were binned using a bin size of 0.035 and *δ* versus *L* was plotted to generate Fig. 5b.

### Data availability

No data sets were generated during the current study. All data values supporting the experimental conclusions are shown either in main or Supplementary Figures (source data and DNA are available from corresponding author). A list of strains and sequences used for plasmids constructed are included in Supplementary Data 1. Custom software used in this work is available in Supplementary Software 1.

## Additional information

**How to cite this article:** Gander, M. W. *et al*. Digital logic circuits in yeast with CRISPR-dCas9 NOR gates. *Nat. Commun.* **8,** 15459 doi: 10.1038/ncomms15459 (2017).

**Publisher's note**: Springer Nature remains neutral with regard to jurisdictional claims in published maps and institutional affiliations.

## Supplementary Material

Supplementary Information Supplementary Figures, Supplementary Tables and Supplementary References

Supplementary Data 1 Strain and sequences list

Supplementary Software 1 R cytometry data processing scripts and mathematical modeling scripts

Peer Review File

## Figures and Tables

**Table 1:** Synthetic circuit size comparison.

**Publication**	**No. of gates/parts**	**No. of connections**	**No. of inputs**	**Circuit complexity (gates**^**2**^**+connections**^**2**^**)**^**1/2**^	**Functionally complete parts?**	**Medium**
Cascade circuit	7	6	1	9.22	Yes	*S. cerevisiae*
Nielsen *et al*.^22^	7	6	3	9.22	Yes	*E. coli*
Qian *et al*.^70^	6	5	4	7.81	Yes	*In vitro*
XOR circuit	5	4	2	6.40	Yes	*S. cerevisiae*
Xie *et al*.^11^	5	4	6	6.40	No	Mammalian
Auslander *et al*.^71^	5	4	2	6.40	No	Mammalian
Regot *et al*.^72^	5	3	2	5.83	Yes	Multicellular *S. cerevisiae*
Nissim *et al*.^33^	5	3	1	5.83	No	Mammalian
Stanton *et al*.^19^	4	3	2	5	Yes	*E. coli*
Nielsen *et al*.^18^	3	2	2	3.61	Yes	*E. coli*
Kiani *et al*.^20^	2	2	1	2.83	No	Mammalian

The best method for quantifying the size of synthetic biological circuits is an open question. Here we took the largest synthetic circuits constructed in recent publications and compared them with the two largest circuits from this paper. We separated the inputs to the circuits from internal components. We also counted the number of connections between the internal components. By our definition, a ‘part' is a molecular species that carries information necessary for the internal function of the circuit (as opposed to a helper protein such as cas9). A ‘connection' is a molecular interaction between parts that propagates information within the circuit.
