# Supplementary material for: Digital logic circuits in yeast with CRISPR-dCas9 NOR gates
Source: Nat Commun. 2017 May 25;8:15459. doi: 10.1038/ncomms15459 (PMC5458518; doi:10.1038/ncomms15459)
Supplement: Supplementary Information — Supplementary Figures, Supplementary Tables and Supplementary References [file ncomms15459-s1.pdf]

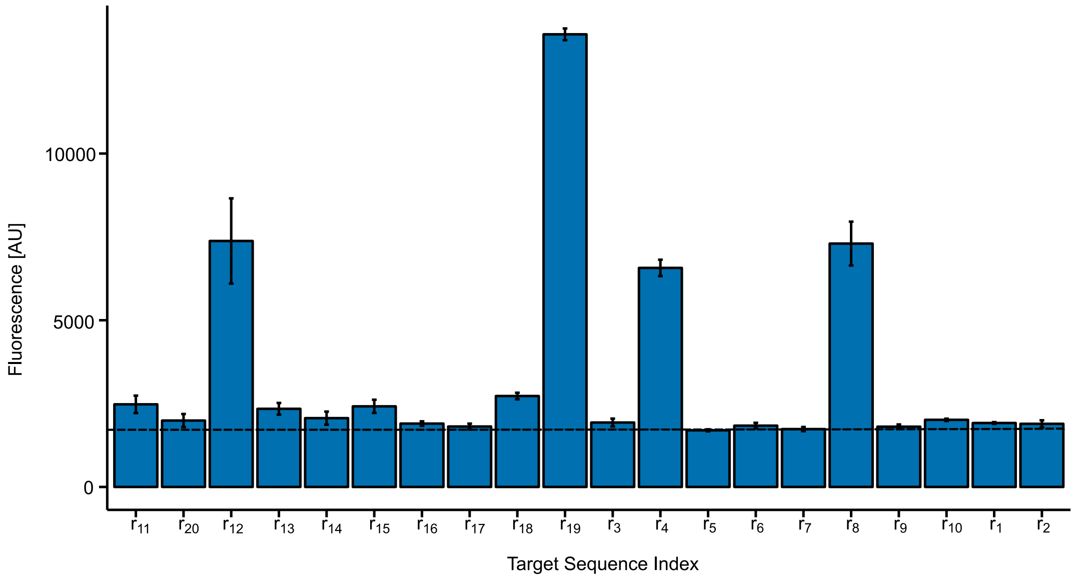

Supplementary Figure 1 | Diagonal of orthogonality matrix repression variation Bar chart representation of the diagonal of the orthogonality matrix from figure 2. Sixteen of the twenty guide sequences, when matched with their cognate promoter, show *GFP* repression near or at the level of autofluorescence for diploid *S. cerevisiae.* Autofluorescence, 1718.63 AU, is indicated by the black dashed line. Four of the guide sequences exhibit significantly worse repression. The sixteen sequences that exhibit strong repression also exhibit variation in level of repression, indicating different levels of efficacy for each guide sequence. Error bars are standard deviation of fluorescence measurements from three biological replicates collected during one experimental run.

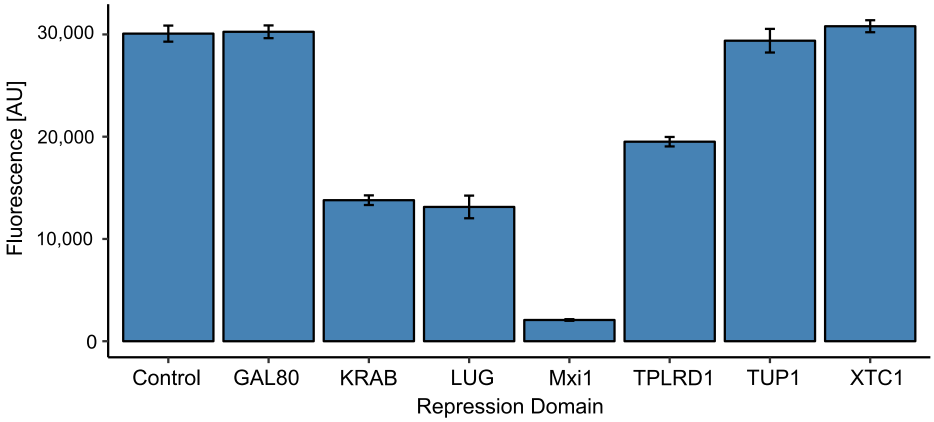

Supplementary Figure 2 | Repression Domain Comparison Protein fusions of *dCas9* and a panel of repression domains that have been shown to function in yeast were compared for repression level. Repression domain fusions were expressed constitutively, along with a pGRR promoter driving GFP and a constitutively expressed cognate RGR. The control strain contains a pGRR promoter driving GFP alone. The repression domains tested are *GAL80*[1], *KRAB*[2], *LUG*[3], *Mxi1*[4], *TPLRD1*[5], *TUP1*[6] and *XTC1*[7]. *Mxi1* shows the greatest amount of repression. Error bars are standard deviation of fluorescence measurements from three biological replicates collected during one experimental run.

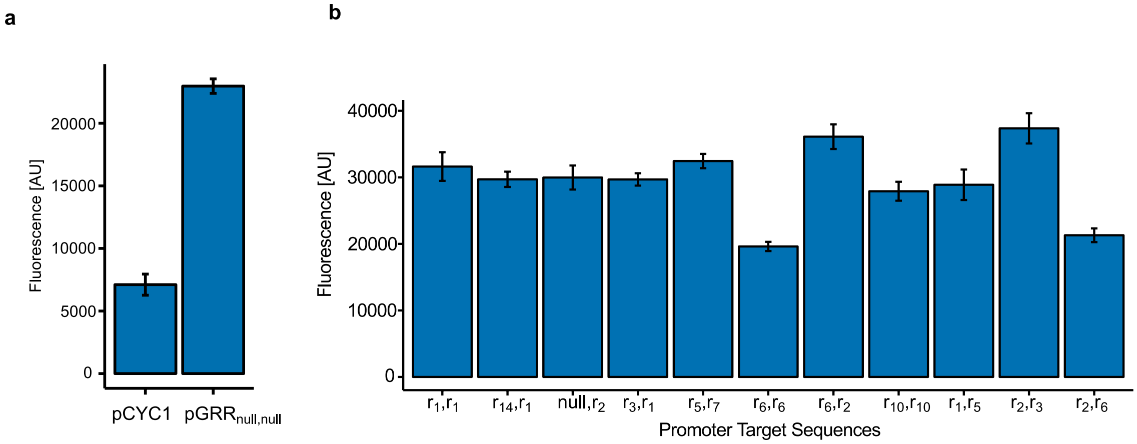

Supplementary Figure 3 | pGRR promoter variability a Addition of the pGPD upstream activating sequence (UAS), to the pCYC1 minimal promoter, increases the expression of *GFP* 3.23 fold. Error bars represent the standard deviation of three biological replicates collected during one experimental run. **b** A subset of 11 $pGRR_{i,j}$ promoters driving *GFP* has a mean fluorescence of 29511.78 [AU], a standard deviation of 5357.249 [AU] and a range of 17751.67 [AU]. Error bars represent the standard deviation of three biological replicates collected during one experimental run.

a

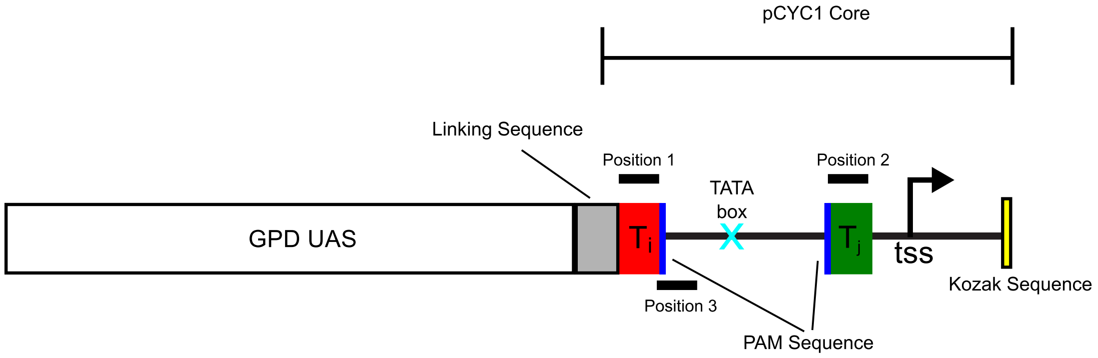

b

agtttatcattatcaatactcgccatttcaaagaatacgtaaataattaatagtagtgattttcctaactttatttagtcaaaaaattagccttttaa
ttctgctgtaacccgtacatgcccaaaatagggggcgggttacacagaatatataacatcgtaggtgtctgggtgaacagtttattcctggc
atccactaaatataatggagcccgctttttaagctggcatccagaaaaaaaaagaatcccagcaccaaaatattgttttcttcaccaacca
tcagttcataggtccattctcttagcgcaactacagagaacaggggcacaaacaggcaaaaaacgggcacaacctcaatggagtgat
gcaacctgcctggagtaaatgatgacacaaggcaattgacccacgcatgtatctatctcattttcttacaccttctattaccttctgctctctctg
atttggaaaaagctgaaaaaaaaggttgaaaccagttccctgaaattattcccctacttgactaataagtaaattcctgcagcccgggtac
tgtatNNNNNNNNNNNNNNNNNNNNtggcatgcatgtgctctgtatgtatataaaactcttgttttcttcttttctctaaatattctttc
ctNNNNNNNNNNNNNNNNNNNNgcataaatt[redacted]ctatacttctatagacacacaaacacaaatacacacactaatctagat
attggattctagaactagtggatctacaaa

Supplementary Figure 4 | pGRR promoter sequence structure a A sequence structure diagram that includes annotations of relevant sequence elements of the $pGRR_{i,j}$ promoter is shown. The positions 1, 2 and 3 targeted in figure 2b are marked on the diagram. **b** The sequence of a typical pGRR promoter is shown along with highlighted portions that correspond to annotations from extend data figure 3a. Colors of the annotations match the diagram in Supplementary figure 3a. The red and green highlighted text corresponds to target sites i and j respectively are listed as 20 Ns to indicate the programmability of these sequence elements. The non-highlighted sequence upstream of the grey highlighted portion of sequence is the GPD UAS.

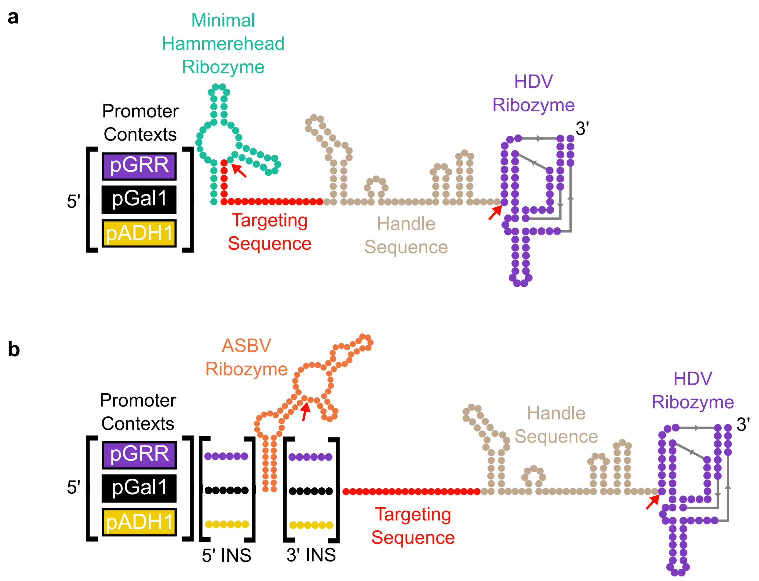

Supplementary Figure 5 | Schematic of pol II gRNA expression systems a The RGR architecture. All RGR constructs have guide sequences that were computationally predicted to confer proper folding of the minimal hammerhead ribozyme in all three promoter sequence contexts used in the work. Cleavage sites are indicated by red arrows. **b** The insulated RGR (iRGR) architecture. The iRGR has unique 5' and 3' insulating sequences, designed for three promoter sequence contexts, flanking the ASBV ribozyme. In the presence of the insulating sequences, proper ASBV folding is predicted for the majority of guide sequences. Cleavage sites are indicated by red arrows.

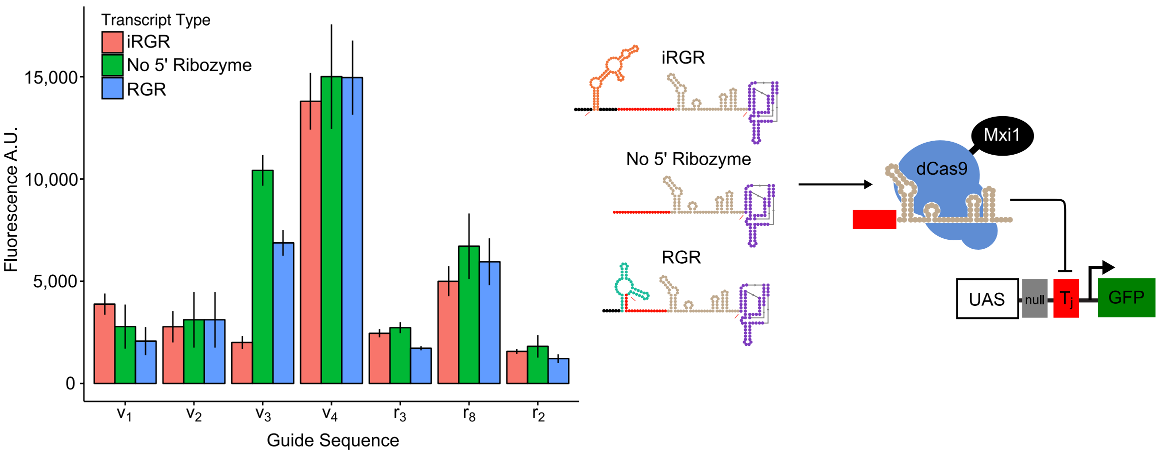

Supplementary Figure 6 | Comparison of pol II gRNA expression designs Seven gRNAs were expressed via three different designs, the RGR, the iRGR and an altered RGR design lacking the 5’ ribozyme. Guide sequences r_2, r_3, and r_8 were drawn from the gRNAs used in the main body of this paper, while guide sequences v_1-v_4 were randomly generated guide sequences not contained within the original 20 component library. Fluorescence levels of repressed cognate pGRR promoters were measured via flow cytometry and error bars indicate standard deviation from 6 biological replicates, except for r_3 RGR, r_2 iRGR and r_8 iRGR which represent 5 biological replicates. Data was collected across two different experimental runs. For all three transcript types, across all seven guide sequences except for v_3, we observed comparable gRNA mediated repression of pGRR promoters. These data suggest that for many of guide sequences, the 5’ ribozyme is not a contributing factor in the behavior of the gRNAs in our system.

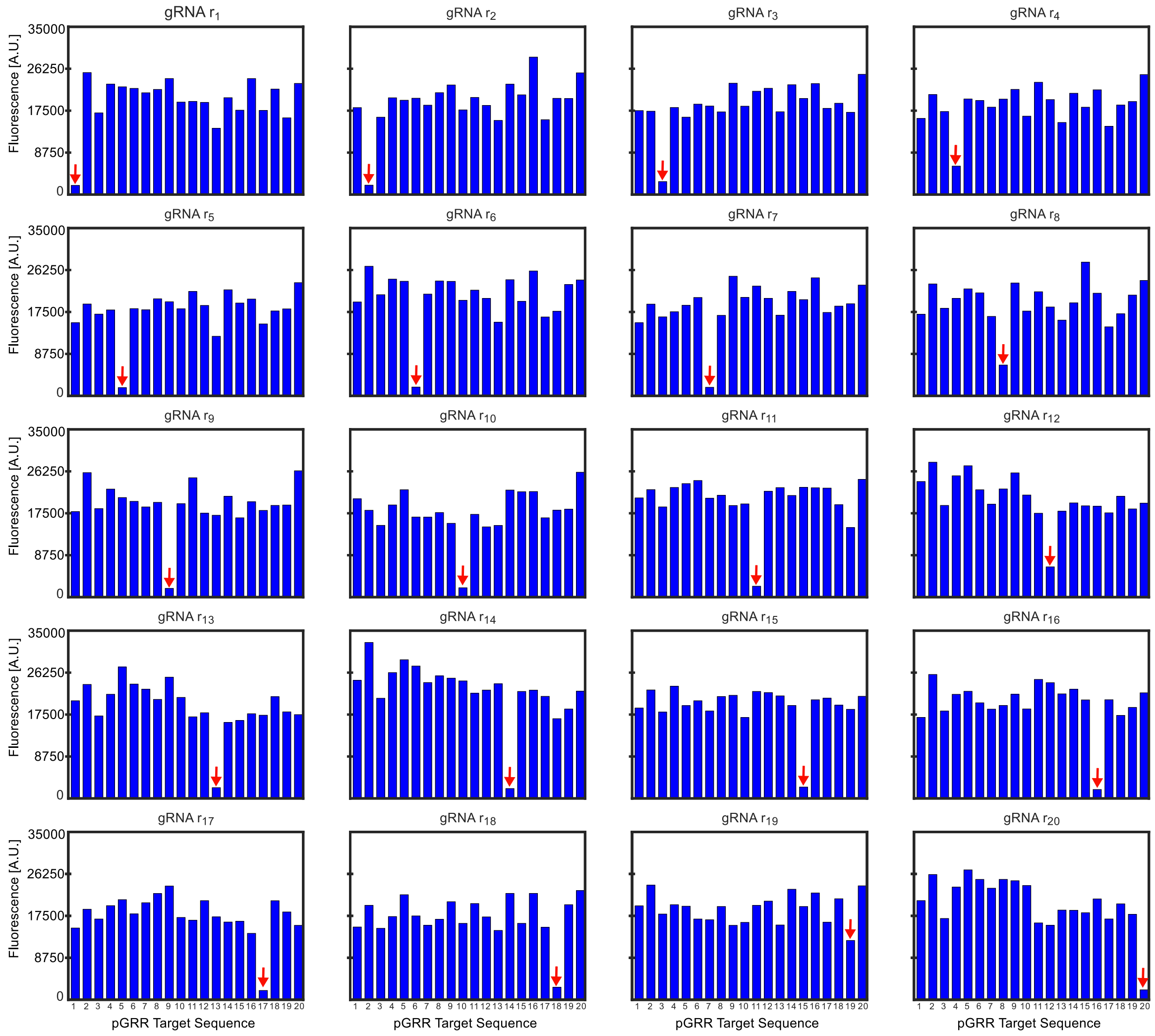

Supplementary Figure 7 | Bar chart of fluorescence values of orthogonality matrix Fluorescence values for all 400 strains in the orthogonality matrix. The strains are segmented by the 20 gRNA target sequences. Promoter target sequence index are in the same order for each subplot. Red arrows indicate a cognate pair of gRNA and pGRR promoter.

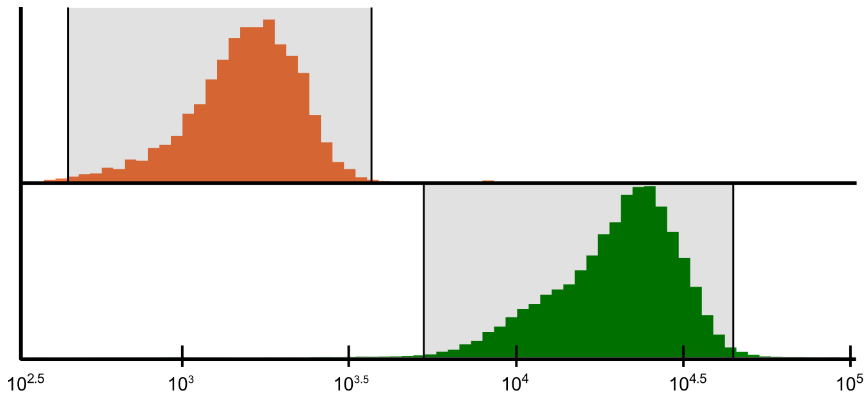

Supplementary Figure 8 | ON OFF and Undefined fluorescence intervals The ON and OFF histograms used to specify the fluorescence intervals for the circuits are shown with their middle 99% mass ranges. The histograms were generated by measuring fluorescence of a set of strains with pGRR promoters driving GFP with and without their cognate gRNAs. The OFF interval was defined by the 16 strains of the diagonal of the orthogonality matrix that were used in circuit construction (Fig. 2 and Supplementary Fig. 1). The ON interval was defined by the 16 unrepressed cognate promoters of the gRNAs used in the circuit construction. The histograms represent the sum of three biological replicates of the two strain sets. The intervals were defined by the middle 99% range of the histograms. The OFF set histogram has an upper middle 99% mass value of 3650 [AU]. The ON set histogram has a lower middle 99% mass value of 5039 [AU]. We specified the undefined interval range from 3650 [AU] to 5039 [AU]. OFF interval ranges from 0 [AU] to 3650 [AU]. The ON interval ranges from 5039 [AU] to ∞ [AU]. For a circuit to be considered in the ON or OFF state a majority of cell population must be in the correct interval.

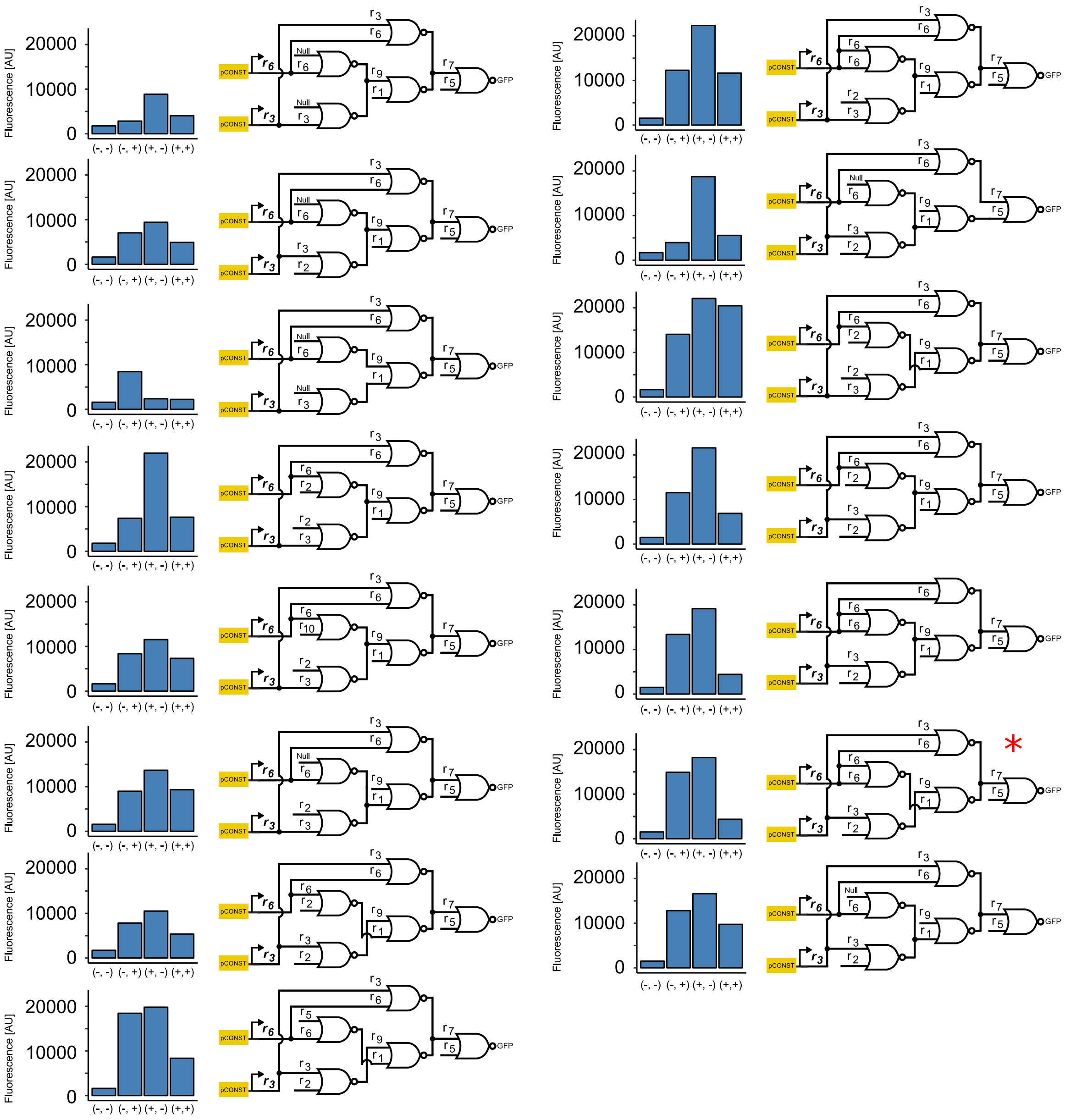

Supplementary Figure 9 | XOR circuit performance variation Fluorescence values for 15 different XOR circuit architectures containing different NOR gates exhibit variation in their output states. This illustrates the impact the performance of the component NOR gates have on overall circuit performance. The red asterisk indicates the XOR architecture that appears in figure 3e. Fluorescence values represent one measurement from cells in log phase.

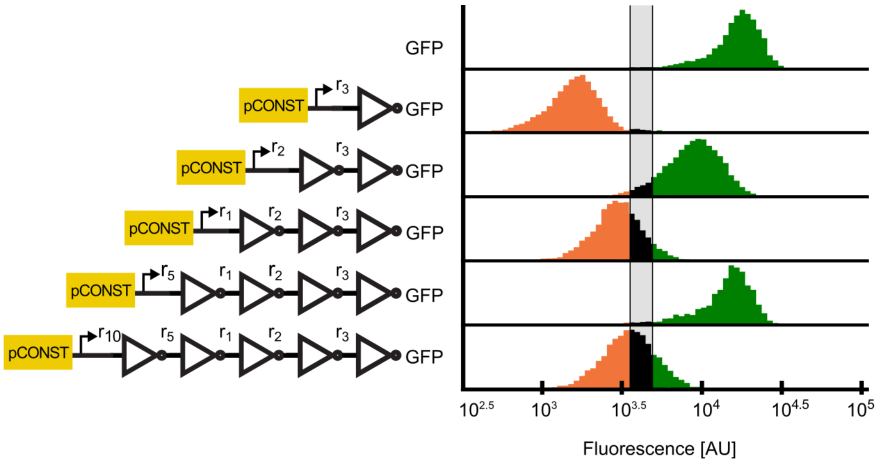

Supplementary Figure 10 | Additional repression cascades A 5-layer repression cascade. The histograms represent population fraction from three different biological replicates measured during a single experiment. Generally, additional layers added to the cascade decrease ON state fluorescence values and OFF state fluorescence values increase.

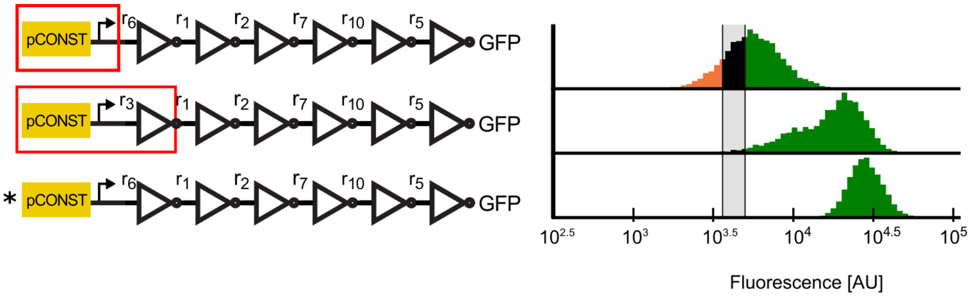

Supplementary Figure 11 | Six layer cascade comparison The fluorescence levels of two different 6 gRNA cascades are compared to the 6 gRNA cascade from figure 4, indicated by a *. The histograms represent population fraction from three different biological replicates measured during a single experiment. Differences in the composition of the cascades are highlighted in red. The pCONST promoter in the top most cascade is replaced with pGRR-r_3,r_{19}. The middle cascade has the pCONST promoter replaced with pGRR-r_9. The new pCONST is expressing gRNA-r_3 instead of gRNA-r_6. In addition, the promoter expressing gRNA-r_1 has been replaced with pGRR-r_3,r_3. Different combinations of promoters and gRNAs yield different levels of fluorescence in the ON state for these cascades. We hypothesize this is due to variations in the parameters associated with each gate, such as promoter strength and gRNA repression strength.

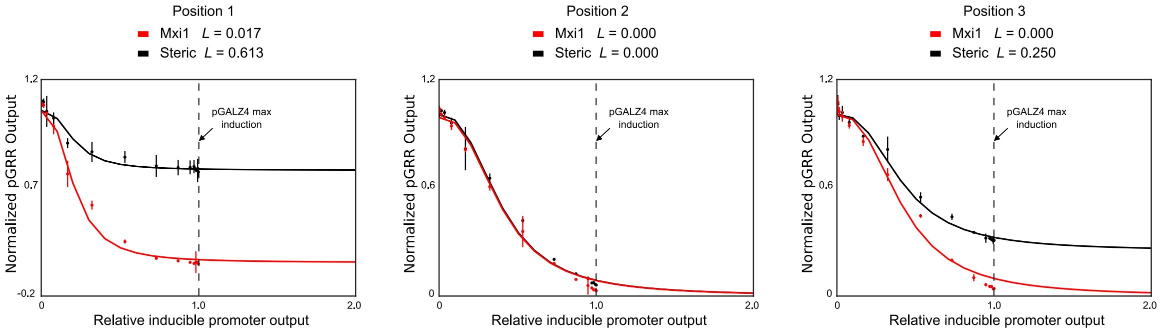

Supplementary Figure 12 | Alternative *dCas9-Mxi1* vs. *dCas9* repression comparison dose response curves Alternative plots of the *dCas9-Mxi1* vs. *dCas9* response curves from figure 2b are shown as a function of relative pGALZ4 inducible promoter output. A model fit of the pGALZ4 inducible promoter dose response function was used to scale the data. The maximum level of pGALZ4 induction is indicated on the plot. The model is extrapolated past the maximum induction level of pGALZ4 to observe the full behavior of the response curves. As the induction level of the inducible promoter goes to infinity the curves settle into an asymptote equivalent to the model predicted transcriptional leak parameter *L*.

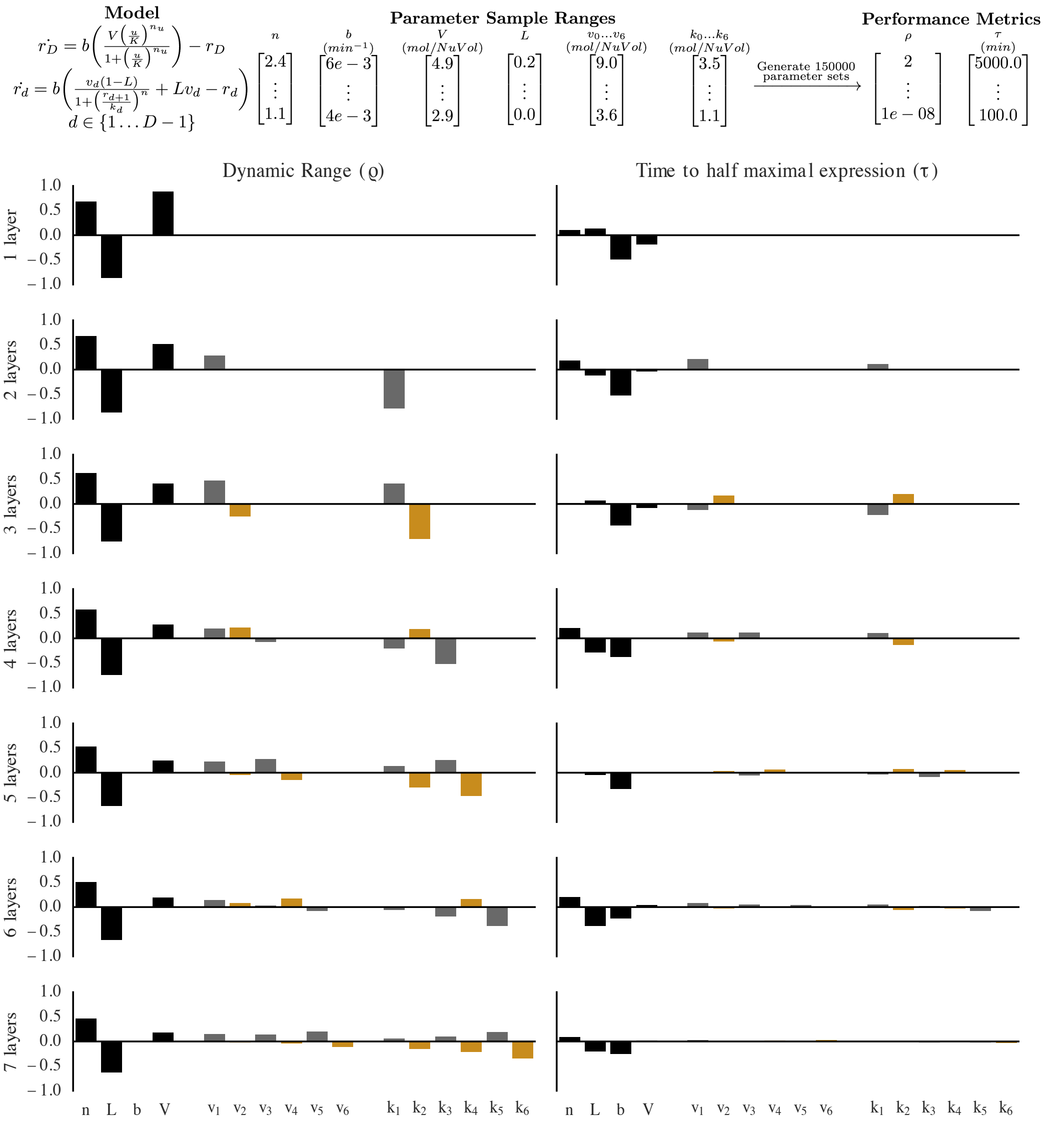

Supplementary Figure 13 | Model parameter sensitivity a 150,000 parameter sets were resampled from a uniform distribution over the intervals shown and applied to our repression cascade model (see methods). **b** Partial rank correlation coefficient (PRCC) was used to determine the contribution of each parameter has on either dynamic range or the time-to-half max. PRCCs were calculated using R (R Foundation for Statistical Computing, Vienna, Austria). Parameters associated with odd and even layers are colored grey and orange respectively. At all layers in the time-to-half maximal plot, *b* is very correlated with the output. In the dynamic range plot, *n* and *L* is strongly positively correlated at all layers with the output.

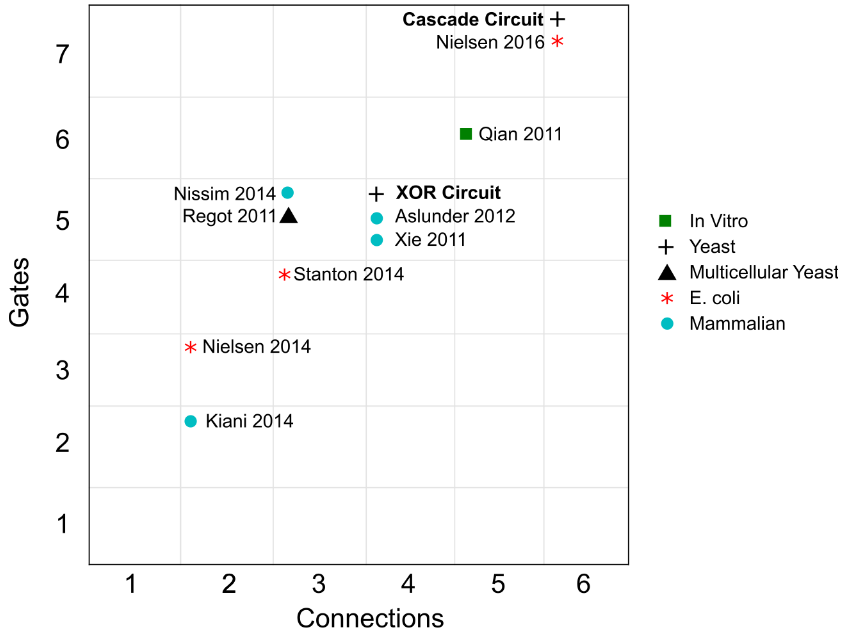

Supplementary Figure 14 | Synthetic circuit size comparison The best method for quantifying the size of synthetic biological circuits is an open question. Here we took the largest synthetic circuits constructed in nine[8-16] recent publications and compared them to the two largest circuits from this paper. We separated the inputs to the circuits from internal components. We also counted the number of connections between the internal components. By our definition, a "part" is a molecular species that carries information necessary for the internal function of the circuit (as opposed to a helper protein such as *dCas9*). A "connection" is a molecular interaction between parts that propagates information within the circuit.

Supplementary Table 1 | Guide sequence table This table lists all the guide sequences used in this work.

gRNA index	Sequence
r_1	GGAACGTGATTGAATAACTT
r_2	ACCAACGCAAAAAGATTTAG
r_3	CATTGCCATACACCTTGAGG
r_4	GAAAATCACAACTCTACTGA
r_5	GAAGTCAGTTGACAGAGTCG
r_6	GTGGTAACTTGCTCCATGTC
r_7	CTTTACGTATAGGTTTAGAG
r_8	CGCATTTCCTATTCAAACTT
r_9	GCAACCCACAAATATCCAGT
r_{10}	GTGACATAAACATTCGACTC
r_{11}	GGGCAAAGAGACGCTTGTCG
r_{12}	GAAGTCATCGCTTCTTGTCG
r_{13}	GAGTTGACAAAGTATAACTT
r_{14}	GAAGTTTCAGAATCTCGACG
r_{15}	GGCTAGGATCCATCTGACTT
r_{16}	GCAACCATAGACTCTCCAGG
r_{17}	ACCACAACTGAGTCGAACCT
r_{18}	GGGTAGCAACACTCGTACTT
r_{19}	GTAAAAGATAACTCTGTTGC
r_{20}	TCTACCCGAGACTCAAACGG
v_1	GTACATACAGTAGGATCCTA
v_2	TTTGGCACTACCGACACGAA
v_3	TGGTCAAAAGTGCGGCTTTC
v_4	CTTTCACAATCTTGACCTGC

Supplementary Table 2 | pCONST promoter table This table lists all constitutive promoter inputs for circuits built in this work.

**Strain**	**pConst**
Orthogonality Matrix Strains	pADH1:RGR_i
Figure 3 NOR	pADH1:RGR-r_7,pADH1:RGR-r_5
OR	pADH1:iRGR-r_3,pGRR:RGR-r_6
AND	pADH1:RGR-r_2,pGRR-r_5:RGR-r_1
NAND	pADH1:RGR-r_2,pGRR-nullnull:RGR-r_{10}
XOR	pADH1:iRGR-r_3,pGRR:RGR-r_6
XNOR	pAHD1:RGR-r_2,pGRR-nullnull:RGR-r_{10}
StaticCascade 1 Layer	pGRR-r_{10}:RGR-r_5
StaticCascade 2 Layer	pGRR-r_7:RGR-r_{10}
StaticCascade 3 Layer	pGRR-r_2:RGR-r_7
StaticCascade 4 Layer	pGRR-r1:RGR-r_2
StaticCascade 5 Layer	pGRR-r_6:RGR-r_1
StaticCascade 6 Layer	pGRR-r_3:RGR-r_6
StaticCascade 7 Layer	pGRR-r_9:RGR-r_3

Supplementary Table 3 | Interval population fractions of logic circuits This table displays the population fractions of the defined fluorescence intervals for all the static logic circuits appearing in the figure 3 and 4 and in Supplementary figures 10 and 11. A circuit's state was deemed acceptable if it met the specifications of having a majority of the cell population in the expected fluorescence interval.

NOR	(-,-)	(-,+)	(+,-)	(+,+)
ON population fraction	0.9910	0.0025	0.0028	0.0043
Undefined population fraction	0.0006	0.0025	0.0025	0.0012
OFF population fraction	0.0084	0.9951	0.9948	0.9944

OR	(-,-)	(-,+)	(+,-)	(+,+)
ON population fraction	0.0020	0.8799	0.9992	0.9923
Undefined population fraction	0.0027	0.0854	0.0004	0.0061
OFF population fraction	0.9953	0.0347	0.0004	0.0015

AND	(-,-)	(-,+)	(+,-)	(+,+)
ON population fraction	0.0177	0.0030	0.0044	0.9116
Undefined population fraction	0.0034	0.0045	0.0041	0.0642
OFF population fraction	0.9789	0.9924	0.9914	0.0242

NAND	(-,-)	(-,+)	(+,-)	(+,+)
ON population fraction	0.9560	0.8846	0.9179	0.2085
Undefined population fraction	0.0252	0.0614	0.0457	0.2047
OFF population fraction	0.0188	0.0540	0.0364	0.5868

XNOR	(-,-)	(-,+)	(+,-)	(+,+)
ON population fraction	0.9753	0.0468	0.0111	0.8768
Undefined population fraction	0.0184	0.0327	0.0308	0.0941
OFF population fraction	0.0063	0.9205	0.9581	0.0290

XOR	(-,-)	(-,+)	(+,-)	(+,+)
ON population fraction	0.0127	0.9786	0.9600	0.1337
Undefined population fraction	0.0029	0.0093	0.0060	0.1762
OFF population fraction	0.9844	0.0121	0.0341	0.6900

7 Layer Cascade	Layer 0	Layer 1	Layer 2	Layer 3	Layer 4	Layer 5	Layer 6	Layer 7
ON population fraction	0.9973	0.0448	0.9352	0.1262	0.7941	0.1978	0.9898	0.1680
Undefined population fraction	0.0004	0.1617	0.0500	0.2479	0.1421	0.2875	0.0032	0.3210
OFF population fraction	0.0022	0.7934	0.0148	0.6259	0.0638	0.5147	0.0069	0.5110

5 Layer Alternate Cascade	Layer 0	Layer 1	Layer 2	Layer 3	Layer 4	Layer 5
ON population fraction	0.9719	0.0219	0.9059	0.0732	0.9601	0.1844
Undefined population fraction	0.0141	0.0187	0.0653	0.2074	0.0222	0.2942
OFF population fraction	0.0139	0.9595	0.0288	0.7194	0.0177	0.5215

Alternate 6 Layer Cascades	Original	Alternate 1	Alternate 2
ON population fraction	0.9898	0.9969	0.6214
Undefined population fraction	0.0032	0.0019	0.2382
OFF population fraction	0.0069	0.0013	0.1403

Supplementary Table 4 | Parameter fit values Table describing parameter estimates for model from differential evolution for the steady-state and kinetics experiments. Using an estimated nuclear volume of ~6 μm^3 for diploid yeast[17] and the published dissociation constant of ~1.2nM for *Cas9* binding to its cognate site[18], bounds for parameters during optimization were selected based on estimates of transcription rates[19] and protein degradation rates in yeast[20]. Standard deviations of the steady-state parameters were determined from three independent experiments. Kinetic parameters were determined from a single experiment and do not have an estimate for experimental error.

parameter	mean	std	units	fitting bounds	description
V^{ss}	16.854	1.073	Molecule NucVol^{-1}	(0.434, 130.078)	Maximum transcription from inducible promoter
K	2.880	NA	nM	(2.880, 2.880)	Michaelis-Menten constant for βe inducible promoter
n_u	1.239	NA	dimensionless	(1.239, 1.239)	hill-coefficient for inducible promoter
v_0^{ss}	1.000	NA	AU	NA	Max fluorescence of reporter normalized to 1.0
v_1^{ss}	31.114	2.436	Molecule NucVol^{-1}	(0.434, 43.359)	Maximum transcription from pGRR promoter
v_2^{ss}	20.876	2.469	Molecule NucVol^{-1}	(0.434, 43.359)	Maximum transcription from pGRR promoter
v_3^{ss}	21.183	4.107	molecule/NucVol^{-1}	(0.434, 43.359)	Maximum transcription from pGRR promoter
k_0^{ss}	1.000	NA	Molecule NucVol^{-1}	NA	dissociation constant of gRNA-dCas9-Mxi1 to its cognate promoter
k_1^{ss}	6.129	0.992	Molecule NucVol^{-1}	(0.434, 43.359)	dissociation constant of gRNA-dCas9-Mxi1 to its cognate promoter
k_2^{ss}	12.229	4.065	Molecule NucVol^{-1}	(0.434, 43.359)	dissociation constant of gRNA-dCas9-Mxi1 to its cognate promoter
k_3^{ss}	11.782	3.442	Molecule NucVol^{-1}	(0.434, 43.359)	dissociation constant of gRNA-dCas9-Mxi1 to its cognate promoter
n^{ss}	1.882	0.107	dimensionless	(2.384, 2.385)	hill-coefficient
$V^{kinetics}$	12.923	0.019	Molecule NucVol^{-1}	(0.434, 130.078)	Maximum transcription from inducible promoter
$v_0^{kinetics}$	1.000	NA	AU	NA	Max fluorescence of reporter normalized to 1.0
$v_1^{kinetics}$	23.631	0.266	Molecule NucVol^{-1}	(0.434, 43.359)	Maximum transcription from pGRR promoter
$v_2^{kinetics}$	19.367	0.172	Molecule NucVol^{-1}	(0.434, 43.359)	Maximum transcription from pGRR promoter
$v_3^{kinetics}$	19.054	0.173	Molecule NucVol^{-1}	(0.434, 43.359)	Maximum transcription from pGRR promoter
$k_0^{kinetics}$	1.000	NA	Molecule NucVol^{-1}	NA	dissociation constant of gRNA-dCas9-Mxi1 to its cognate promoter
$k_1^{kinetics}$	6.771	0.072	Molecule NucVol^{-1}	(0.434, 43.359)	dissociation constant of gRNA-dCas9-Mxi1 to its cognate promoter
$k_2^{kinetics}$	12.988	0.061	Molecule NucVol^{-1}	(0.434, 43.359)	dissociation constant of gRNA-dCas9-Mxi1 to its cognate promoter
$k_3^{kinetics}$	14.411	0.121	Molecule NucVol^{-1}	(0.434, 43.359)	dissociation constant of gRNA-dCas9-Mxi1 to its cognate promoter
B	0.005	0.000	min^{-1}	(0.003, 0.011)	degradation/dilution of GFP reporter
b	0.006	0.000	min^{-1}	(0.003, 0.011)	degradation/dilution of gRNA-dCas9-Mxi1
$n^{kinetics}$	2.231	0.006	dimensionless	(0.500, 3.000)	hill-coefficient
Reported transcription rate	0.03 – 0.5	-	min^{-1}	-	transcription rate of most S. cerevisae promoters

Reported protein degradation rate	0.0001 9 – 0.058	-	min^{-1}	-	degradation rate of most S. cerevisae proteins
Kd of Cas9	1.200	-	nM	-	dissociation constant of gRNA-dCas9 to target measured *in vitro*

Supplementary References

1. Flick, JEFFREY S., and M. A. R. K. Johnston. "Two systems of glucose repression of the GAL1 promoter in Saccharomyces cerevisiae." *Molecular and Cellular Biology* 10.9 (1990): 4757-4769.

2. Witzgall, Ralph, et al. "The Krüppel-associated box-A (KRAB-A) domain of zinc finger proteins mediates transcriptional repression." *Proceedings of the National Academy of Sciences* 91.10 (1994): 4514-4518.

3. Sridhar, Vaniyambadi V., et al. "Transcriptional repression of target genes by LEUNIG and SEUSS, two interacting regulatory proteins for Arabidopsis flower development." *Proceedings of the National Academy of Sciences of the United States of America* 101.31 (2004): 11494-11499.

4. Gilbert, Luke A., et al. "CRISPR-mediated modular RNA-guided regulation of transcription in eukaryotes." *Cell* 154.2 (2013): 442-451.

5. Pierre-Jerome, Edith, et al. "Recapitulation of the forward nuclear auxin response pathway in yeast." *Proceedings of the National Academy of Sciences* 111.26 (2014): 9407-9412.

6. Wu, Jiansheng, et al. "TUP1 utilizes histone H3/H2B–specific HDA1 deacetylase to repress gene activity in yeast." *Molecular cell* 7.1 (2001): 117-126.

7. Traven, Ana, et al. "The yeast protein Xtc1 functions as a direct transcriptional repressor." *Nucleic acids research* 30.11 (2002): 2358-2364.

8. Kiani, Samira, et al. "CRISPR transcriptional repression devices and layered circuits in mammalian cells." *Nature methods* 11.7 (2014): 723-726.

9. Xie, Zhen, et al. "Multi-input RNAi-based logic circuit for identification of specific cancer cells." *Science* 333.6047 (2011): 1307-1311.

10. Ausländer, Simon, et al. "Programmable single-cell mammalian biocomputers." *Nature* 487.7405 (2012): 123-127.

11. Nissim, Lior, et al. "Multiplexed and programmable regulation of gene networks with an integrated RNA and CRISPR/Cas toolkit in human cells."*Molecular cell* 54.4 (2014): 698-710.

12. Nielsen, Alec AK, et al. "Genetic circuit design automation." *Science*352.6281 (2016): aac7341.

13. Qian, Lulu, and Erik Winfree. "Scaling up digital circuit computation with DNA strand displacement cascades." *Science* 332.6034 (2011): 1196-1201.

14. Nielsen, Alec AK, and Christopher A. Voigt. "Multi‐input CRISPR/Cas genetic circuits that interface host regulatory networks." *Molecular systems biology*10.11 (2014): 763.

15. Regot, Sergi, et al. "Distributed biological computation with multicellular engineered networks." *Nature* 469.7329 (2011): 207-211.

16. Stanton, Brynne C., et al. "Genomic mining of prokaryotic repressors for orthogonal logic gates." *Nature chemical biology* 10.2 (2014): 99-105.

17. Jorgensen P, Edgington NP, Schneider BL, Rupes I, Tyers M, Futcher B. The size of the nucleus increases as yeast cells grow. Mol Biol Cell. 2007 Sep18(9):3523-32.

18. Richardson, C., Ray, G., DeWitt, M., Curie, G. & Corn, J. Enhancing homology-directed genome editing by catalytically active and inactive CRISPR-Cas9 using asymmetric donor DNA. Nat Biotechnol 34, 339–344 (2016).

19. Transcription rate of most S. cerevisae promoters [REF Pelechano, V., Chávez, S. & Pérez-Ortín, J. E. E. A complete set of nascent transcription rates for yeast genes. PLoS ONE 5, e15442 (2010).

20. Christiano, Romain, et al. "Global proteome turnover analyses of the yeasts S. cerevisiae and S. pombe." *Cell reports* 9.5 (2014): 1959-1965.
