# Supplementary figures and images for: Digital logic circuits in yeast with CRISPR-dCas9 NOR gates
Source: Nat Commun. 2017 May 25;8:15459. doi: 10.1038/ncomms15459 (PMC5458518; doi:10.1038/ncomms15459)

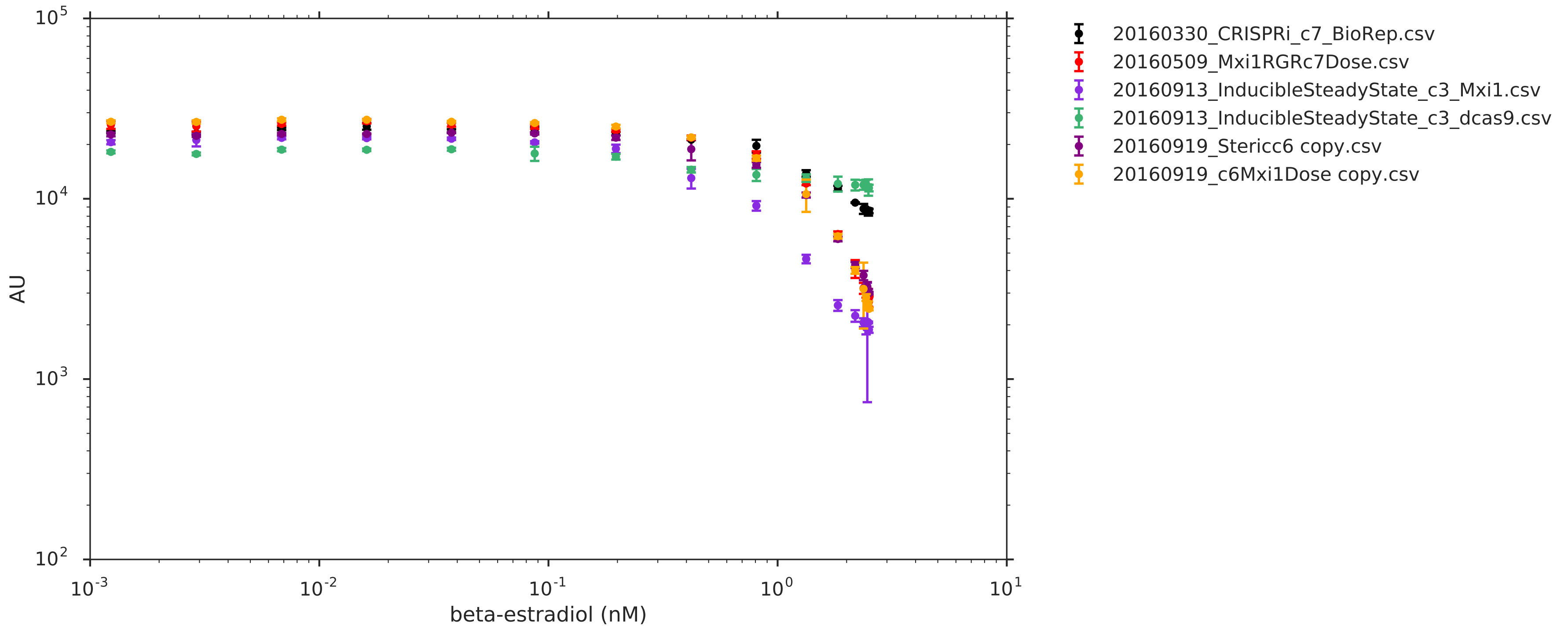

Supplement: Supplementary Software 1 — R cytometry data processing scripts and mathematical modeling scripts [file ncomms15459-s3.zip › Supplementary Software 1/FittingScripts/CRISPRi_Fitting/FinalResults/20160919_c6Mxi1Dose copy.csv_CRISPRi_fits.pdf]

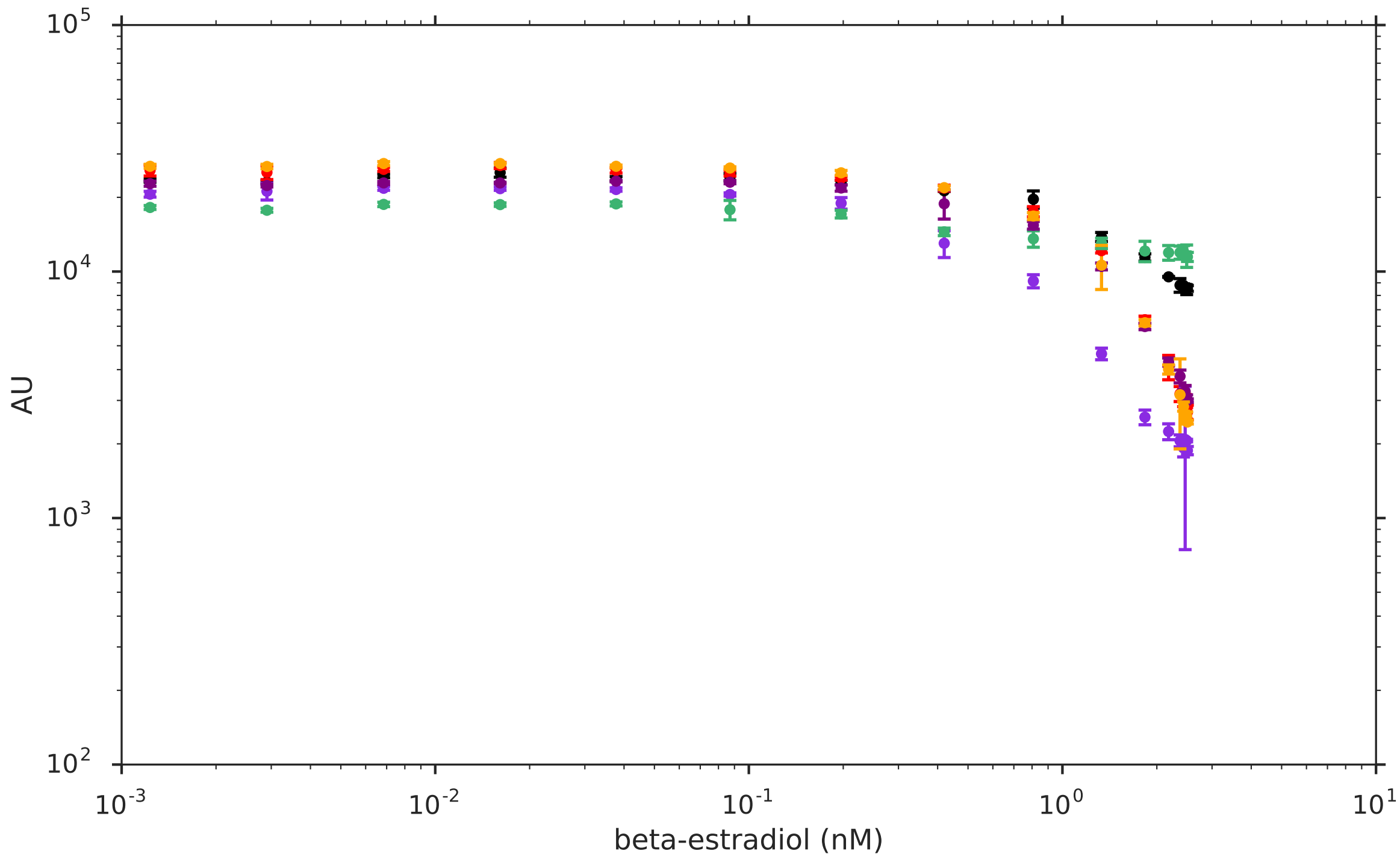

Supplement: Supplementary Software 1 — R cytometry data processing scripts and mathematical modeling scripts [file ncomms15459-s3.zip › Supplementary Software 1/FittingScripts/CRISPRi_Fitting/FinalResults/crispri_plot.pdf]

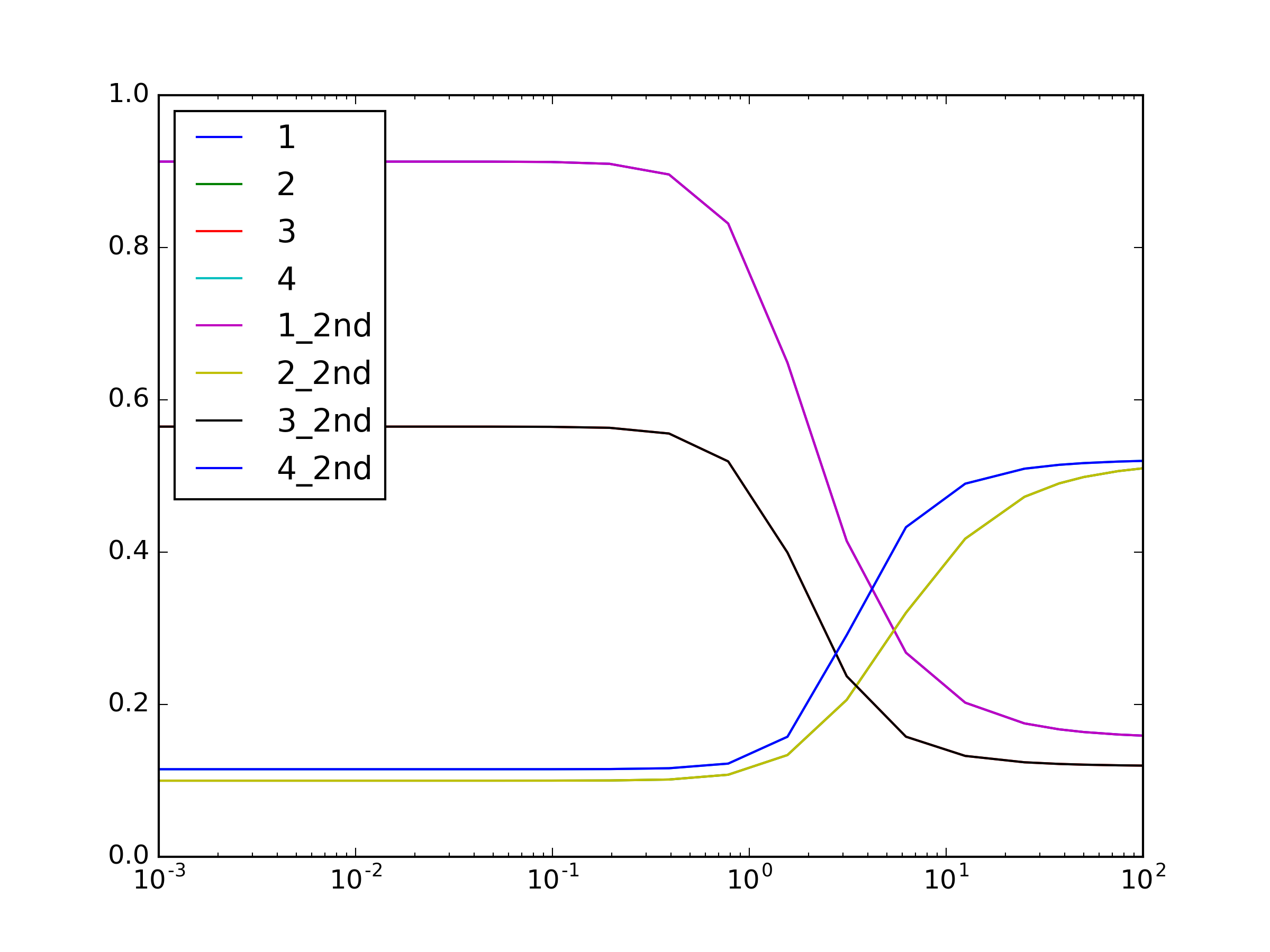

Supplement: Supplementary Software 1 — R cytometry data processing scripts and mathematical modeling scripts [file ncomms15459-s3.zip › Supplementary Software 1/FittingScripts/Results/Output/example_input_dose_1_steadystate_2016-04-15-11-34-29_1460745269611107.png]

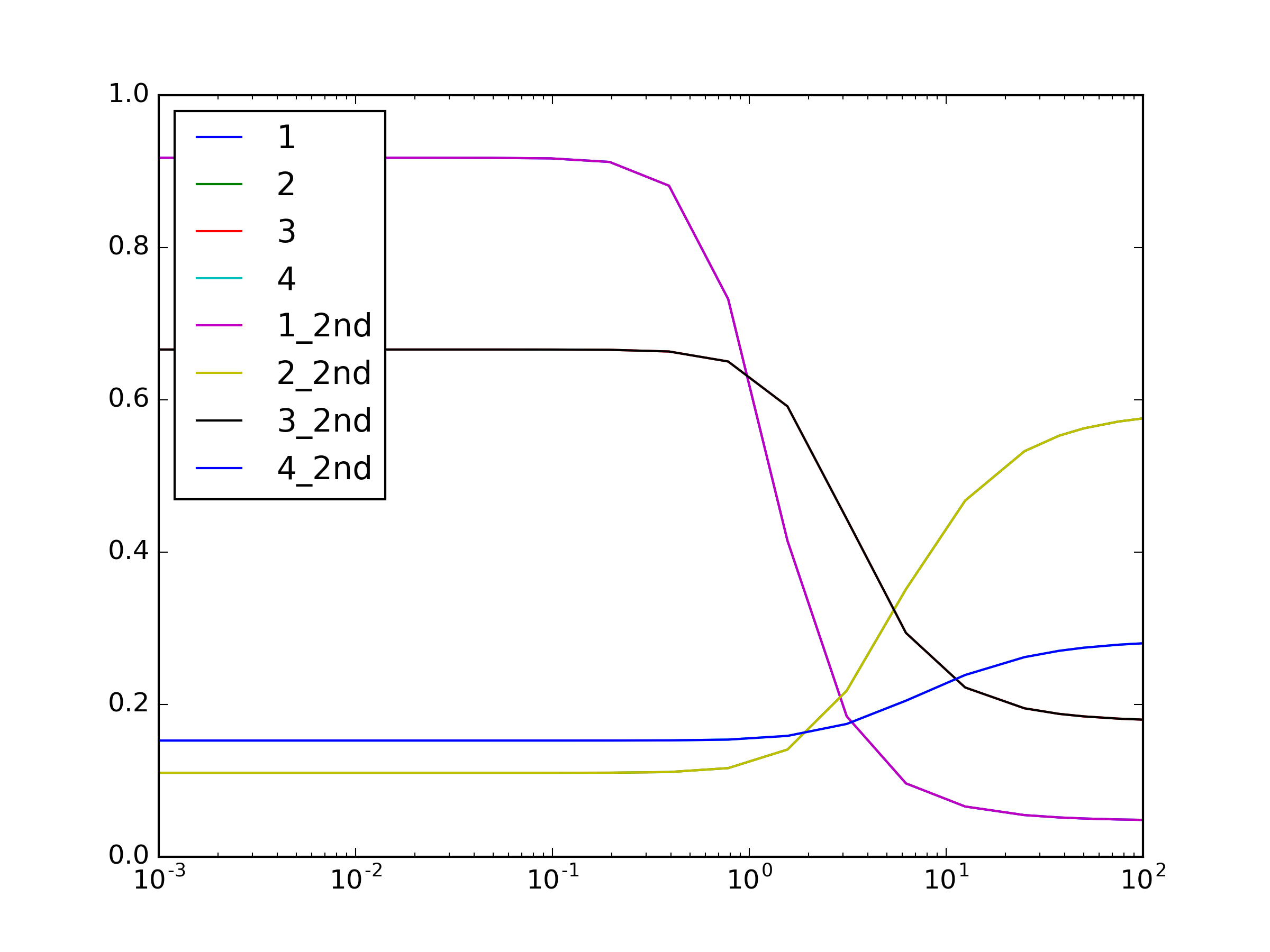

Supplement: Supplementary Software 1 — R cytometry data processing scripts and mathematical modeling scripts [file ncomms15459-s3.zip › Supplementary Software 1/FittingScripts/Results/Output/example_input_dose_1_steadystate_2016-04-15-11-35-48_1460745348613042.png]

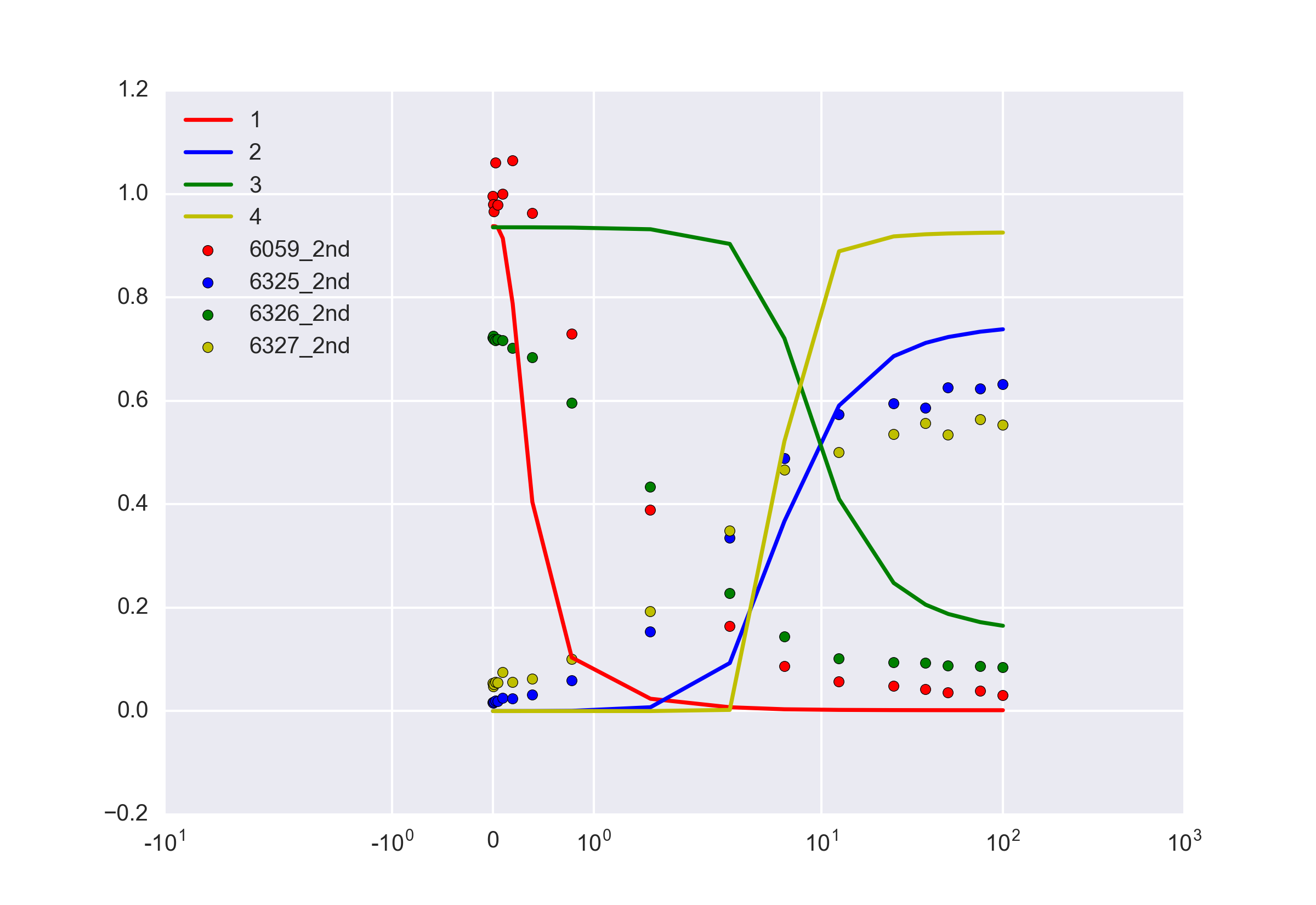

Supplement: Supplementary Software 1 — R cytometry data processing scripts and mathematical modeling scripts [file ncomms15459-s3.zip › Supplementary Software 1/FittingScripts/Results/Output/exp1_dose_0_steadystate_2016-04-15-23-35-13_1460788513529432.png]

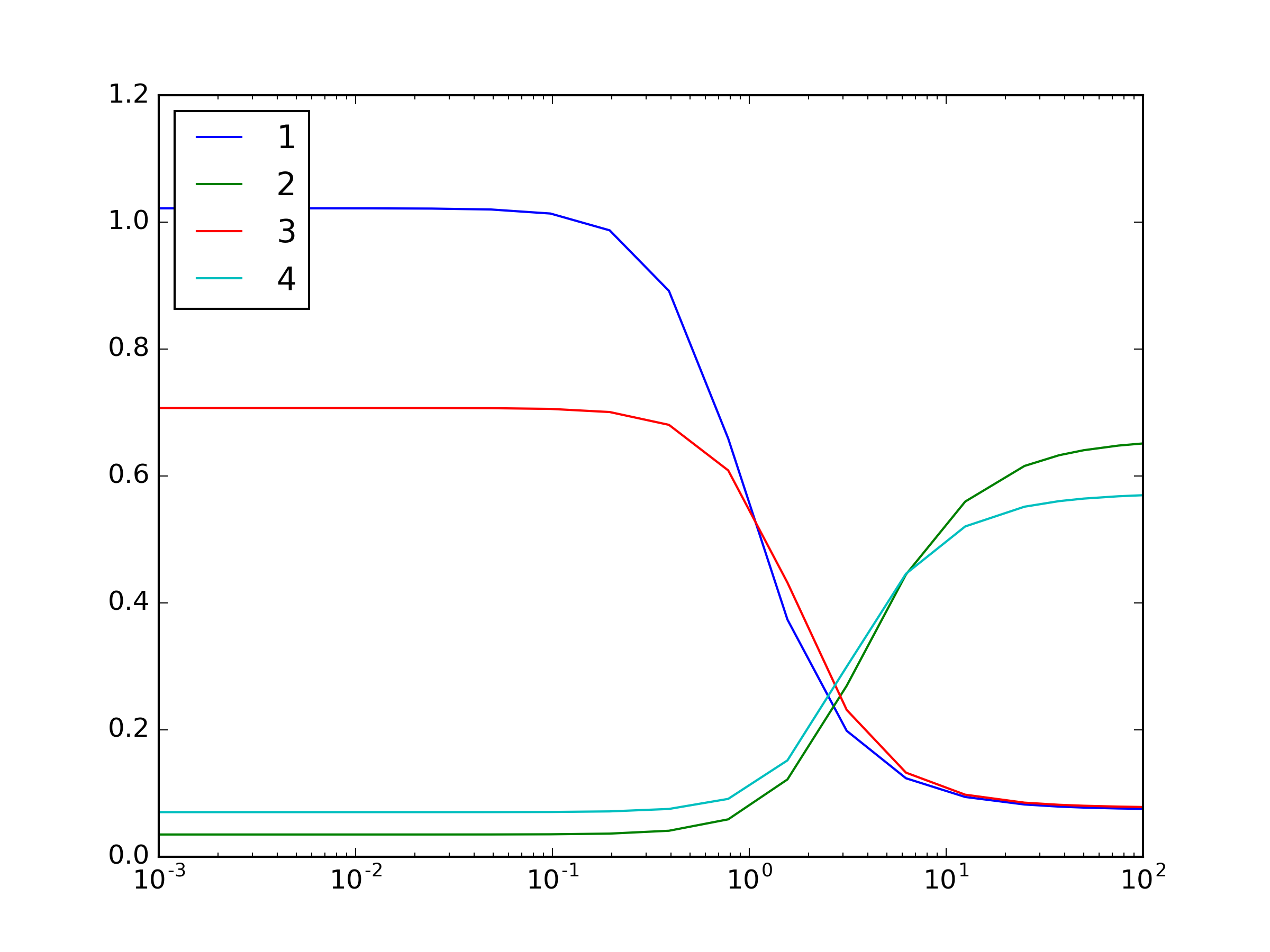

Supplement: Supplementary Software 1 — R cytometry data processing scripts and mathematical modeling scripts [file ncomms15459-s3.zip › Supplementary Software 1/FittingScripts/Results/Output/FittingScript_DoseExp1_20160330.py_model_image_2016-04-01-12-26-42_1459538802564563.png]

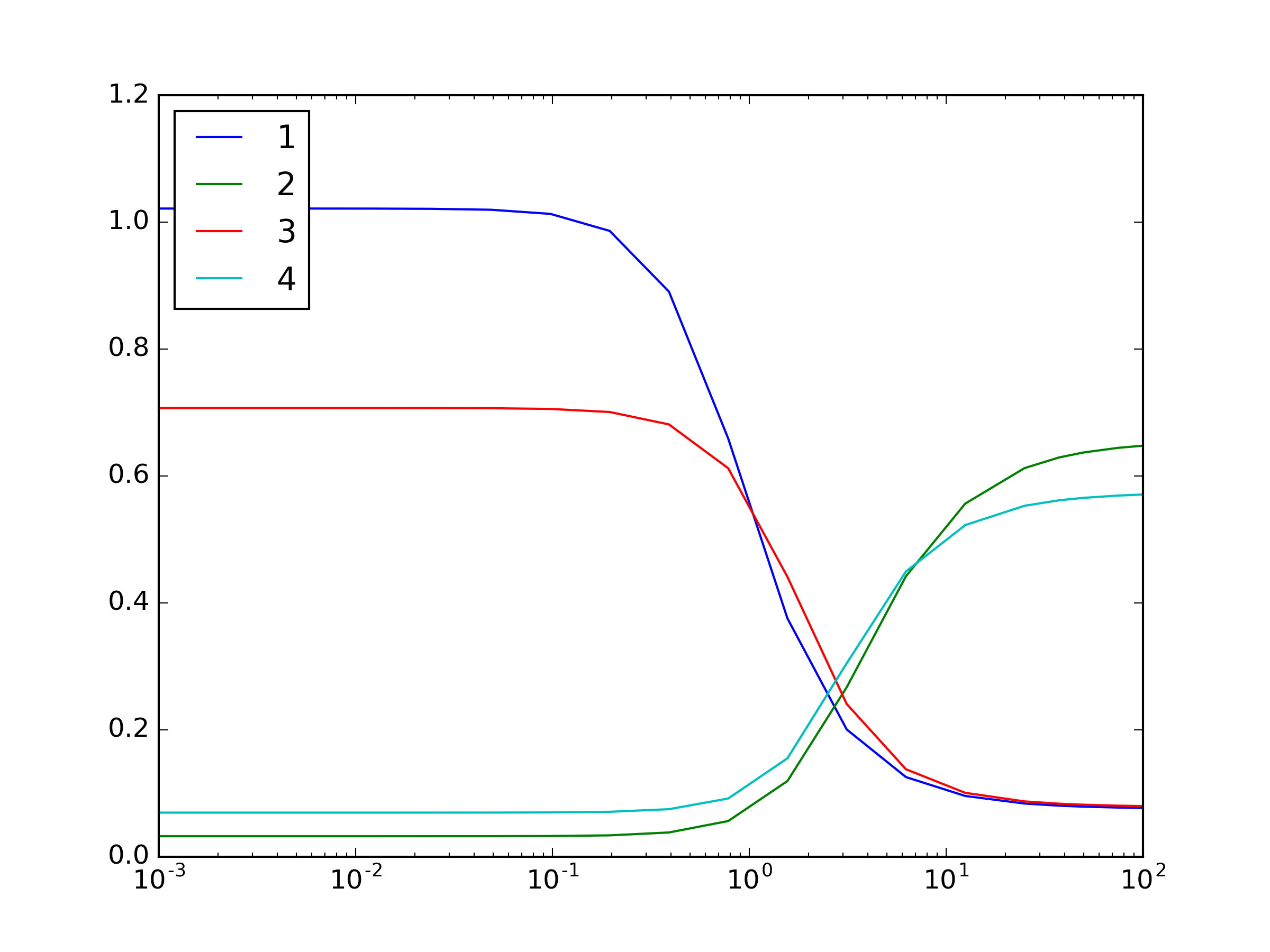

Supplement: Supplementary Software 1 — R cytometry data processing scripts and mathematical modeling scripts [file ncomms15459-s3.zip › Supplementary Software 1/FittingScripts/Results/Output/FittingScript_DoseExp1_20160330.py_model_image_2016-04-01-18-58-20_1459562300424089.png]

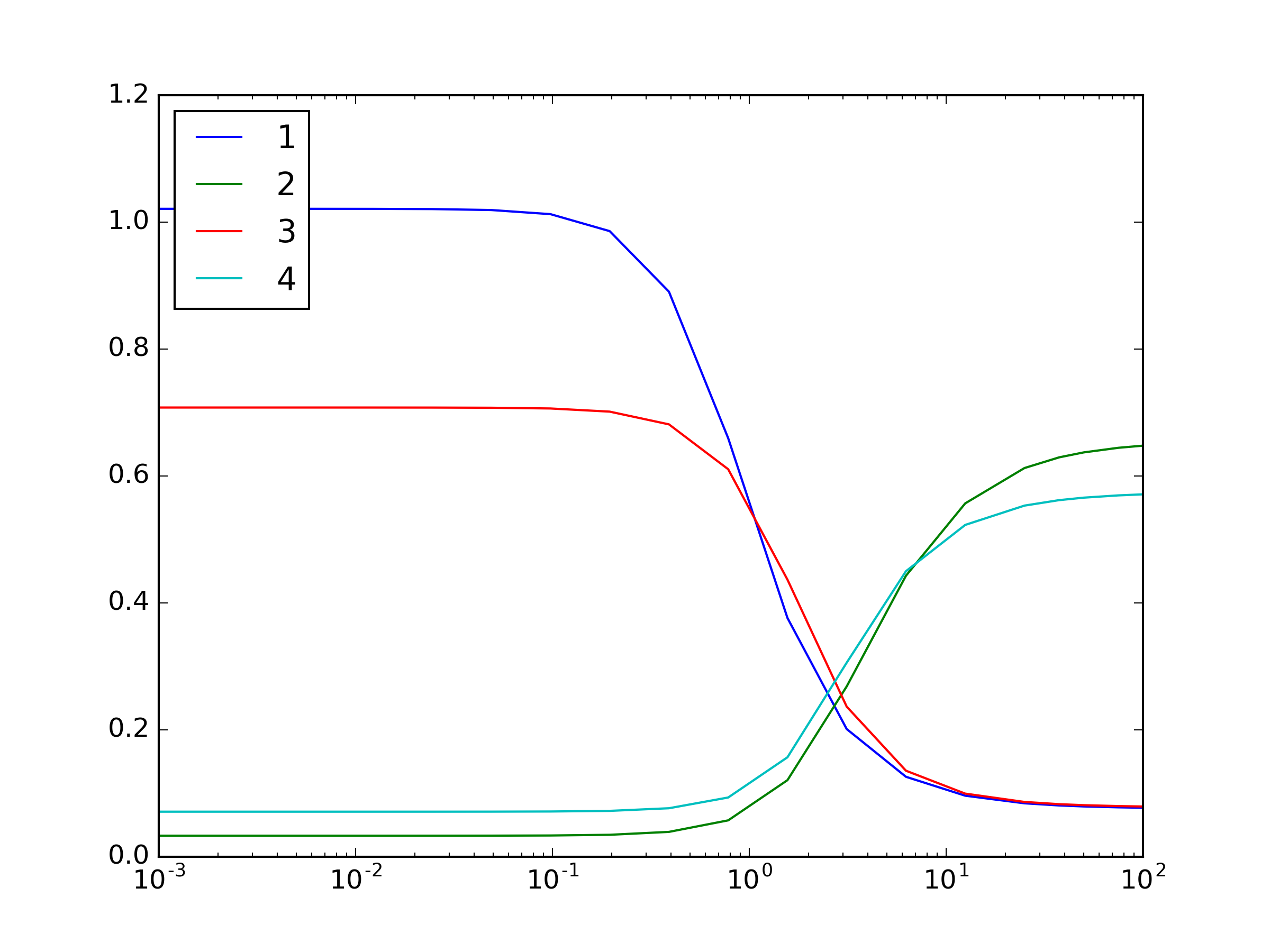

Supplement: Supplementary Software 1 — R cytometry data processing scripts and mathematical modeling scripts [file ncomms15459-s3.zip › Supplementary Software 1/FittingScripts/Results/Output/FittingScript_DoseExp1_20160330.py_model_image_2016-04-01-21-19-15_1459570755367534.png]

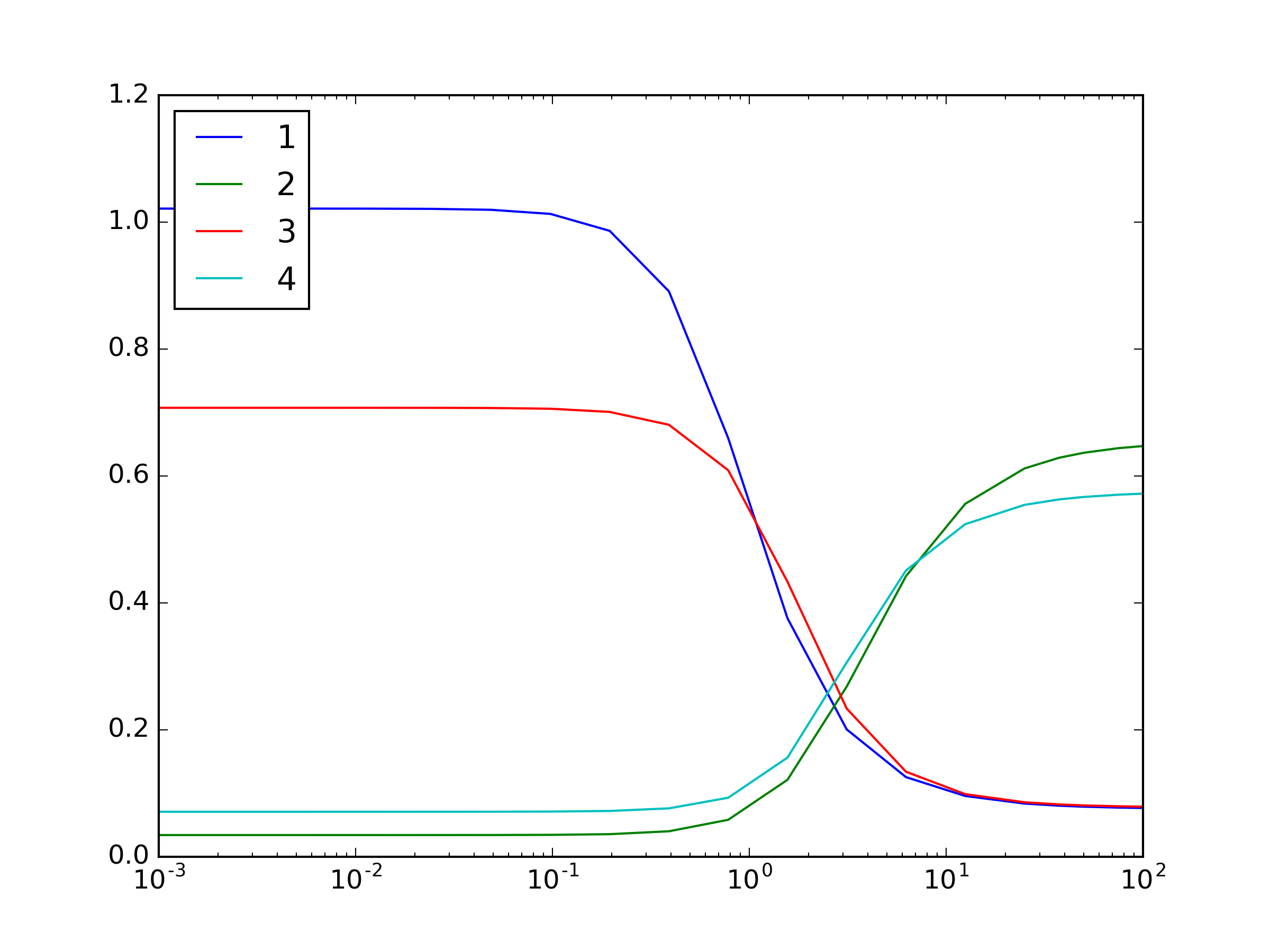

Supplement: Supplementary Software 1 — R cytometry data processing scripts and mathematical modeling scripts [file ncomms15459-s3.zip › Supplementary Software 1/FittingScripts/Results/Output/FittingScript_DoseExp1_20160330.py_model_image_2016-04-01-23-46-11_1459579571630960.png]

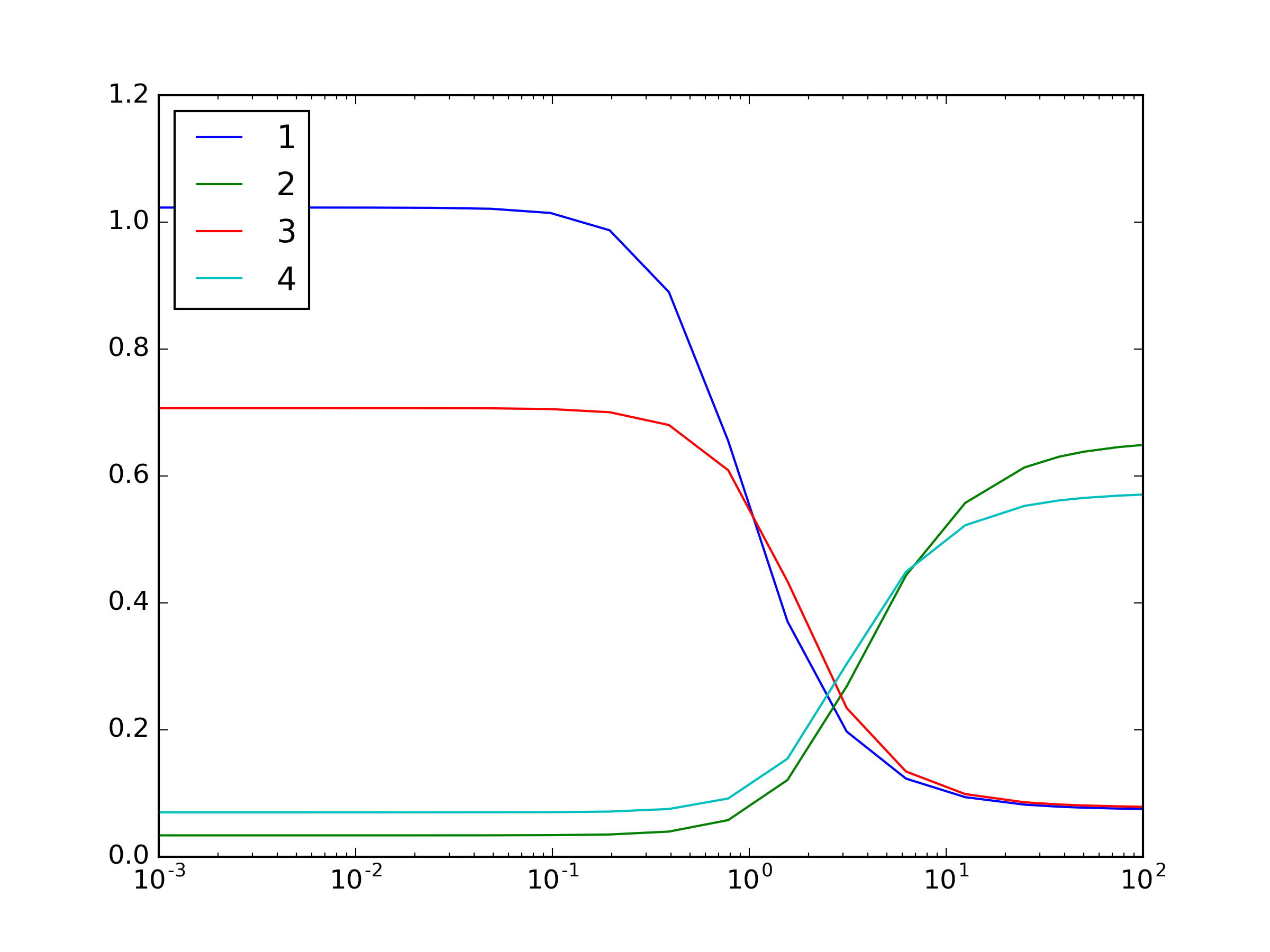

Supplement: Supplementary Software 1 — R cytometry data processing scripts and mathematical modeling scripts [file ncomms15459-s3.zip › Supplementary Software 1/FittingScripts/Results/Output/FittingScript_DoseExp1_20160330.py_model_image_2016-04-02-01-41-46_1459586506981830.png]

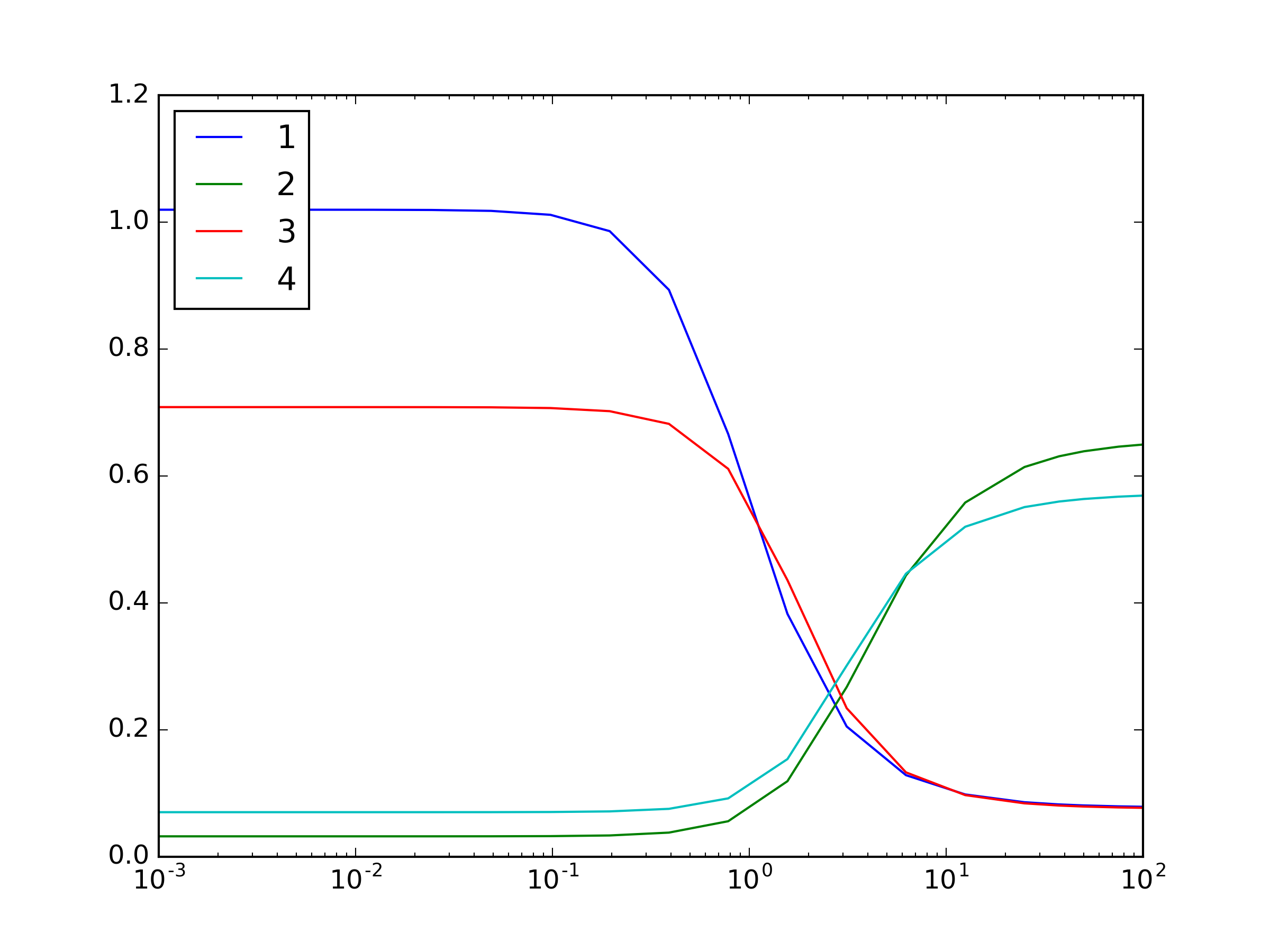

Supplement: Supplementary Software 1 — R cytometry data processing scripts and mathematical modeling scripts [file ncomms15459-s3.zip › Supplementary Software 1/FittingScripts/Results/Output/FittingScript_DoseExp1_20160330.py_model_image_2016-04-02-03-49-26_1459594166877957.png]

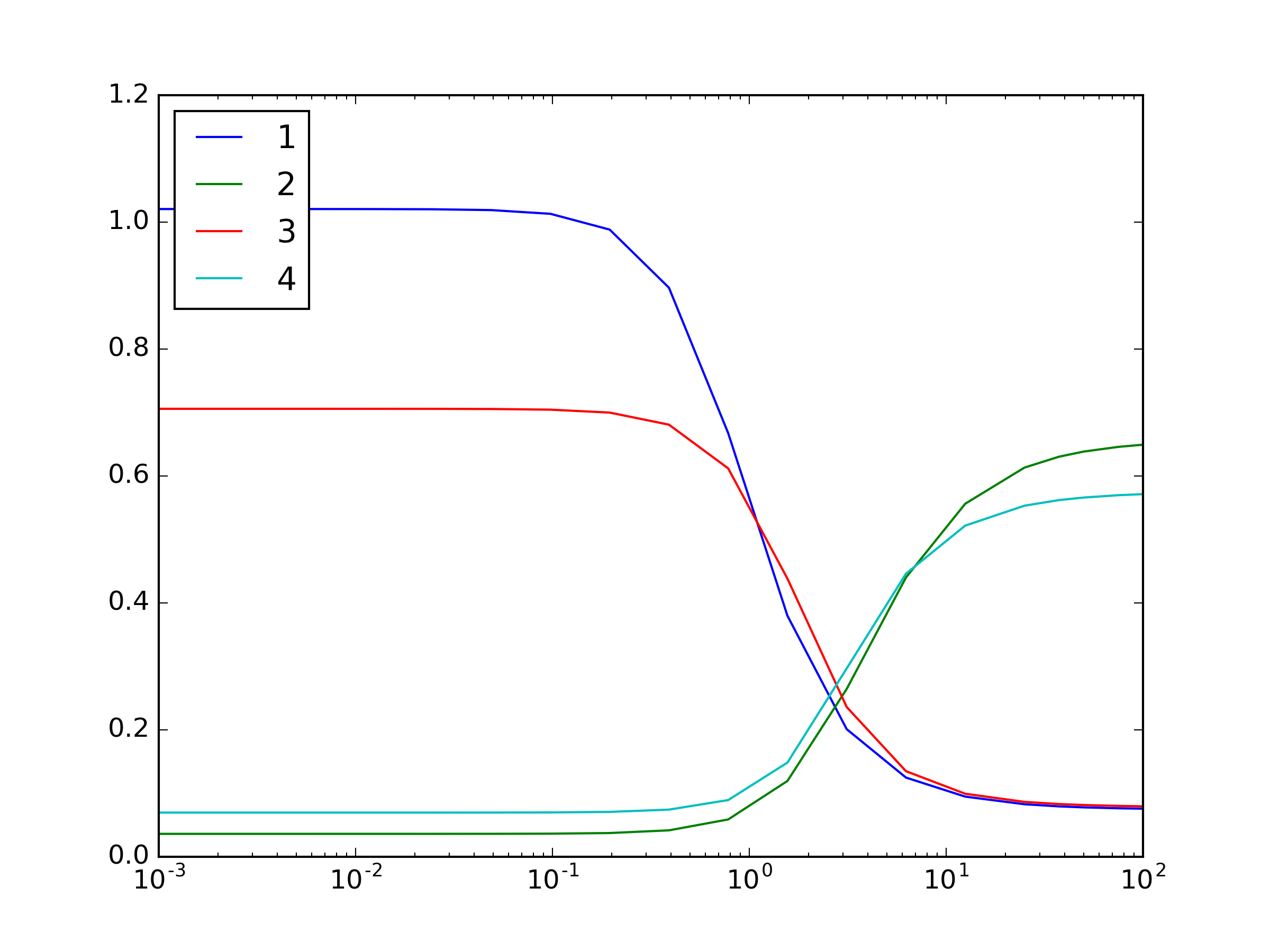

Supplement: Supplementary Software 1 — R cytometry data processing scripts and mathematical modeling scripts [file ncomms15459-s3.zip › Supplementary Software 1/FittingScripts/Results/Output/FittingScript_DoseExp1_20160330.py_model_image_2016-04-02-06-20-04_1459603204672098.png]

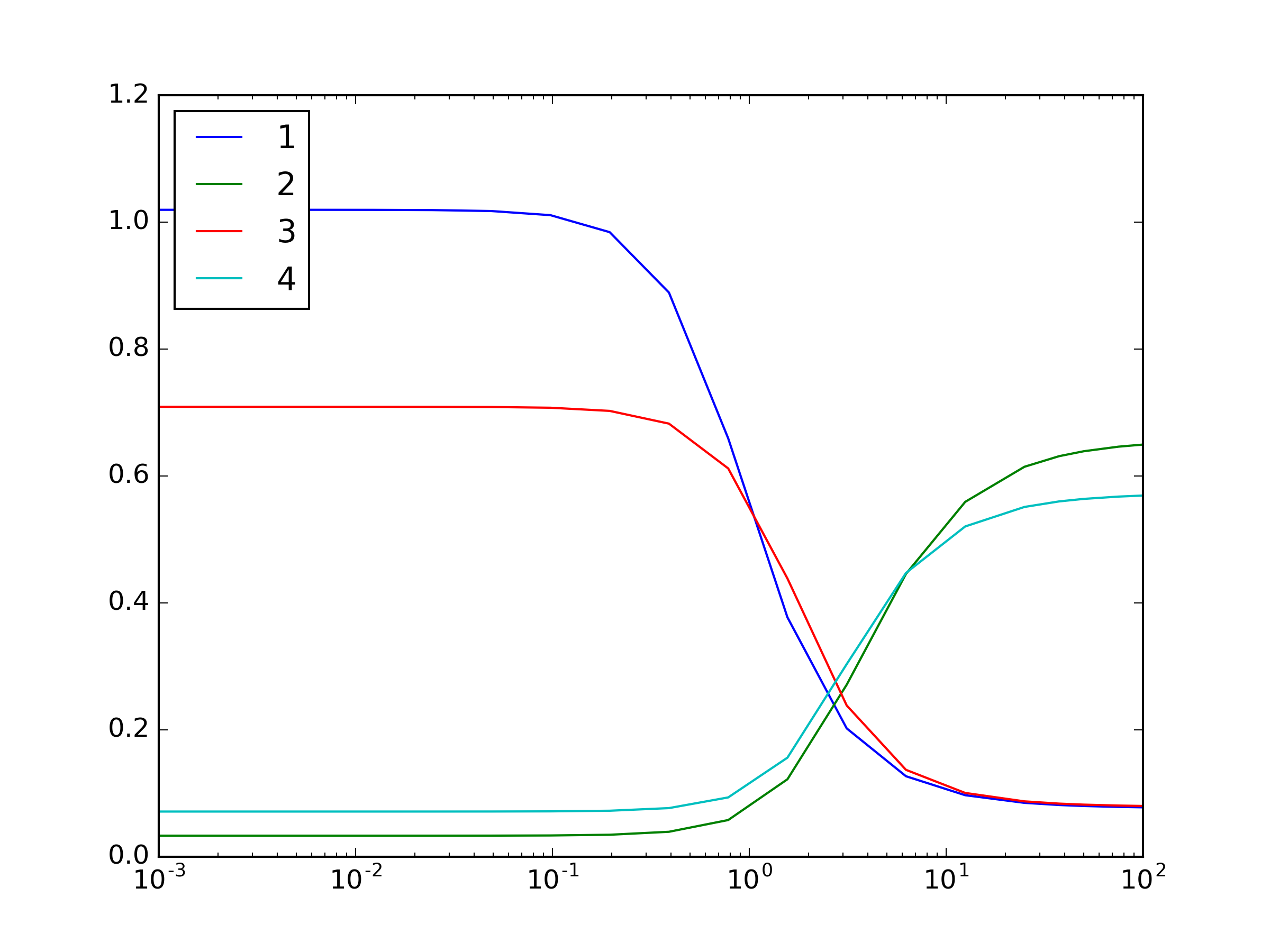

Supplement: Supplementary Software 1 — R cytometry data processing scripts and mathematical modeling scripts [file ncomms15459-s3.zip › Supplementary Software 1/FittingScripts/Results/Output/FittingScript_DoseExp1_20160330.py_model_image_2016-04-02-08-37-33_1459611453428725.png]

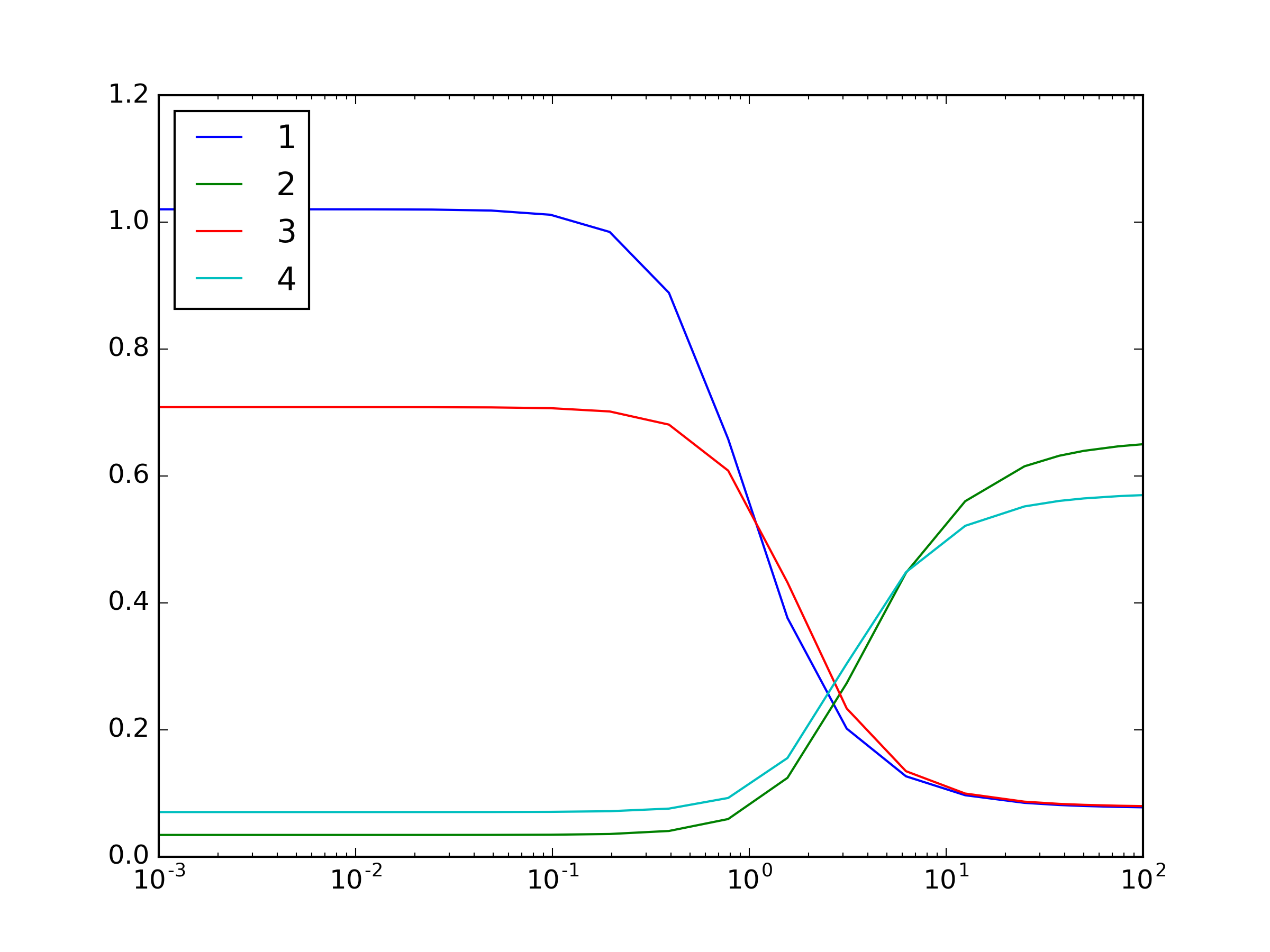

Supplement: Supplementary Software 1 — R cytometry data processing scripts and mathematical modeling scripts [file ncomms15459-s3.zip › Supplementary Software 1/FittingScripts/Results/Output/FittingScript_DoseExp1_20160330.py_model_image_2016-04-02-11-29-58_1459621798490186.png]

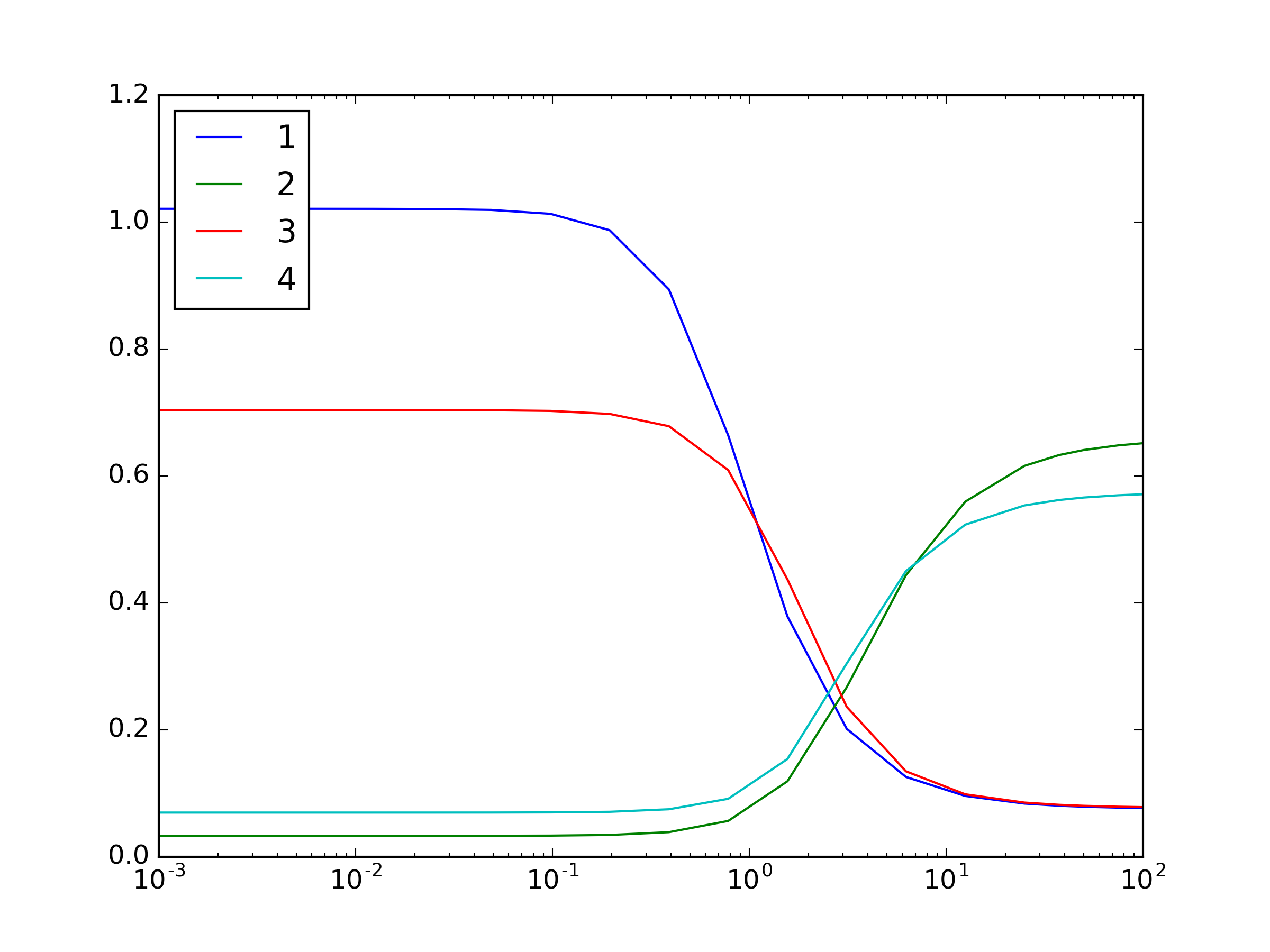

Supplement: Supplementary Software 1 — R cytometry data processing scripts and mathematical modeling scripts [file ncomms15459-s3.zip › Supplementary Software 1/FittingScripts/Results/Output/FittingScript_DoseExp1_20160330.py_model_image_2016-04-02-14-11-07_1459631467556026.png]

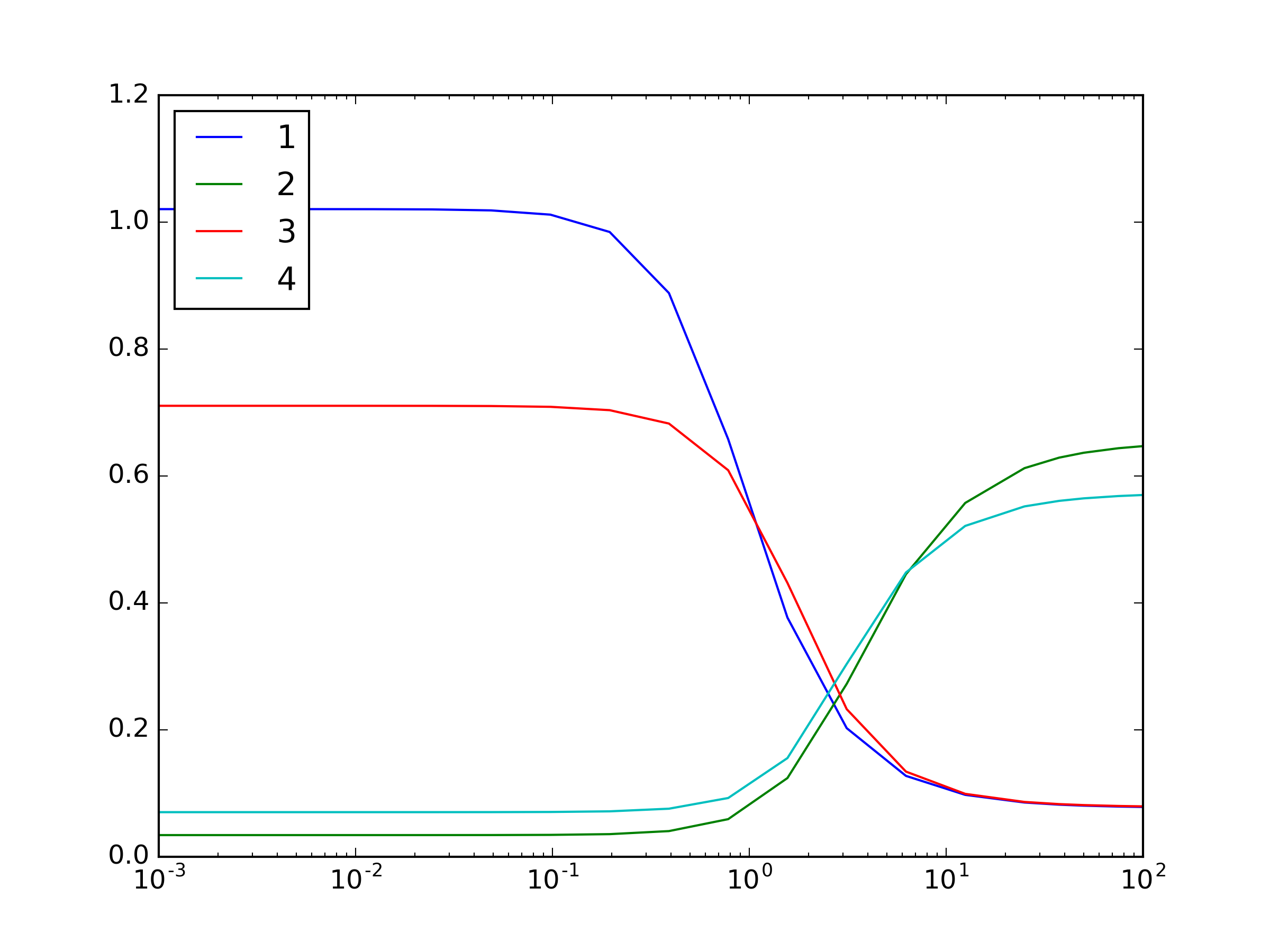

Supplement: Supplementary Software 1 — R cytometry data processing scripts and mathematical modeling scripts [file ncomms15459-s3.zip › Supplementary Software 1/FittingScripts/Results/Output/FittingScript_DoseExp1_20160330.py_model_image_2016-04-02-16-33-52_1459640032021555.png]

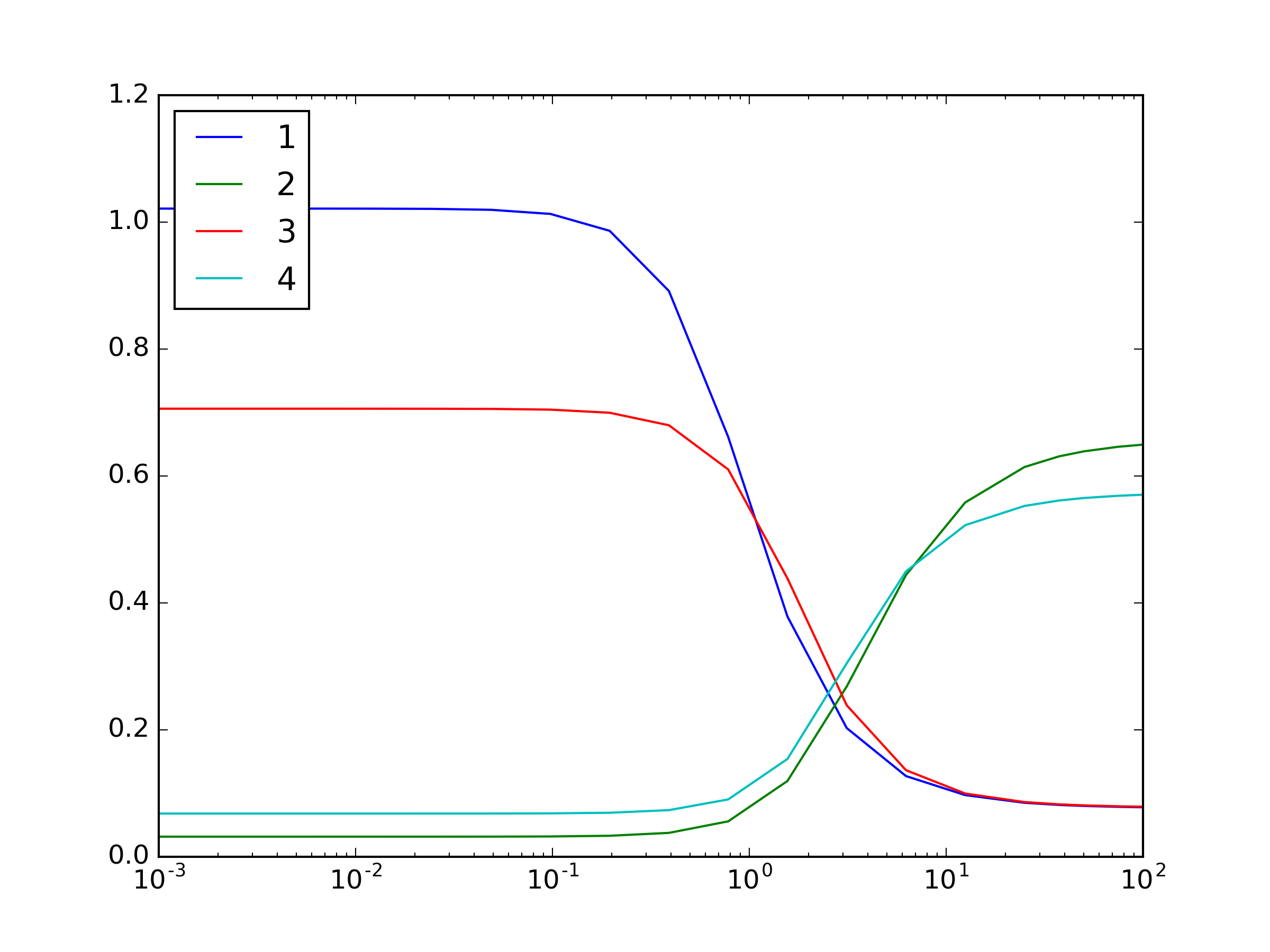

Supplement: Supplementary Software 1 — R cytometry data processing scripts and mathematical modeling scripts [file ncomms15459-s3.zip › Supplementary Software 1/FittingScripts/Results/Output/FittingScript_DoseExp1_20160330.py_model_image_2016-04-02-20-07-13_1459652833807953.png]

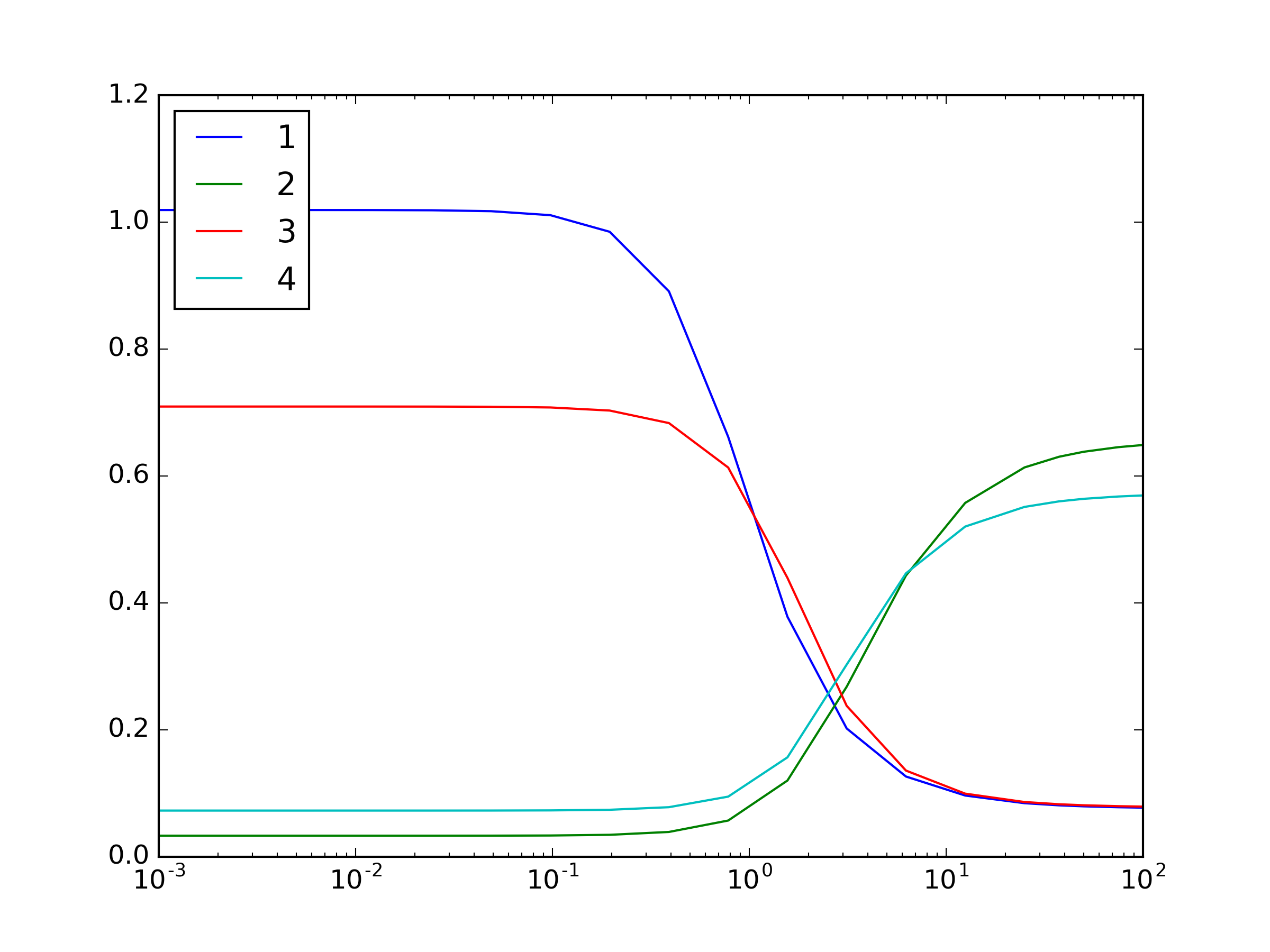

Supplement: Supplementary Software 1 — R cytometry data processing scripts and mathematical modeling scripts [file ncomms15459-s3.zip › Supplementary Software 1/FittingScripts/Results/Output/FittingScript_DoseExp1_20160330.py_model_image_2016-04-02-22-13-55_1459660435959097.png]

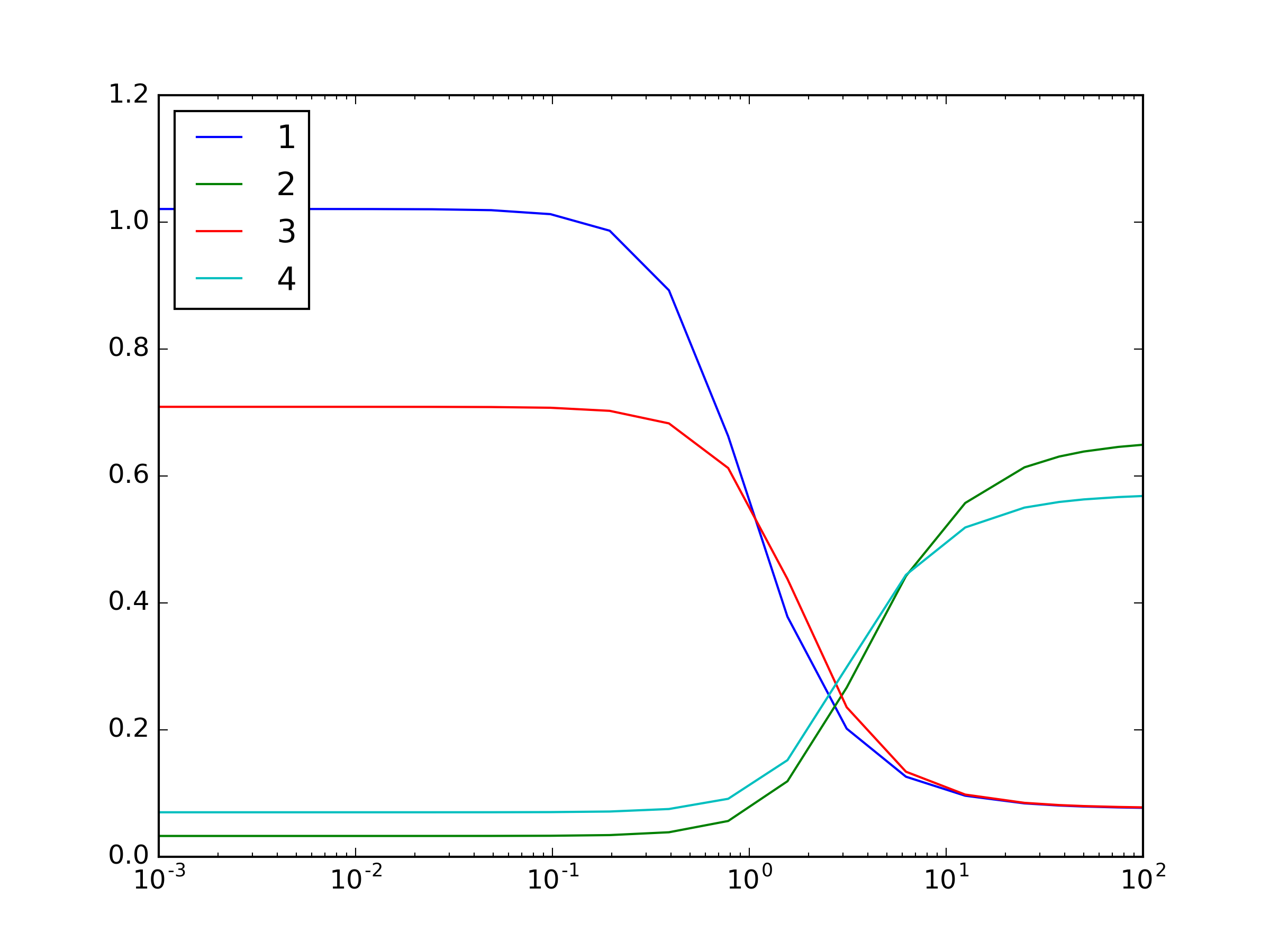

Supplement: Supplementary Software 1 — R cytometry data processing scripts and mathematical modeling scripts [file ncomms15459-s3.zip › Supplementary Software 1/FittingScripts/Results/Output/FittingScript_DoseExp1_20160330.py_model_image_2016-04-03-00-33-53_1459668833315539.png]

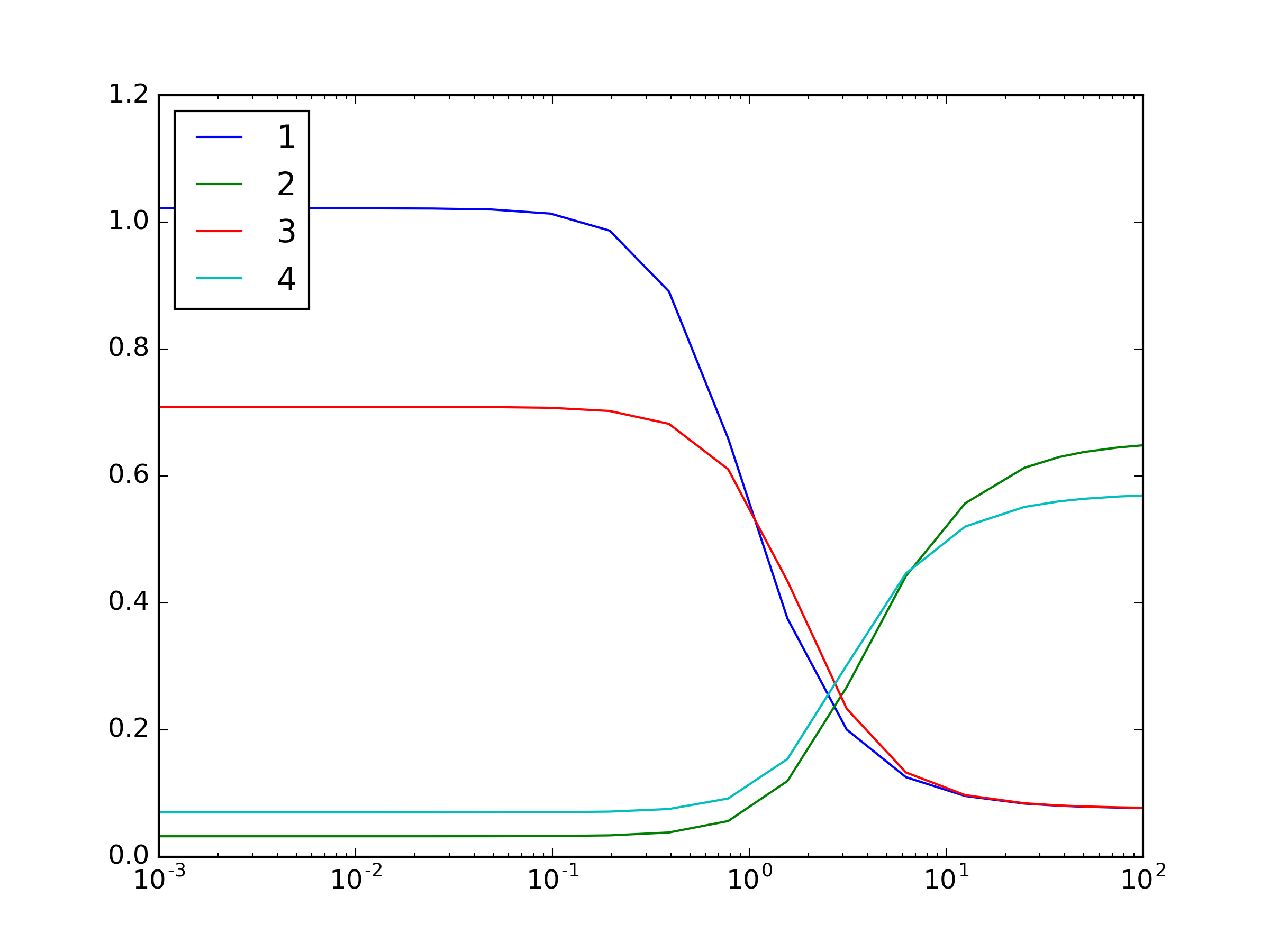

Supplement: Supplementary Software 1 — R cytometry data processing scripts and mathematical modeling scripts [file ncomms15459-s3.zip › Supplementary Software 1/FittingScripts/Results/Output/FittingScript_DoseExp1_20160330.py_model_image_2016-04-03-02-46-39_1459676799238535.png]

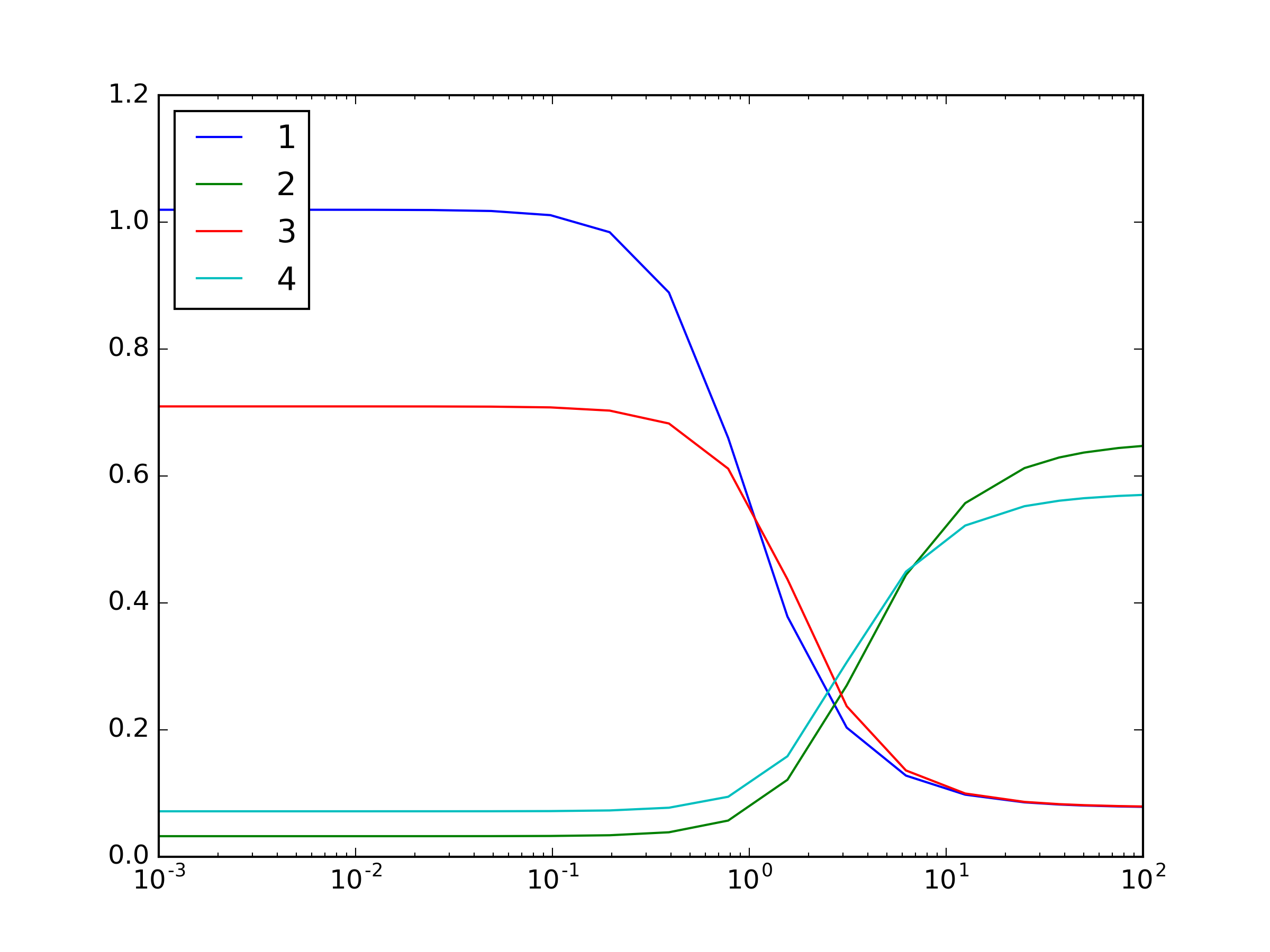

Supplement: Supplementary Software 1 — R cytometry data processing scripts and mathematical modeling scripts [file ncomms15459-s3.zip › Supplementary Software 1/FittingScripts/Results/Output/FittingScript_DoseExp1_20160330.py_model_image_2016-04-03-05-27-32_1459686452245637.png]

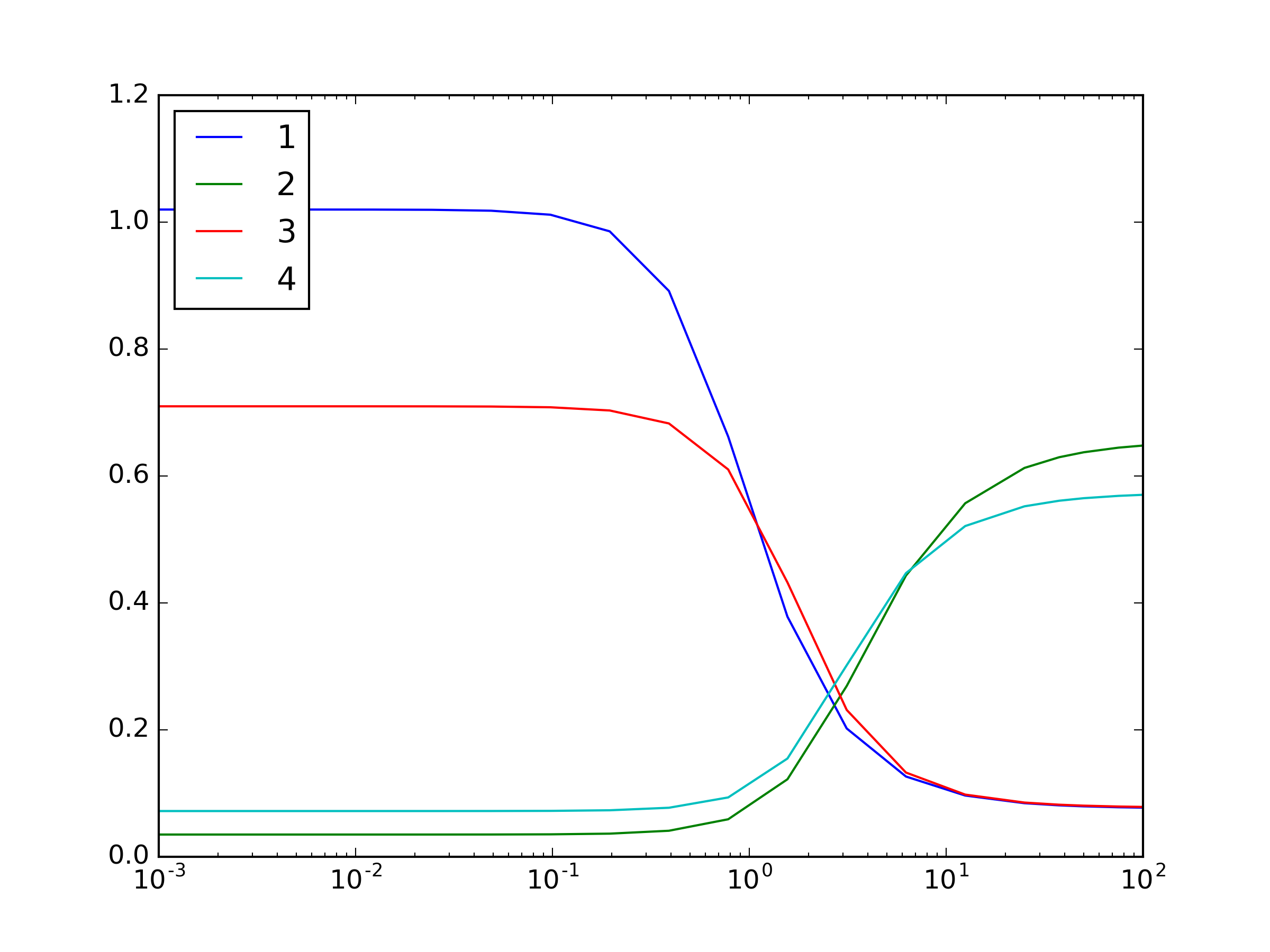

Supplement: Supplementary Software 1 — R cytometry data processing scripts and mathematical modeling scripts [file ncomms15459-s3.zip › Supplementary Software 1/FittingScripts/Results/Output/FittingScript_DoseExp1_20160330.py_model_image_2016-04-03-08-02-19_1459695739186334.png]

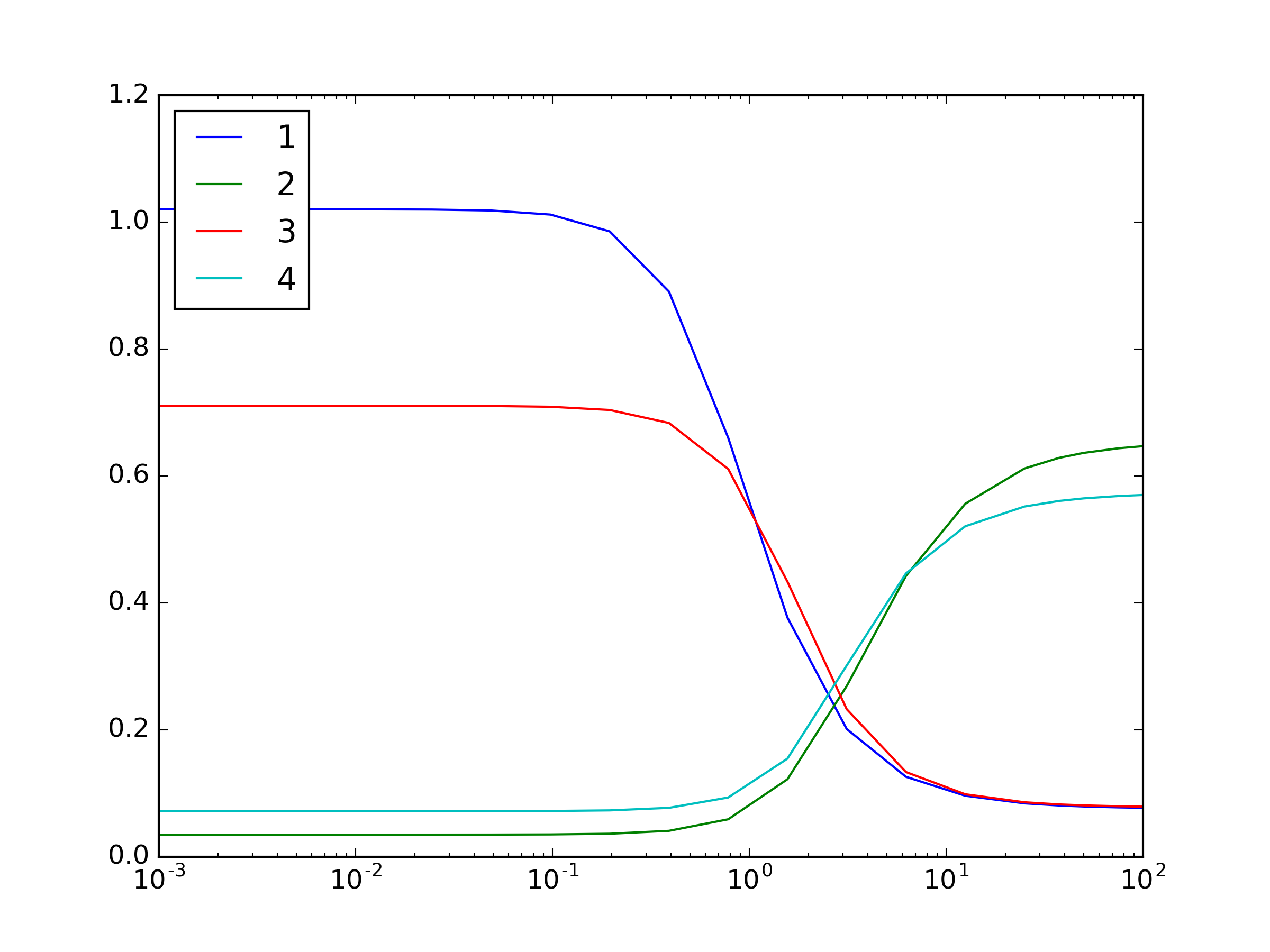

Supplement: Supplementary Software 1 — R cytometry data processing scripts and mathematical modeling scripts [file ncomms15459-s3.zip › Supplementary Software 1/FittingScripts/Results/Output/FittingScript_DoseExp1_20160330.py_model_image_2016-04-03-10-36-00_1459704960245009.png]

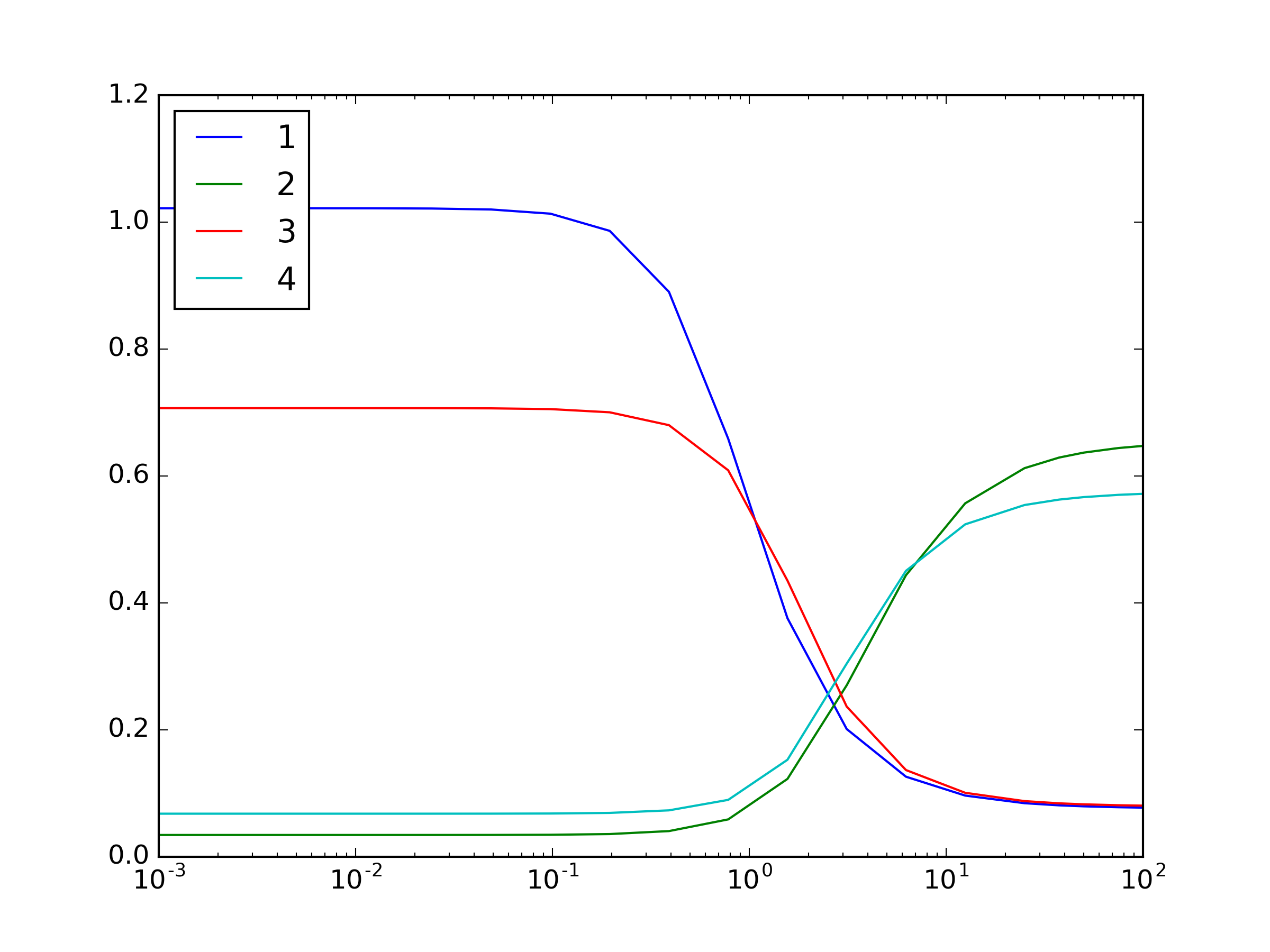

Supplement: Supplementary Software 1 — R cytometry data processing scripts and mathematical modeling scripts [file ncomms15459-s3.zip › Supplementary Software 1/FittingScripts/Results/Output/FittingScript_DoseExp1_20160330.py_model_image_2016-04-03-14-56-43_1459720603727201.png]

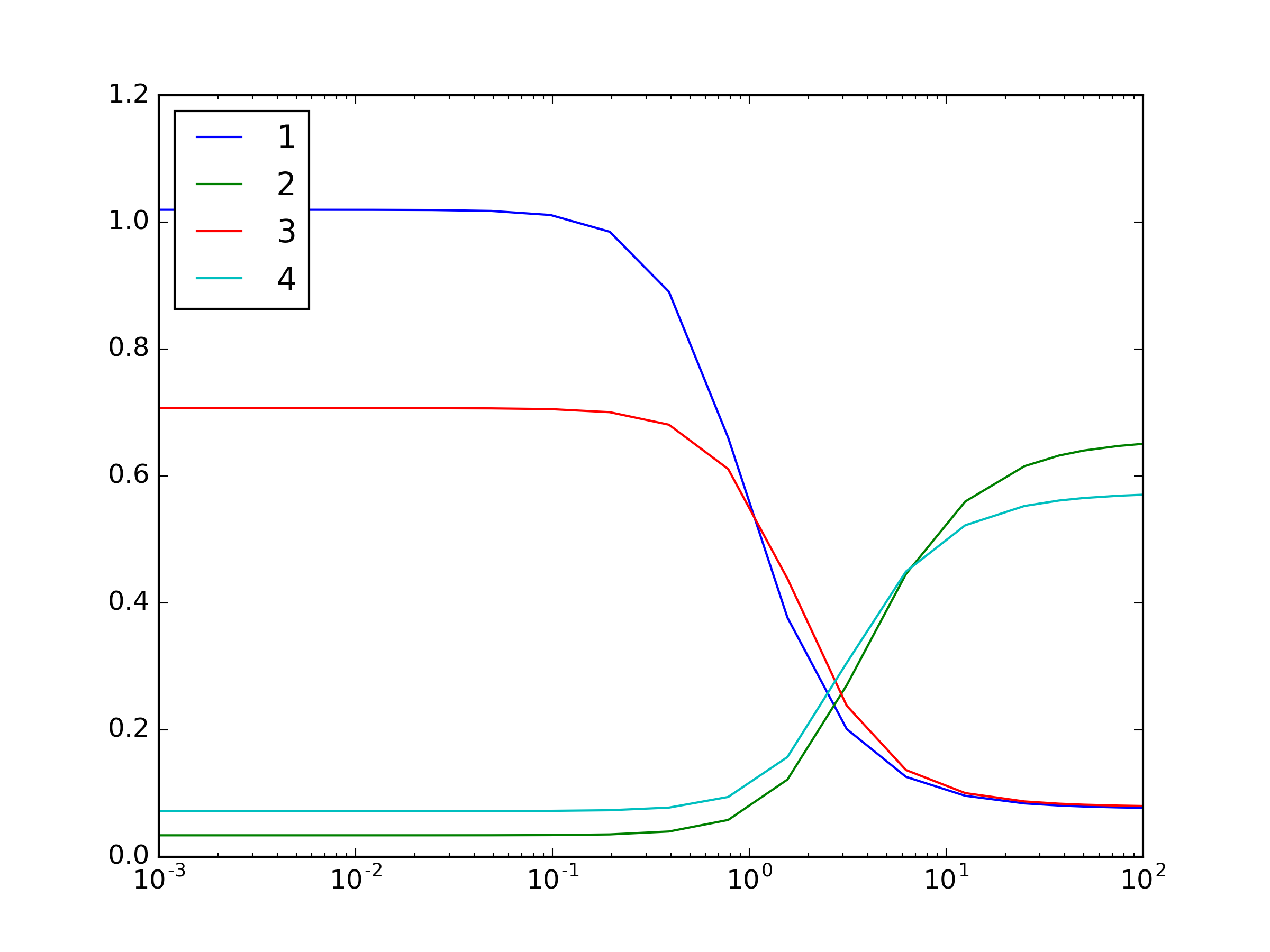

Supplement: Supplementary Software 1 — R cytometry data processing scripts and mathematical modeling scripts [file ncomms15459-s3.zip › Supplementary Software 1/FittingScripts/Results/Output/FittingScript_DoseExp1_20160330.py_model_image_2016-04-03-17-24-58_1459729498175931.png]

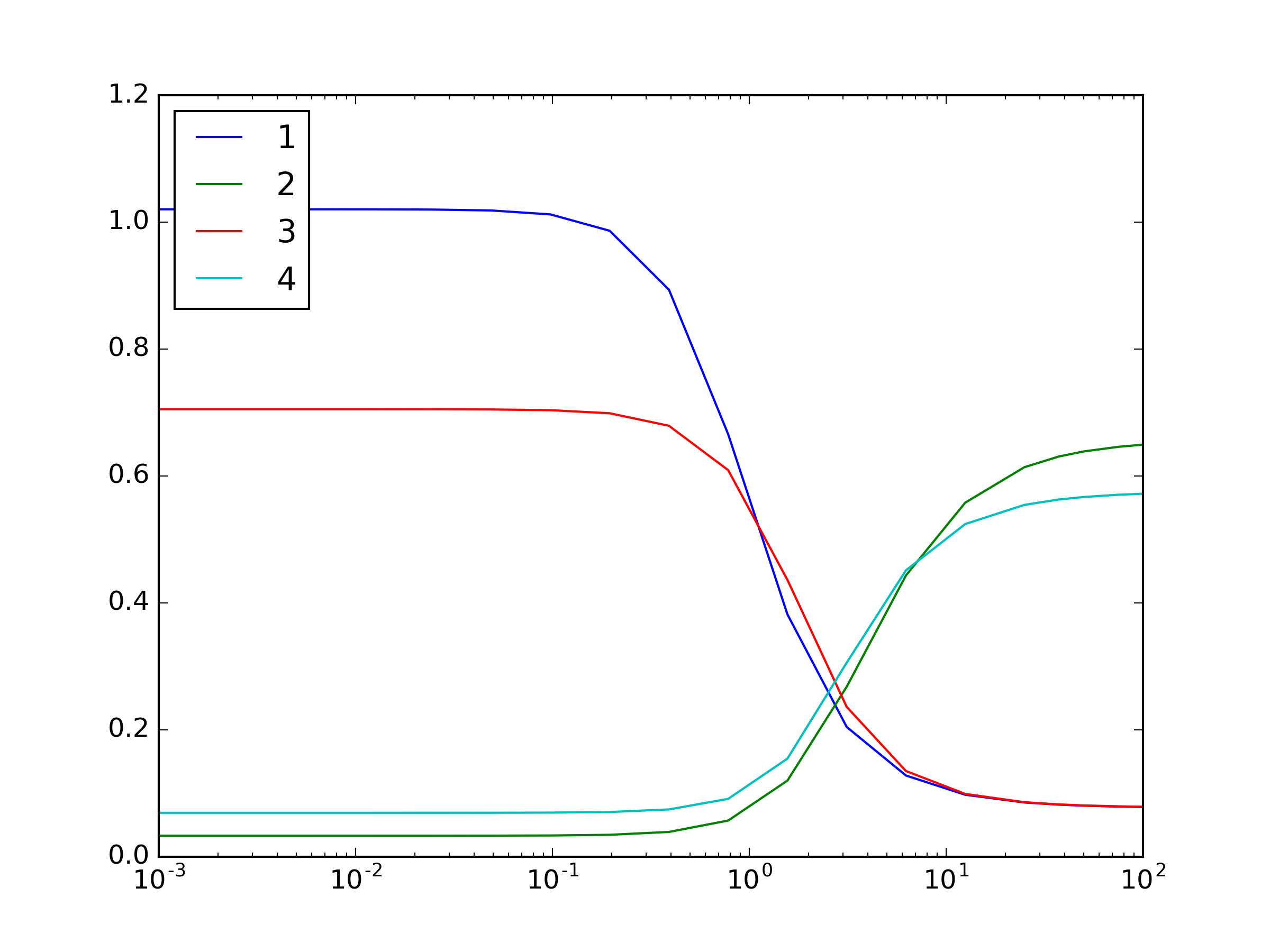

Supplement: Supplementary Software 1 — R cytometry data processing scripts and mathematical modeling scripts [file ncomms15459-s3.zip › Supplementary Software 1/FittingScripts/Results/Output/FittingScript_DoseExp1_20160330.py_model_image_2016-04-03-20-12-09_1459739529623110.png]

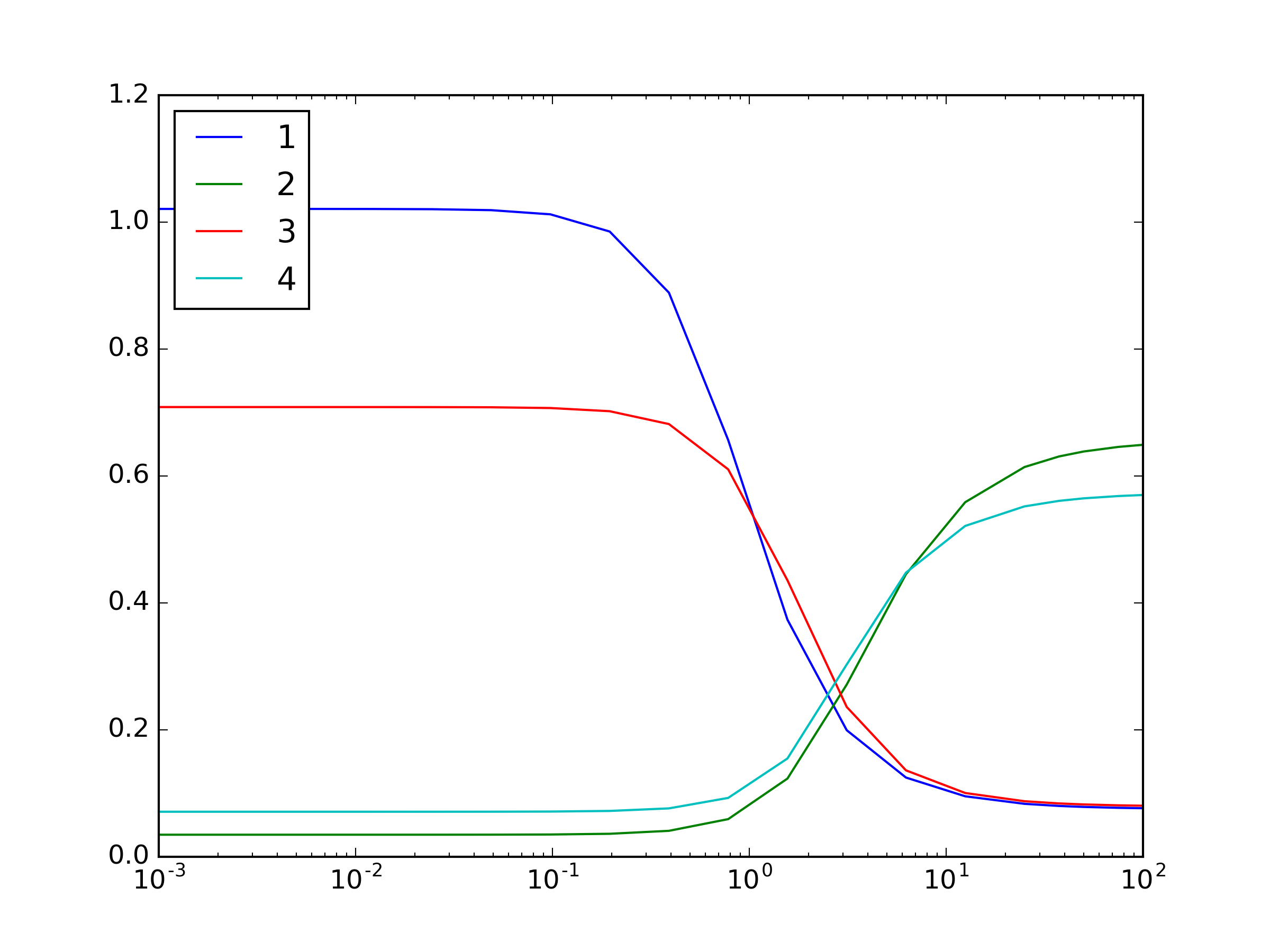

Supplement: Supplementary Software 1 — R cytometry data processing scripts and mathematical modeling scripts [file ncomms15459-s3.zip › Supplementary Software 1/FittingScripts/Results/Output/FittingScript_DoseExp1_20160330.py_model_image_2016-04-03-22-52-50_1459749170938957.png]

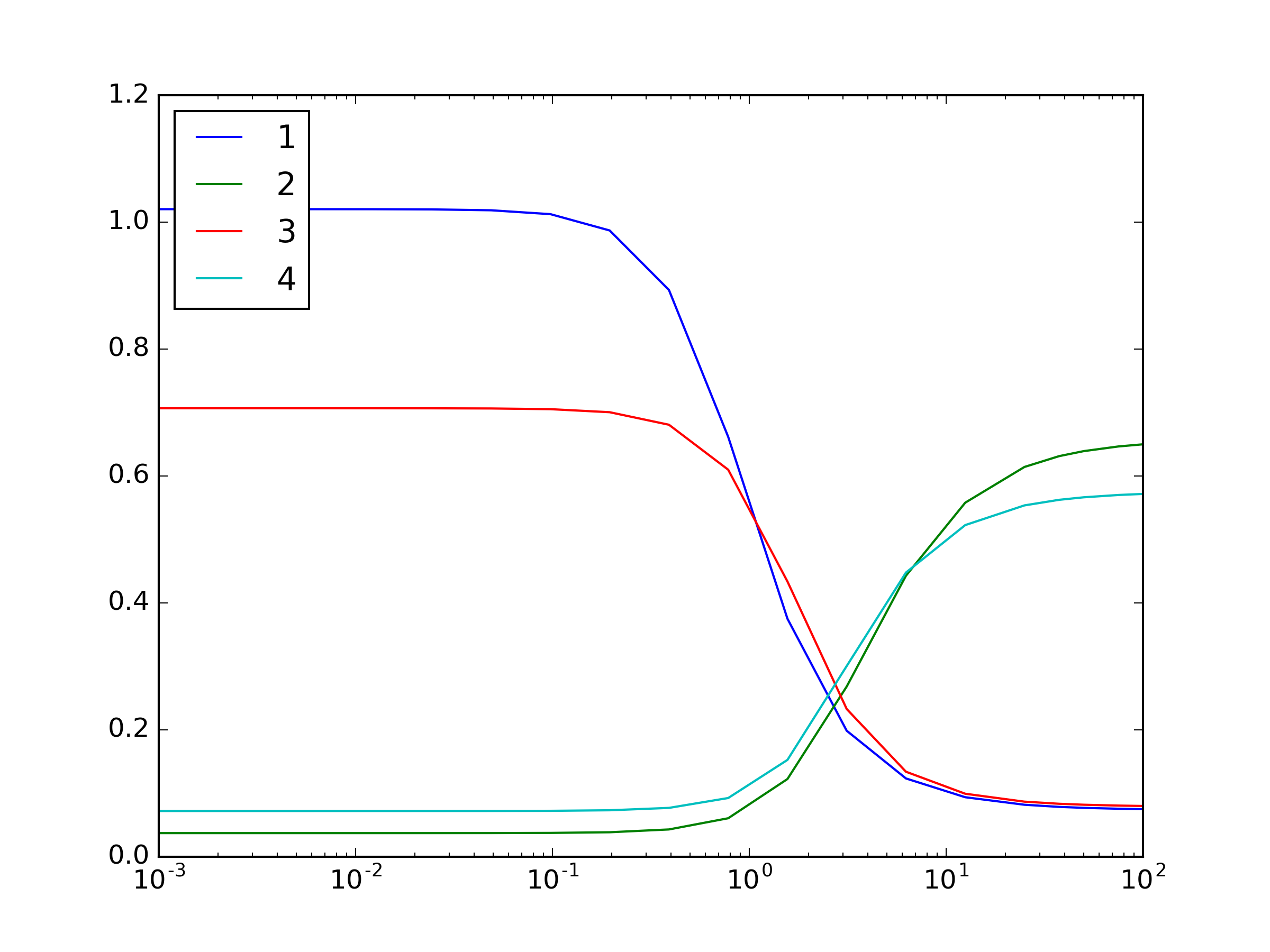

Supplement: Supplementary Software 1 — R cytometry data processing scripts and mathematical modeling scripts [file ncomms15459-s3.zip › Supplementary Software 1/FittingScripts/Results/Output/FittingScript_DoseExp1_20160330.py_model_image_2016-04-04-00-55-23_1459756523367027.png]

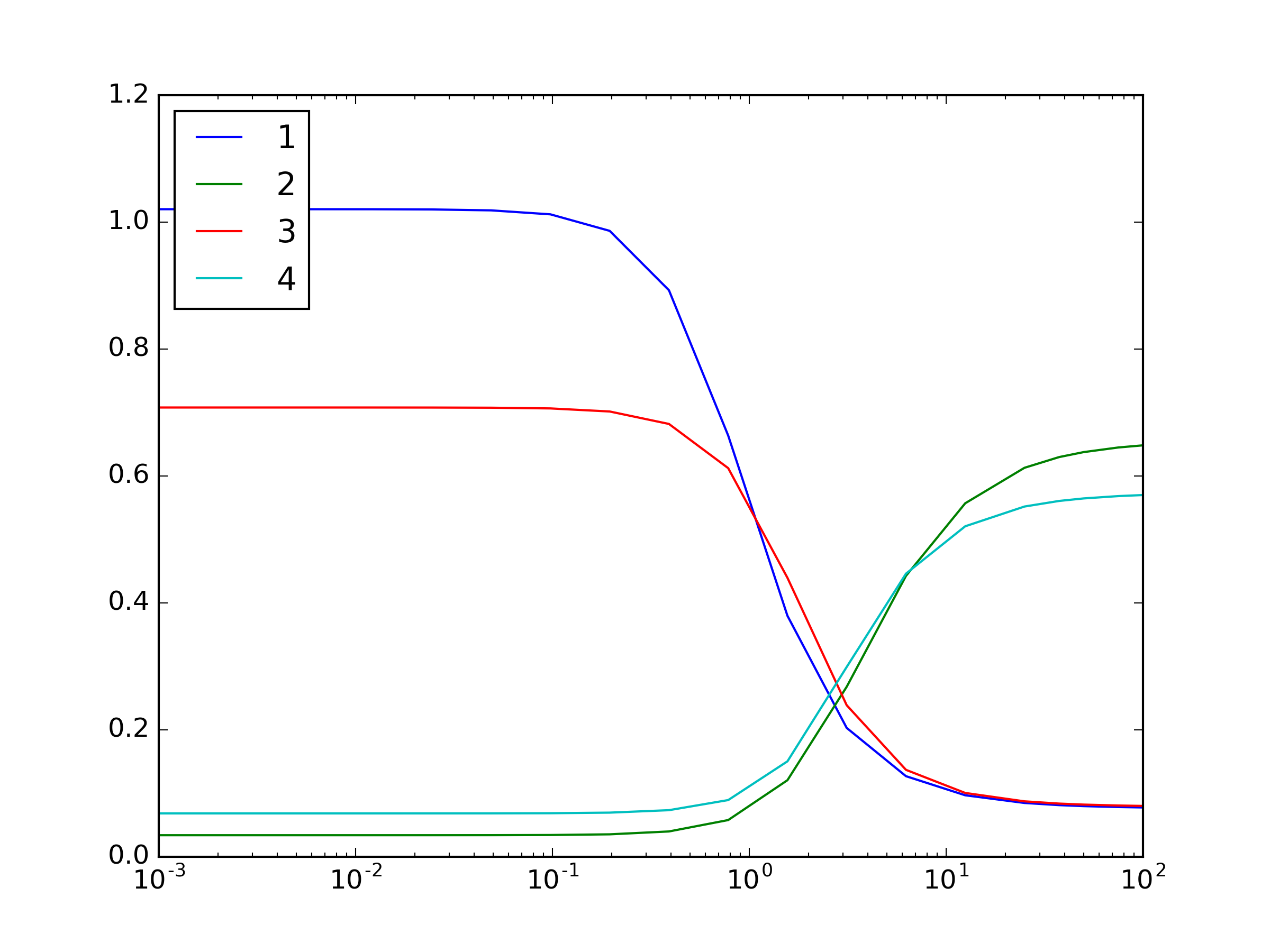

Supplement: Supplementary Software 1 — R cytometry data processing scripts and mathematical modeling scripts [file ncomms15459-s3.zip › Supplementary Software 1/FittingScripts/Results/Output/FittingScript_DoseExp1_20160330.py_model_image_2016-04-04-03-26-48_1459765608124732.png]

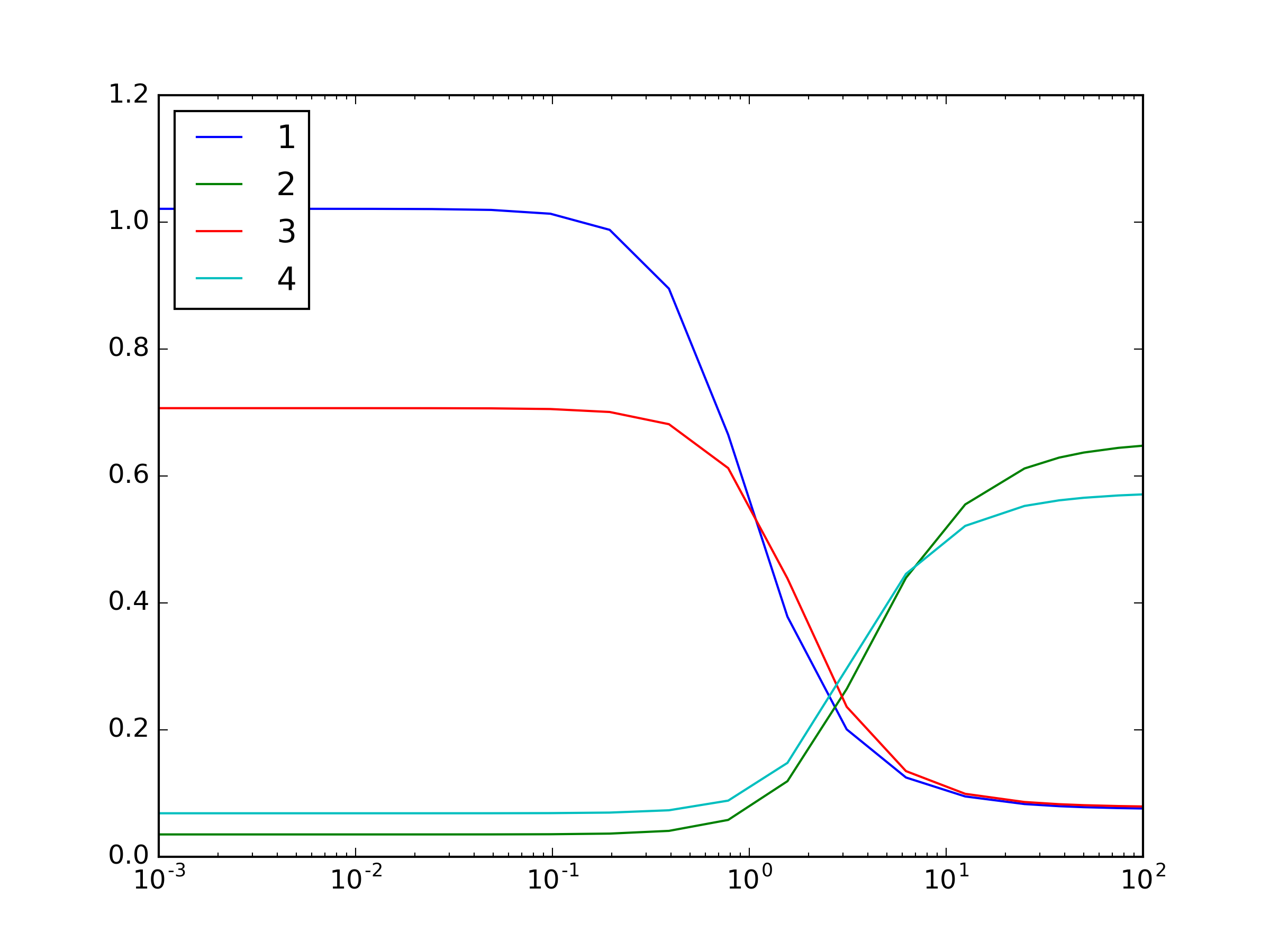

Supplement: Supplementary Software 1 — R cytometry data processing scripts and mathematical modeling scripts [file ncomms15459-s3.zip › Supplementary Software 1/FittingScripts/Results/Output/FittingScript_DoseExp1_20160330.py_model_image_2016-04-04-06-40-17_1459777217468674.png]

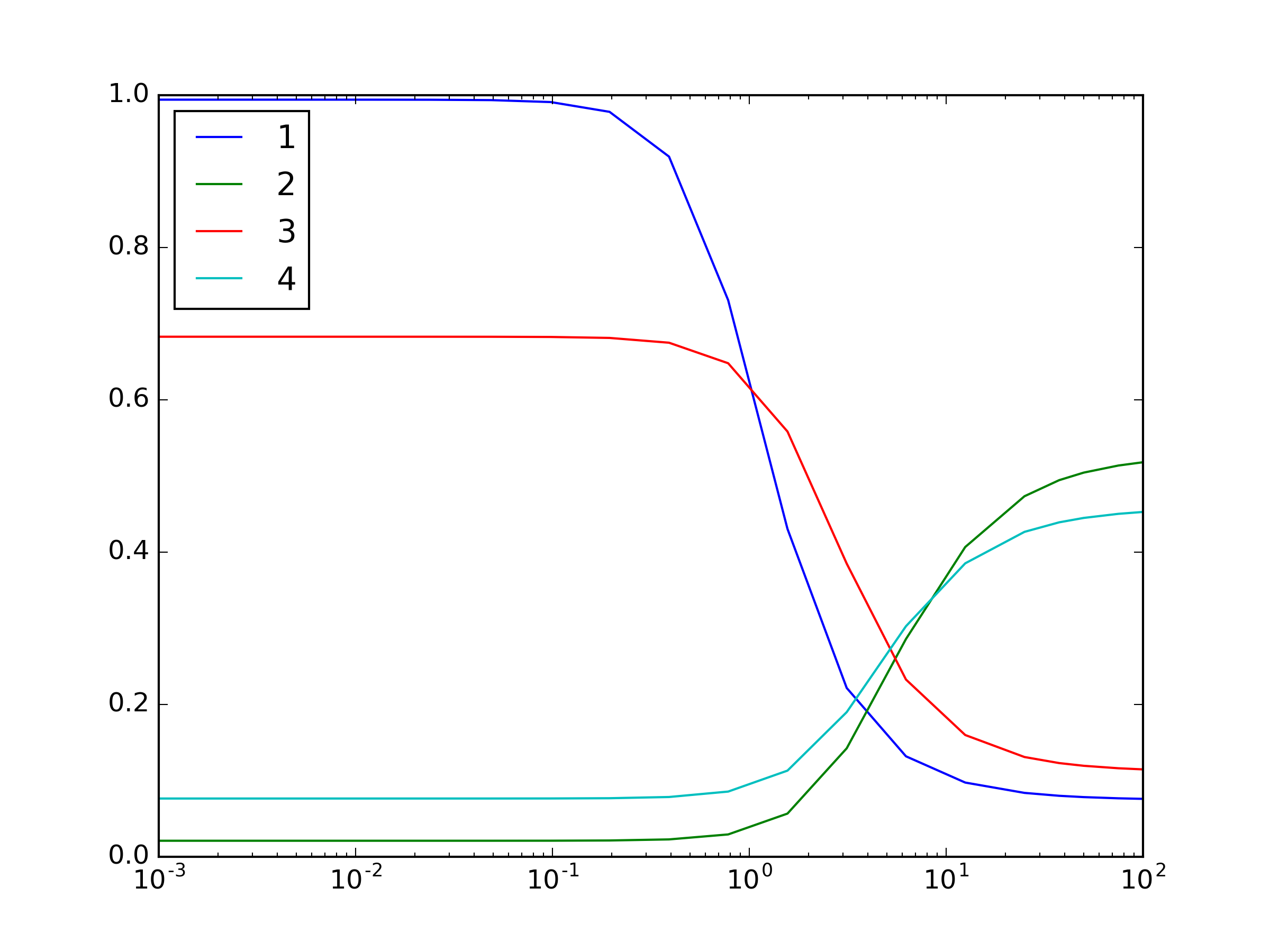

Supplement: Supplementary Software 1 — R cytometry data processing scripts and mathematical modeling scripts [file ncomms15459-s3.zip › Supplementary Software 1/FittingScripts/Results/Output/FittingScript_DoseExp2_20160330.py_model_image_2016-04-01-12-26-48_1459538808647907.png]

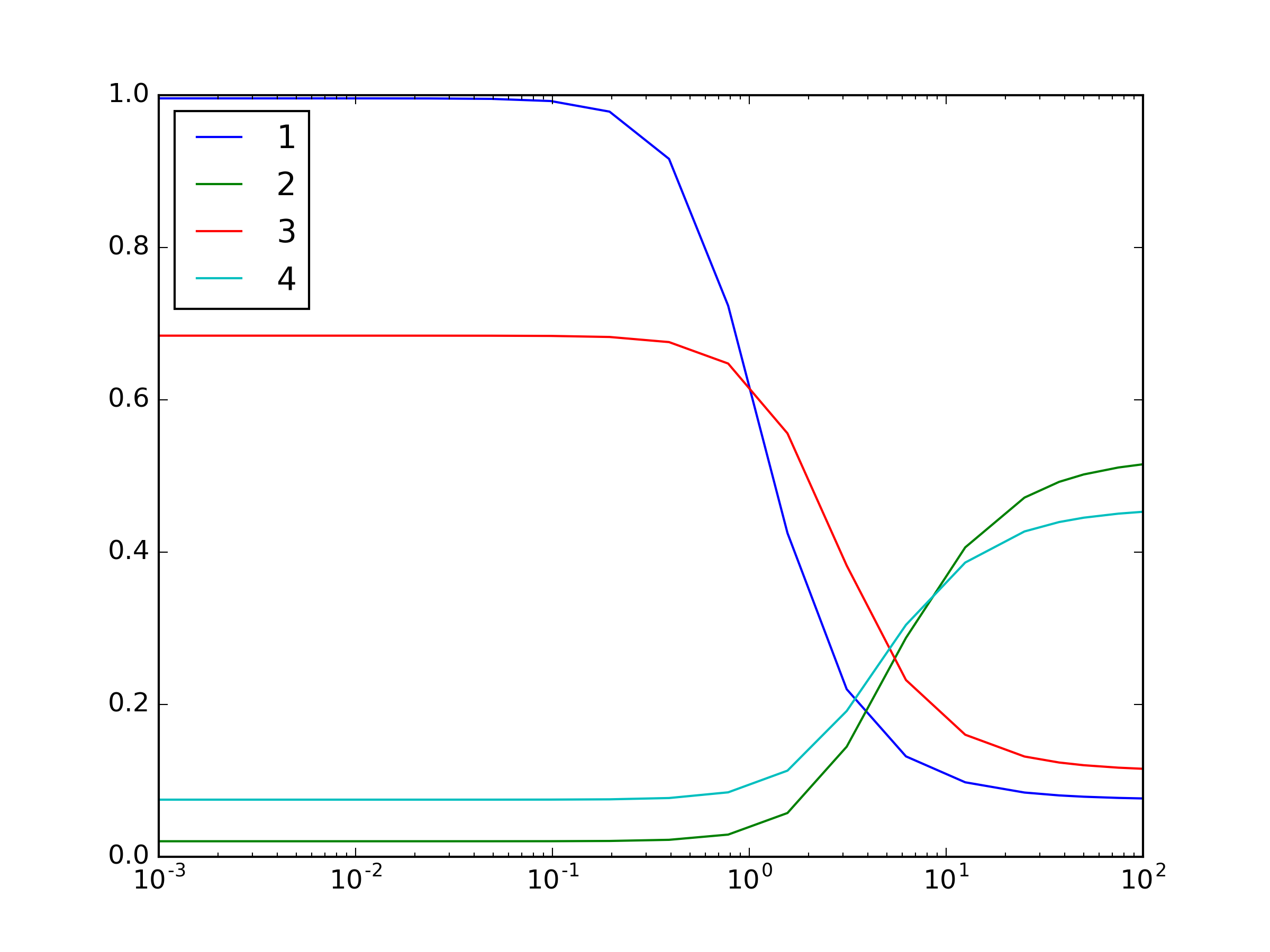

Supplement: Supplementary Software 1 — R cytometry data processing scripts and mathematical modeling scripts [file ncomms15459-s3.zip › Supplementary Software 1/FittingScripts/Results/Output/FittingScript_DoseExp2_20160330.py_model_image_2016-04-01-17-12-58_1459555978528267.png]

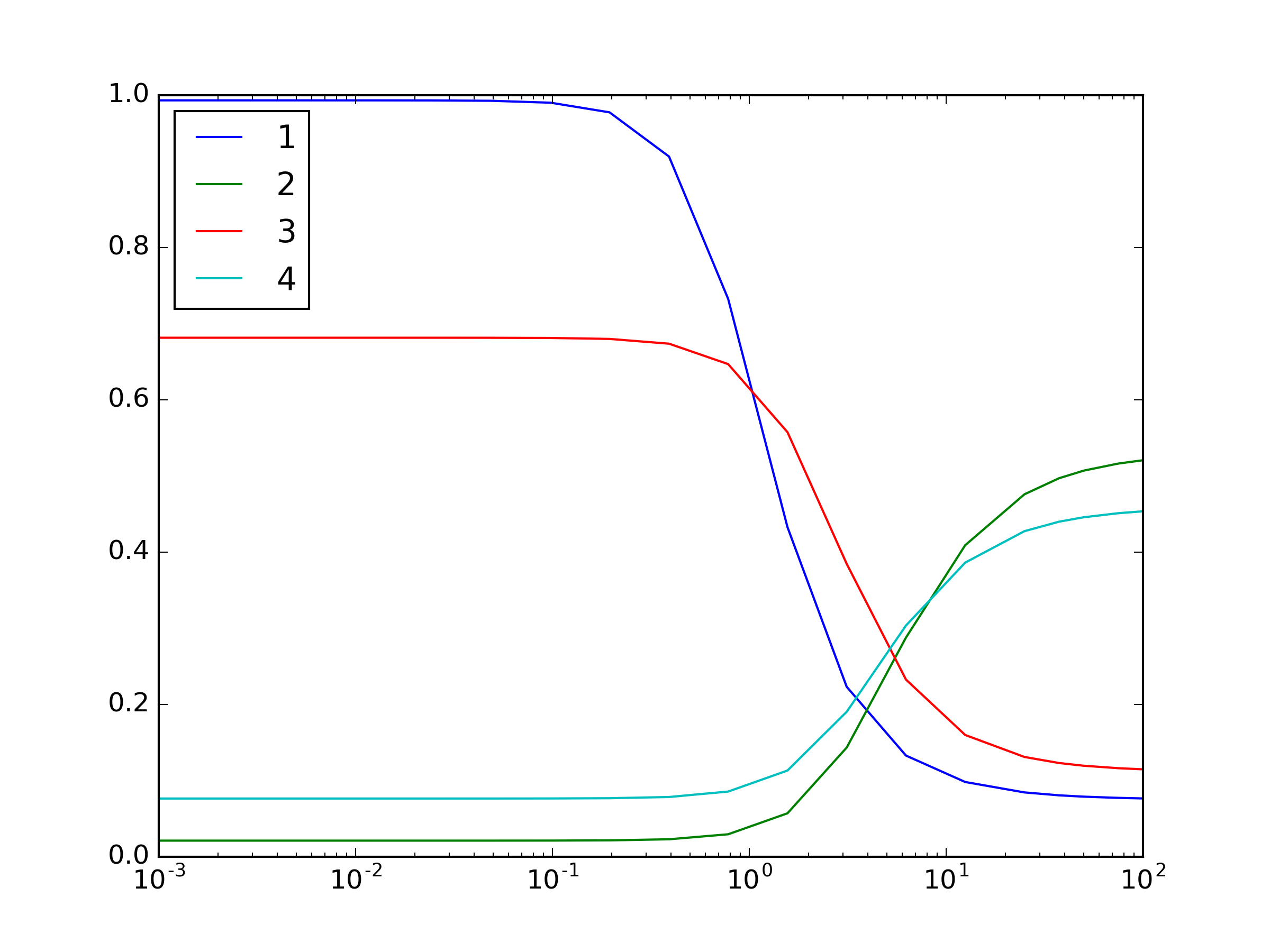

Supplement: Supplementary Software 1 — R cytometry data processing scripts and mathematical modeling scripts [file ncomms15459-s3.zip › Supplementary Software 1/FittingScripts/Results/Output/FittingScript_DoseExp2_20160330.py_model_image_2016-04-01-21-53-03_1459572783123379.png]

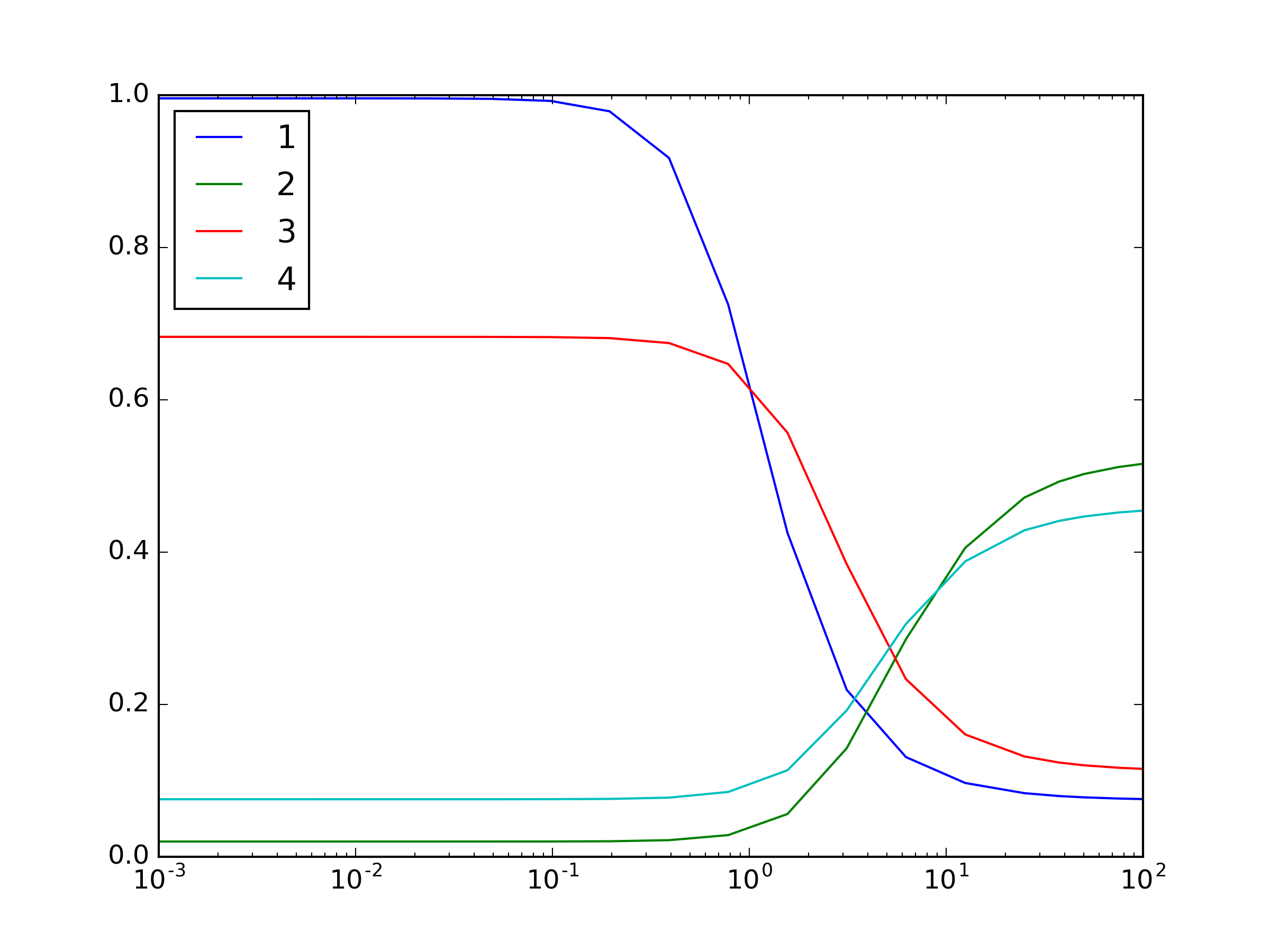

Supplement: Supplementary Software 1 — R cytometry data processing scripts and mathematical modeling scripts [file ncomms15459-s3.zip › Supplementary Software 1/FittingScripts/Results/Output/FittingScript_DoseExp2_20160330.py_model_image_2016-04-02-00-51-12_1459583472139206.png]

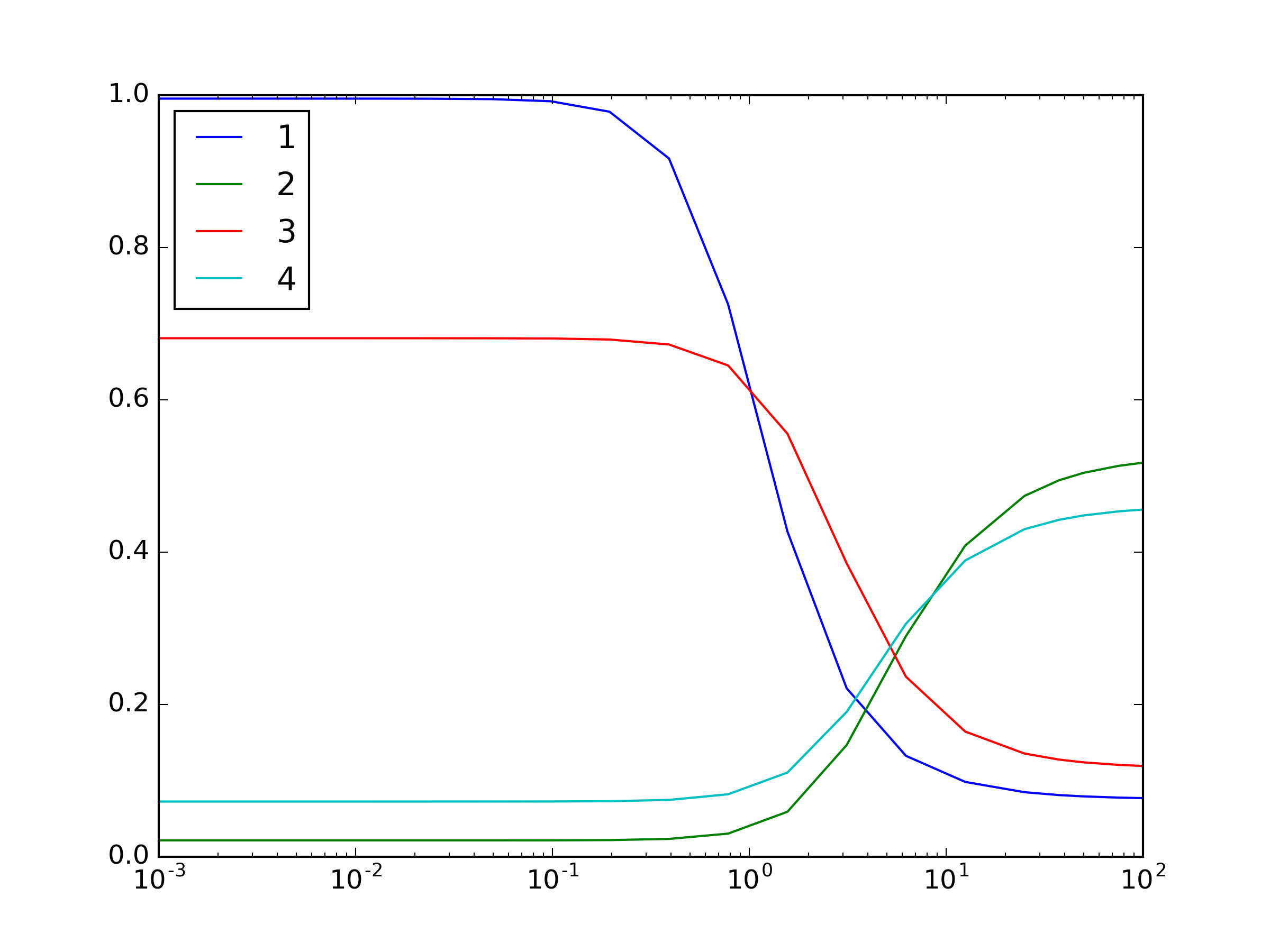

Supplement: Supplementary Software 1 — R cytometry data processing scripts and mathematical modeling scripts [file ncomms15459-s3.zip › Supplementary Software 1/FittingScripts/Results/Output/FittingScript_DoseExp2_20160330.py_model_image_2016-04-02-03-50-08_1459594208486466.png]

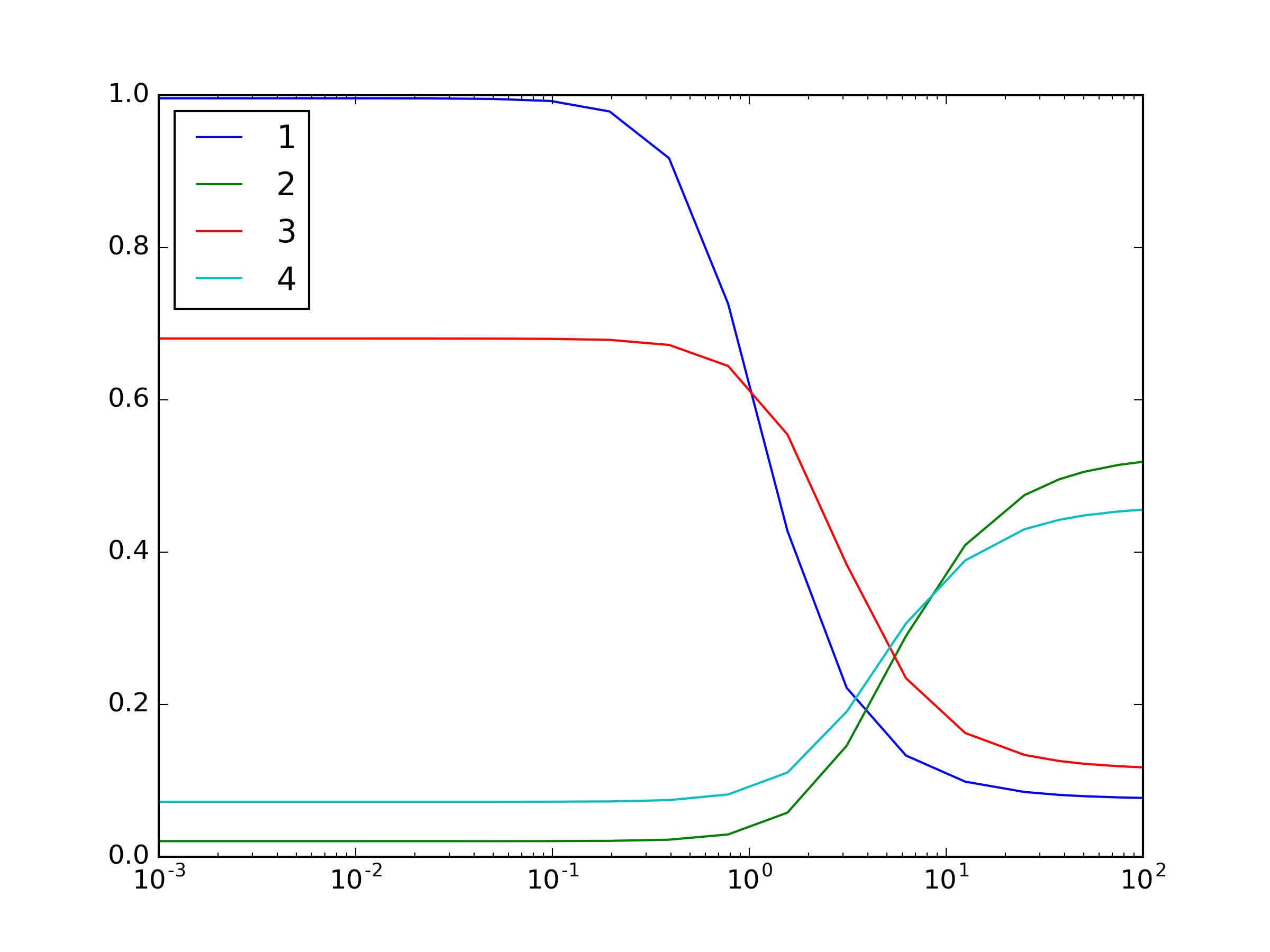

Supplement: Supplementary Software 1 — R cytometry data processing scripts and mathematical modeling scripts [file ncomms15459-s3.zip › Supplementary Software 1/FittingScripts/Results/Output/FittingScript_DoseExp2_20160330.py_model_image_2016-04-02-07-04-17_1459605857570675.png]

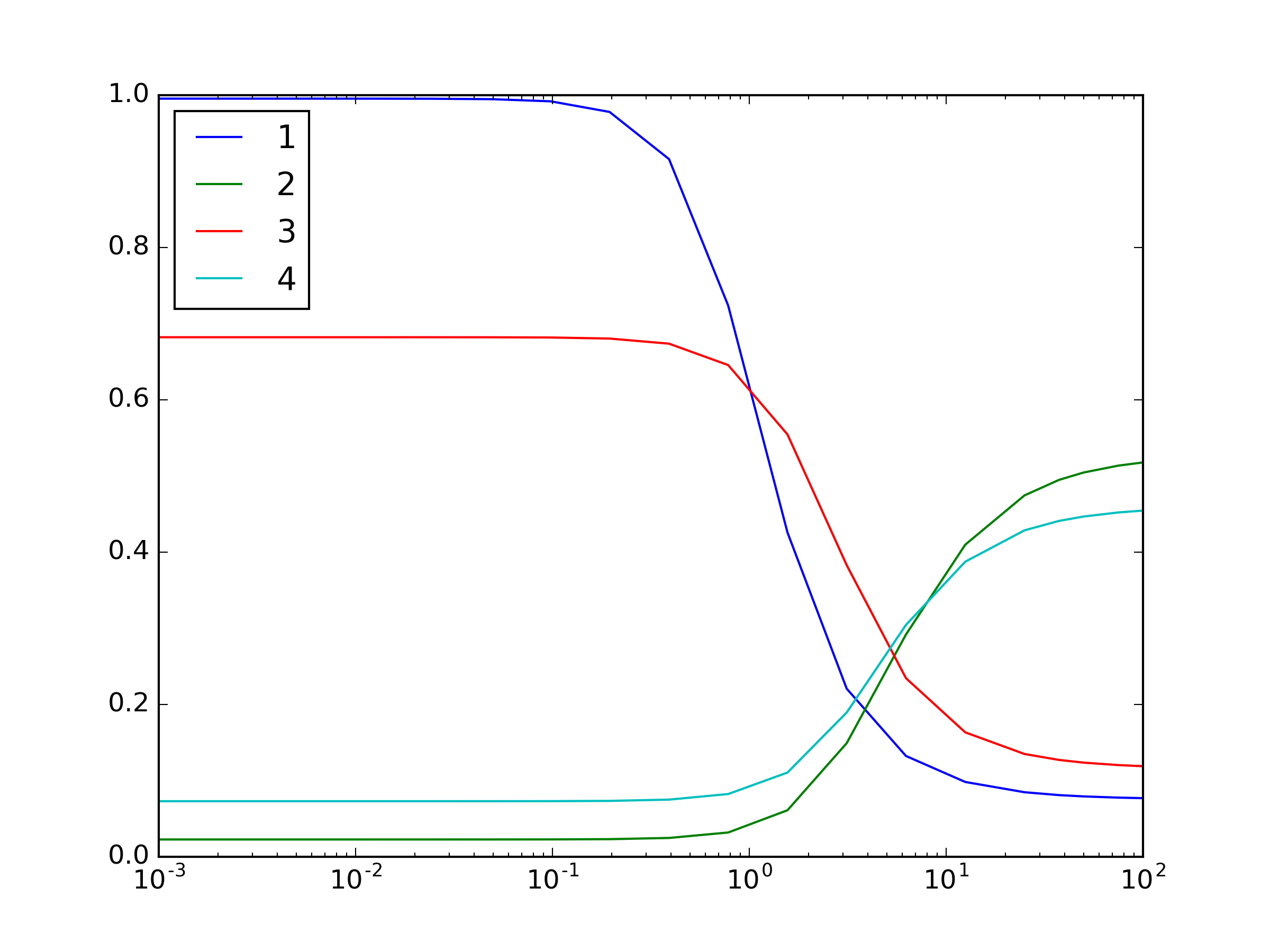

Supplement: Supplementary Software 1 — R cytometry data processing scripts and mathematical modeling scripts [file ncomms15459-s3.zip › Supplementary Software 1/FittingScripts/Results/Output/FittingScript_DoseExp2_20160330.py_model_image_2016-04-02-11-53-42_1459623222618295.png]

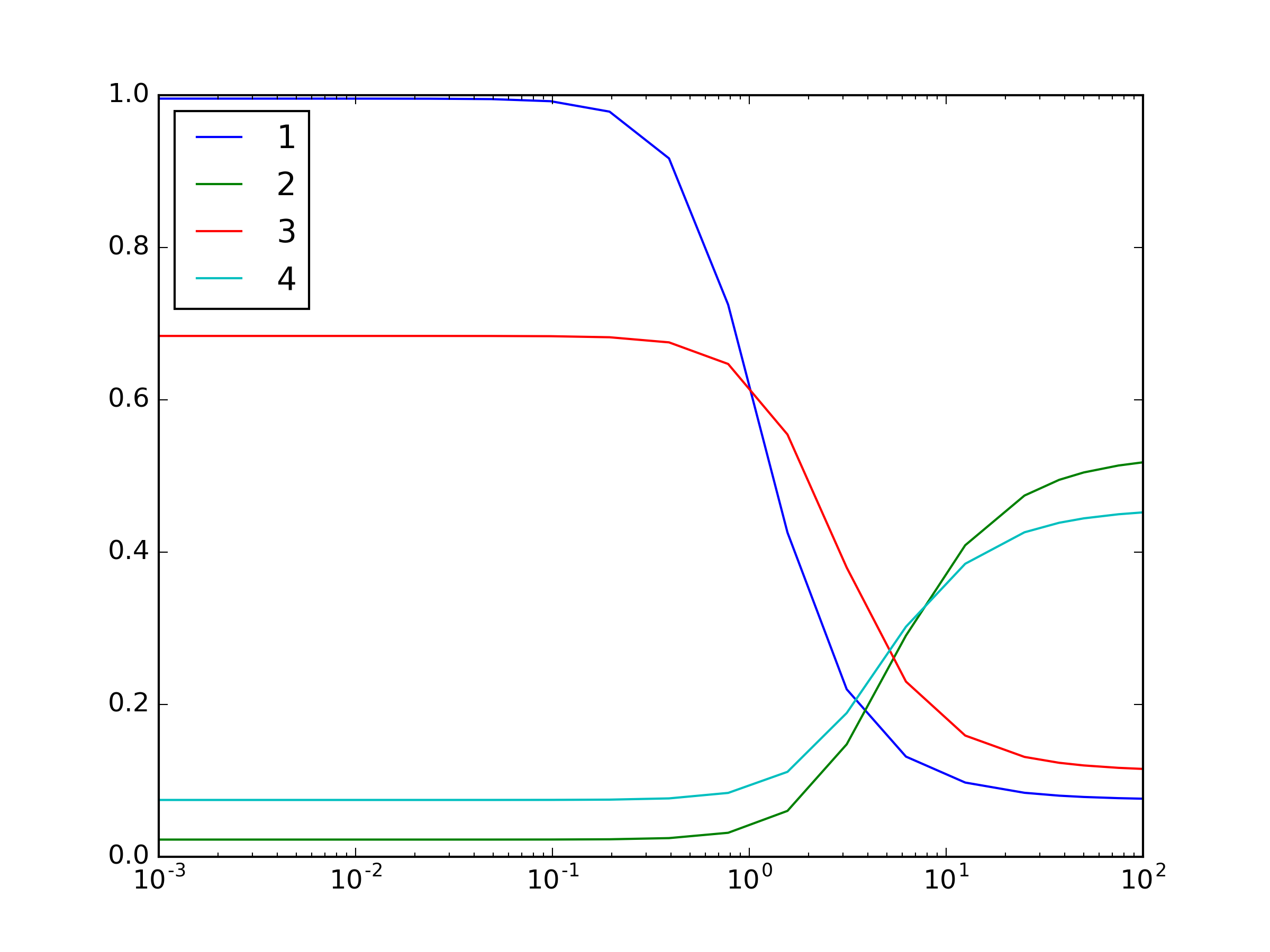

Supplement: Supplementary Software 1 — R cytometry data processing scripts and mathematical modeling scripts [file ncomms15459-s3.zip › Supplementary Software 1/FittingScripts/Results/Output/FittingScript_DoseExp2_20160330.py_model_image_2016-04-02-15-01-39_1459634499752606.png]

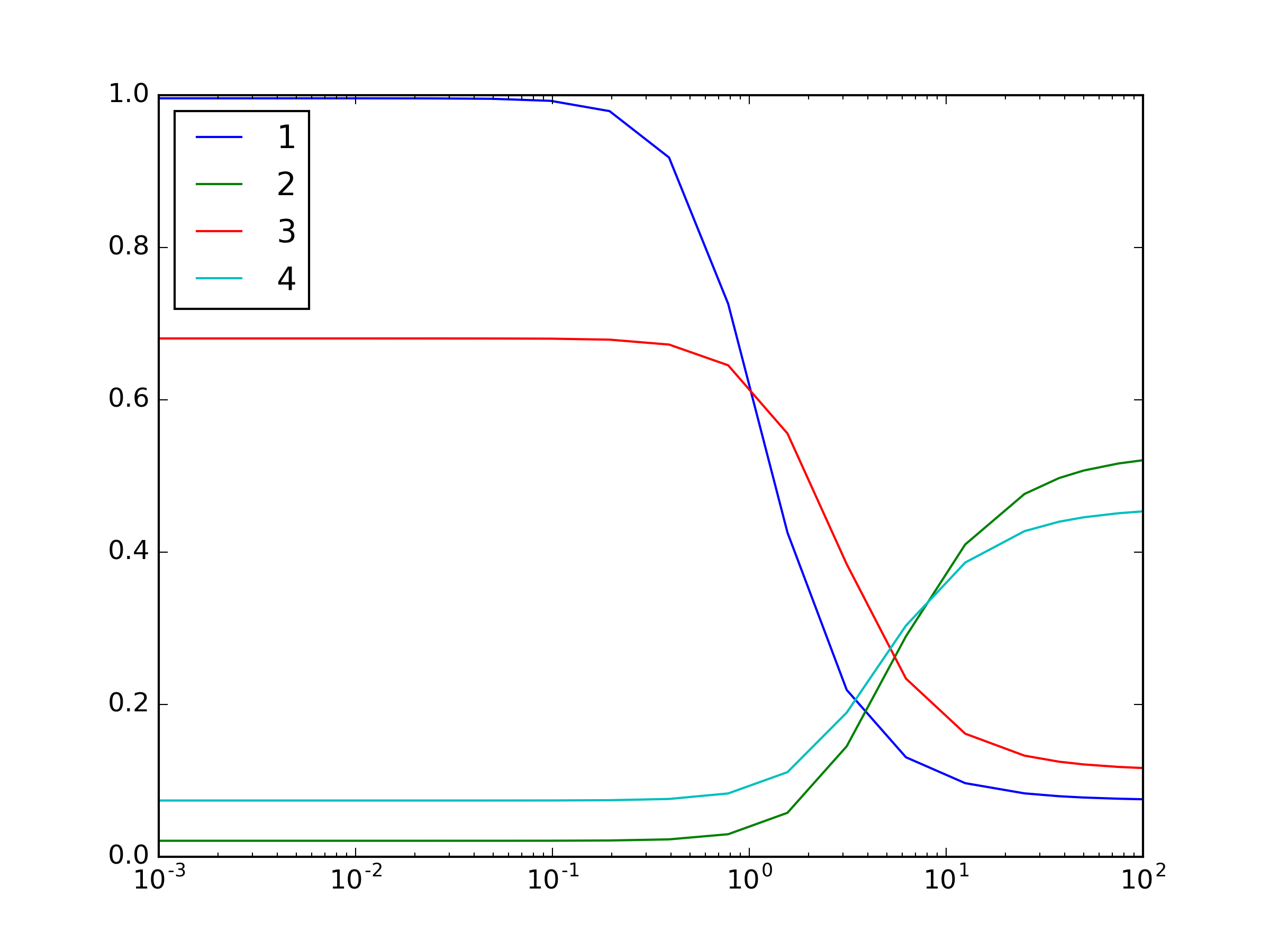

Supplement: Supplementary Software 1 — R cytometry data processing scripts and mathematical modeling scripts [file ncomms15459-s3.zip › Supplementary Software 1/FittingScripts/Results/Output/FittingScript_DoseExp2_20160330.py_model_image_2016-04-02-18-58-40_1459648720451950.png]

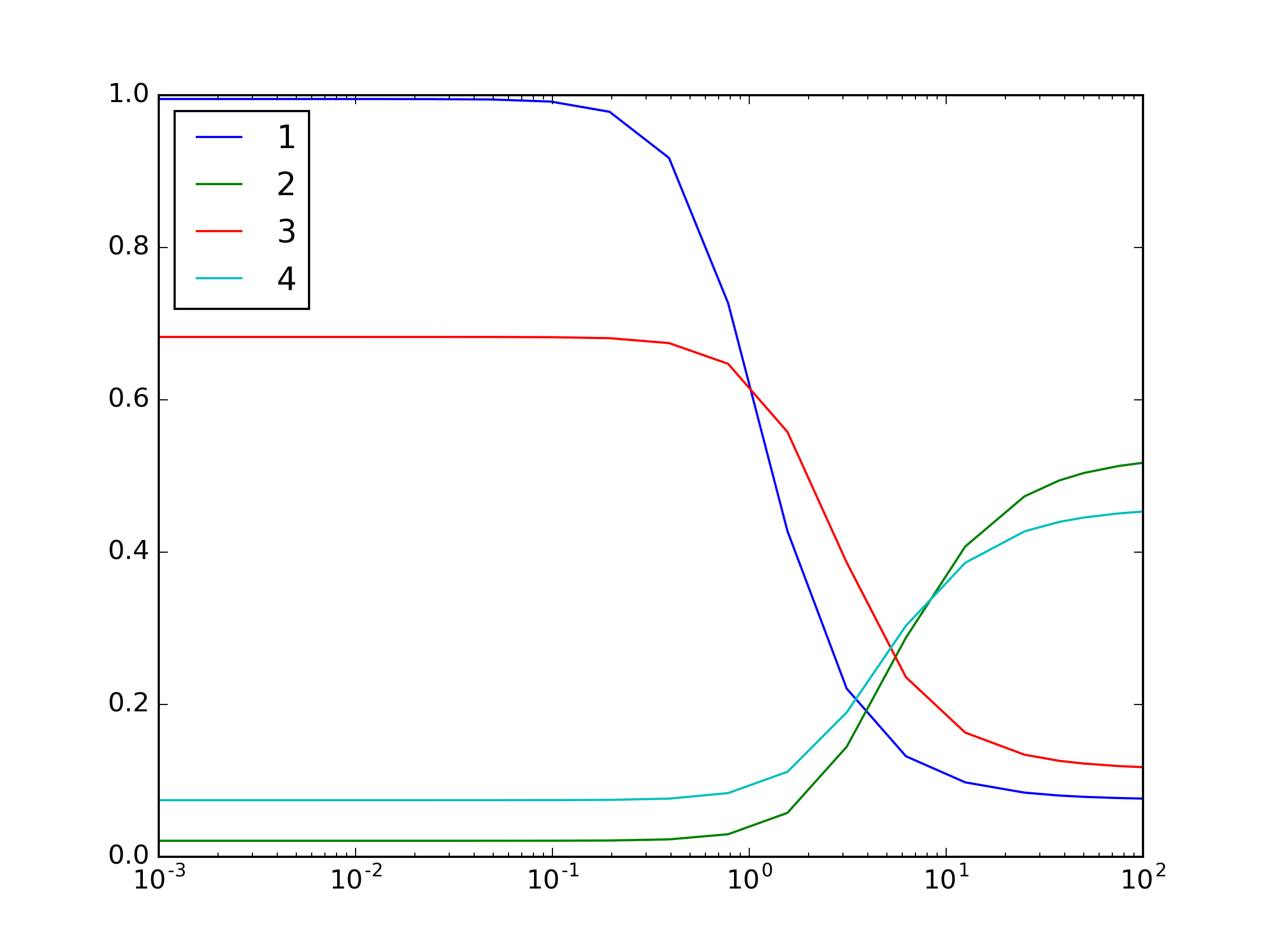

Supplement: Supplementary Software 1 — R cytometry data processing scripts and mathematical modeling scripts [file ncomms15459-s3.zip › Supplementary Software 1/FittingScripts/Results/Output/FittingScript_DoseExp2_20160330.py_model_image_2016-04-02-21-56-08_1459659368244087.png]

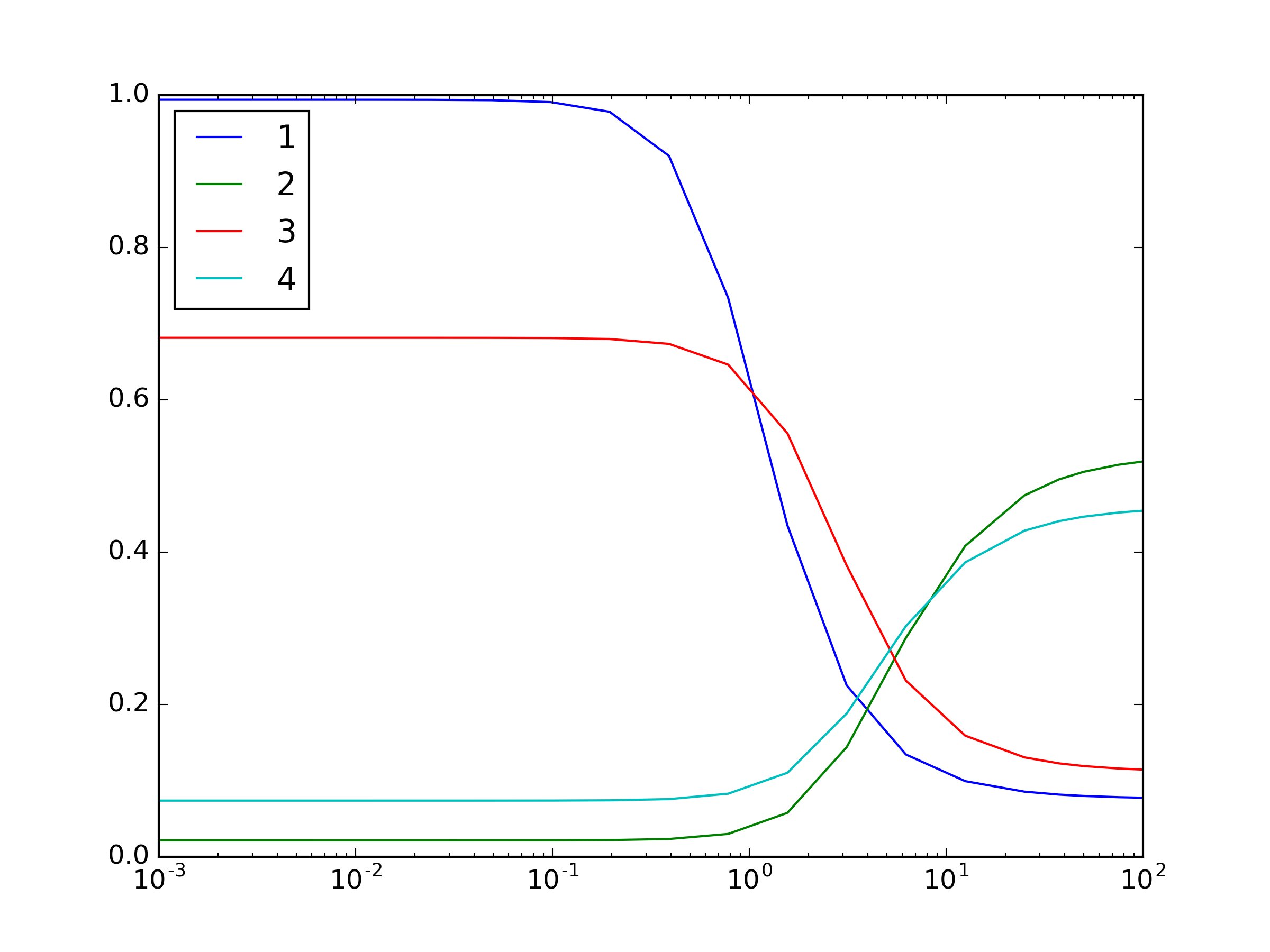

Supplement: Supplementary Software 1 — R cytometry data processing scripts and mathematical modeling scripts [file ncomms15459-s3.zip › Supplementary Software 1/FittingScripts/Results/Output/FittingScript_DoseExp2_20160330.py_model_image_2016-04-03-01-53-36_1459673616316402.png]

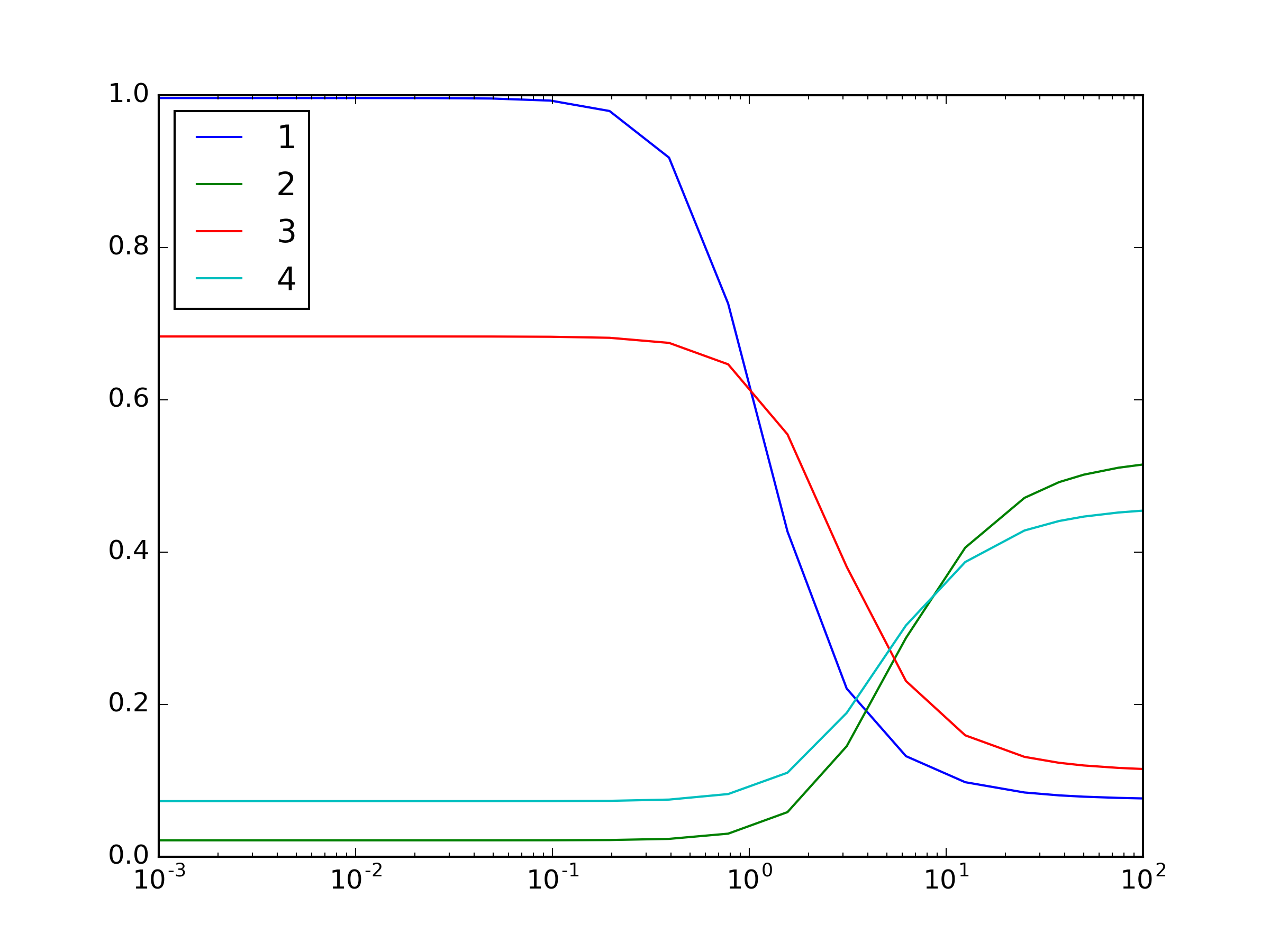

Supplement: Supplementary Software 1 — R cytometry data processing scripts and mathematical modeling scripts [file ncomms15459-s3.zip › Supplementary Software 1/FittingScripts/Results/Output/FittingScript_DoseExp2_20160330.py_model_image_2016-04-03-05-03-44_1459685024120765.png]

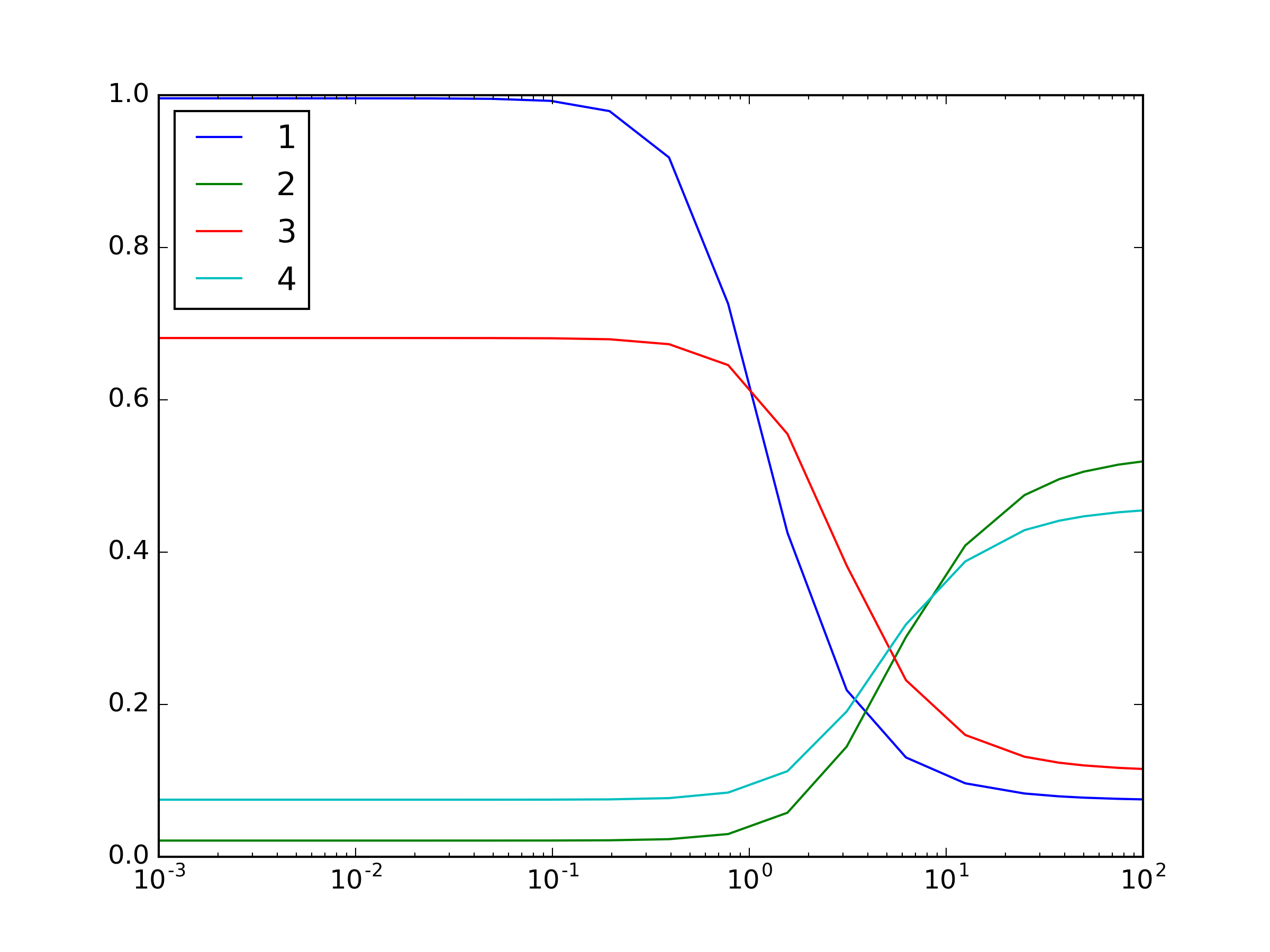

Supplement: Supplementary Software 1 — R cytometry data processing scripts and mathematical modeling scripts [file ncomms15459-s3.zip › Supplementary Software 1/FittingScripts/Results/Output/FittingScript_DoseExp2_20160330.py_model_image_2016-04-03-07-47-31_1459694851881083.png]

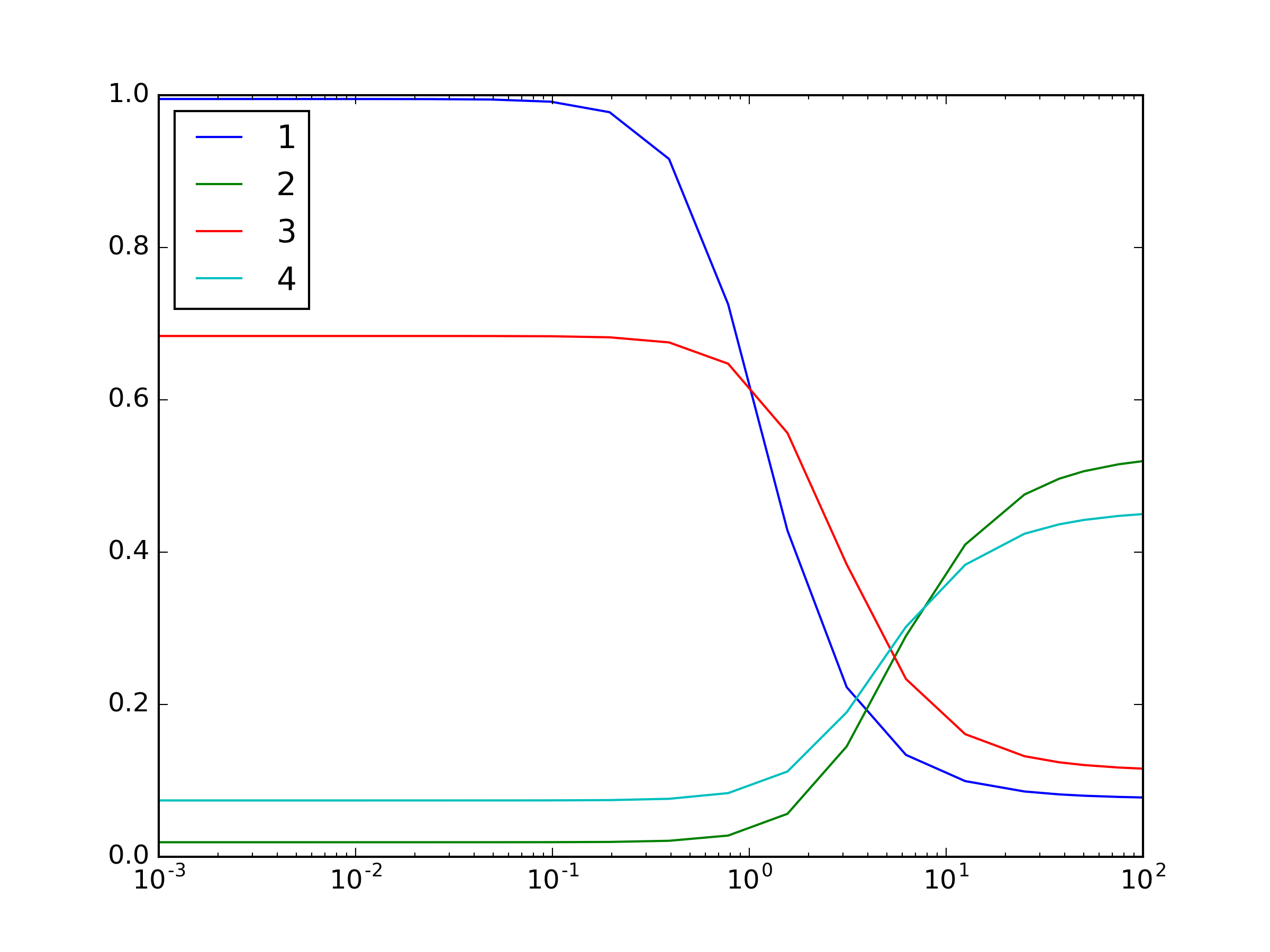

Supplement: Supplementary Software 1 — R cytometry data processing scripts and mathematical modeling scripts [file ncomms15459-s3.zip › Supplementary Software 1/FittingScripts/Results/Output/FittingScript_DoseExp2_20160330.py_model_image_2016-04-03-11-36-52_1459708612971475.png]

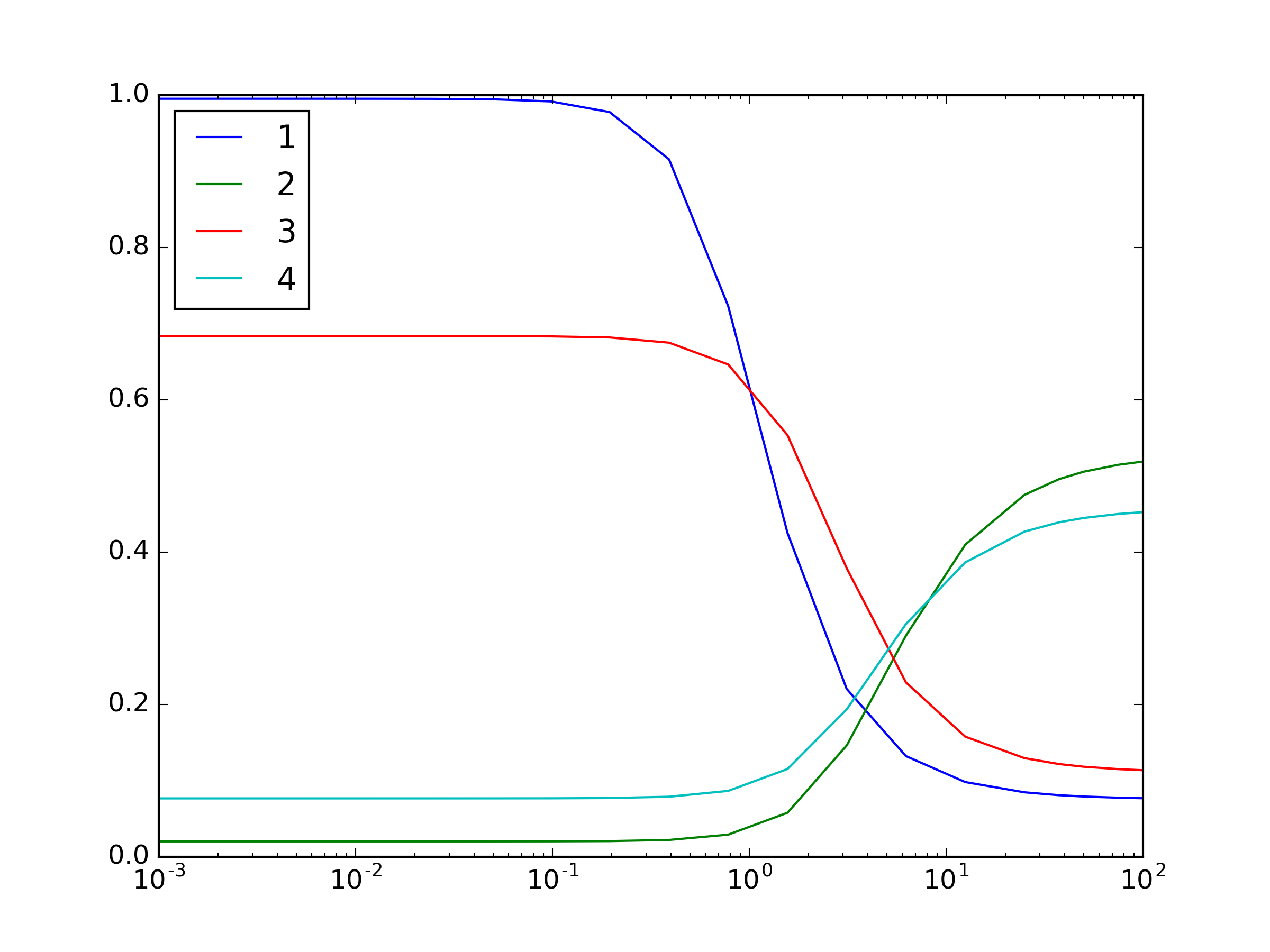

Supplement: Supplementary Software 1 — R cytometry data processing scripts and mathematical modeling scripts [file ncomms15459-s3.zip › Supplementary Software 1/FittingScripts/Results/Output/FittingScript_DoseExp2_20160330.py_model_image_2016-04-03-14-46-44_1459720004709487.png]

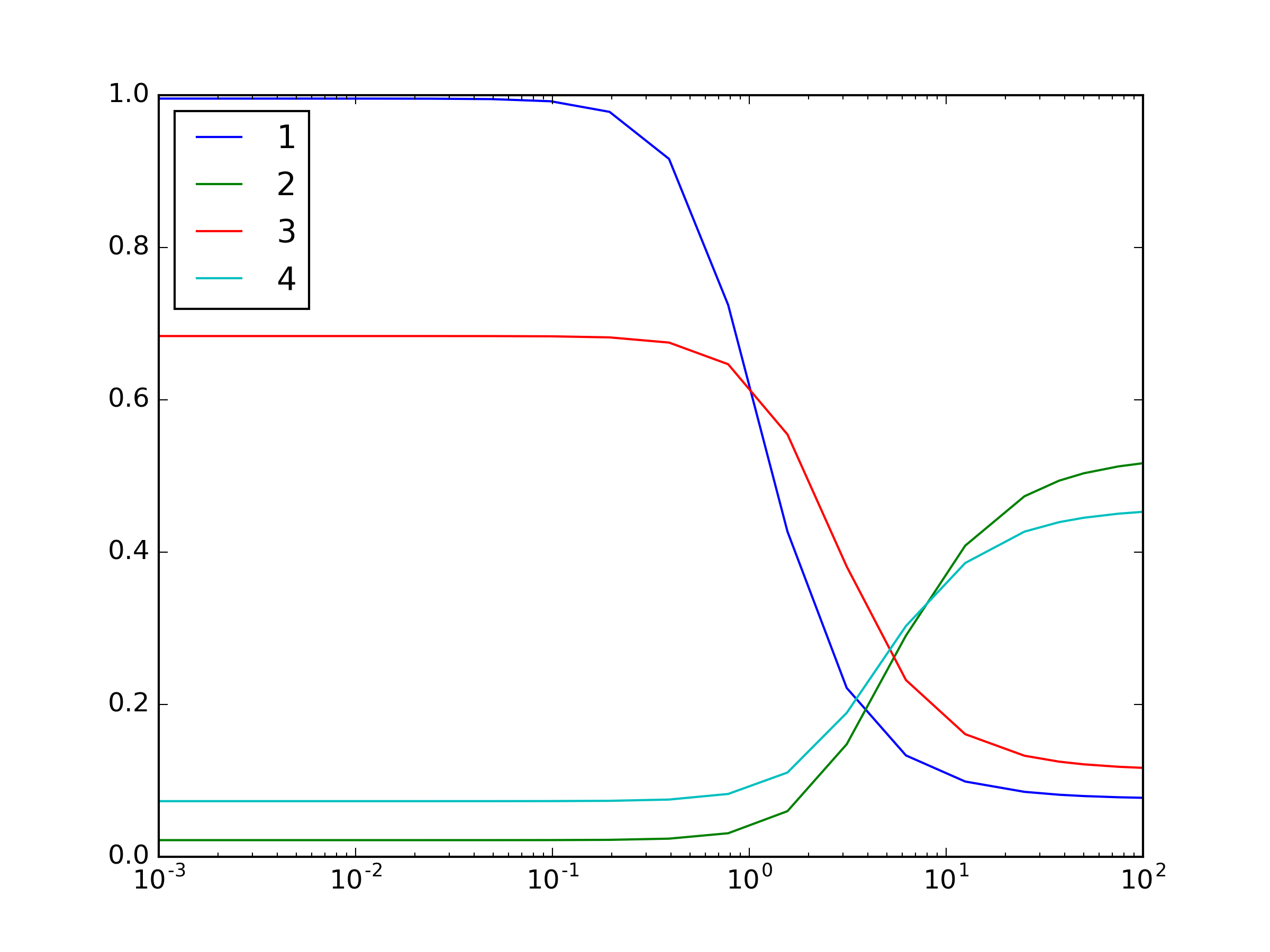

Supplement: Supplementary Software 1 — R cytometry data processing scripts and mathematical modeling scripts [file ncomms15459-s3.zip › Supplementary Software 1/FittingScripts/Results/Output/FittingScript_DoseExp2_20160330.py_model_image_2016-04-03-17-28-04_1459729684708436.png]

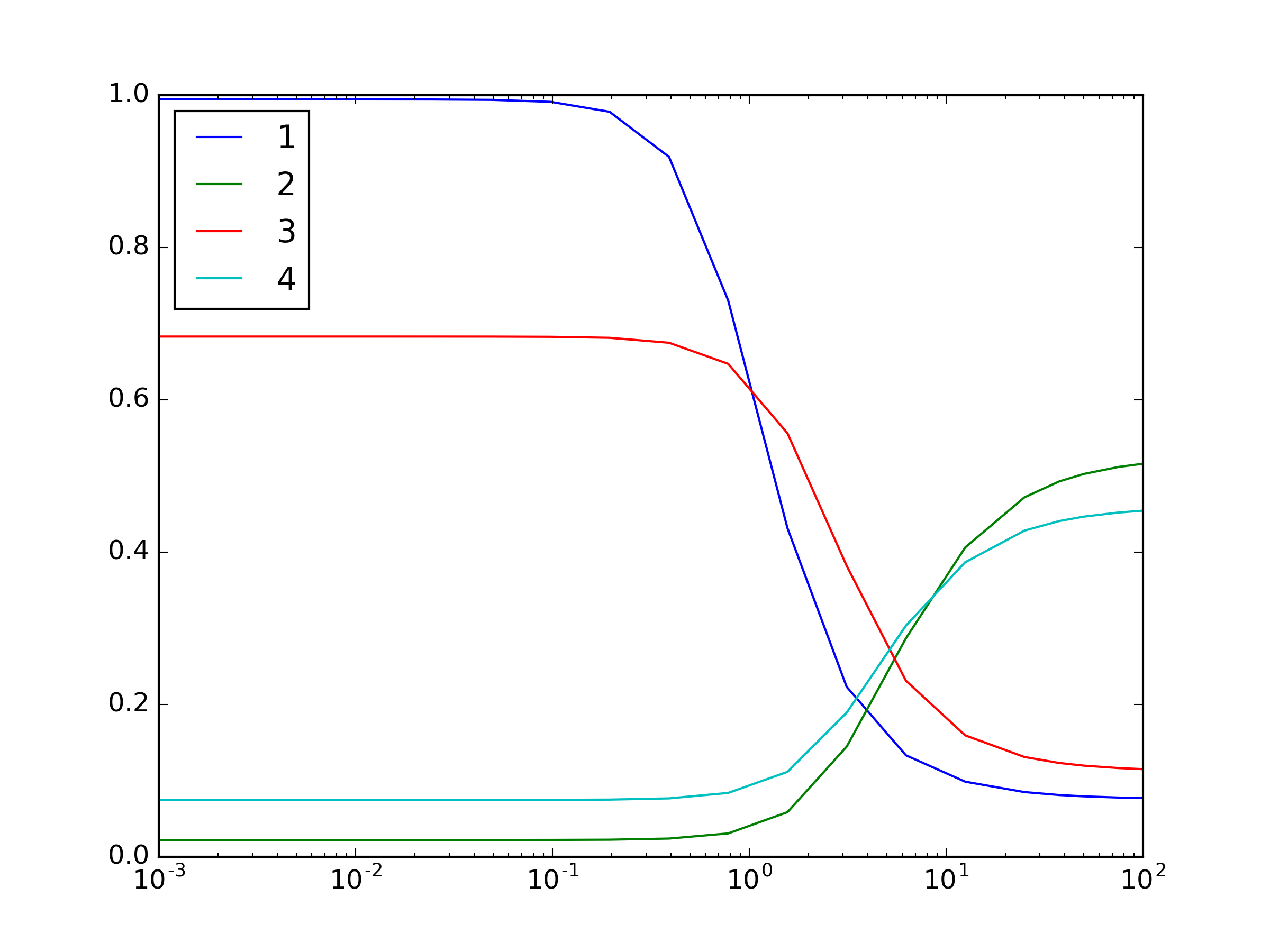

Supplement: Supplementary Software 1 — R cytometry data processing scripts and mathematical modeling scripts [file ncomms15459-s3.zip › Supplementary Software 1/FittingScripts/Results/Output/FittingScript_DoseExp2_20160330.py_model_image_2016-04-03-20-58-22_1459742302030100.png]

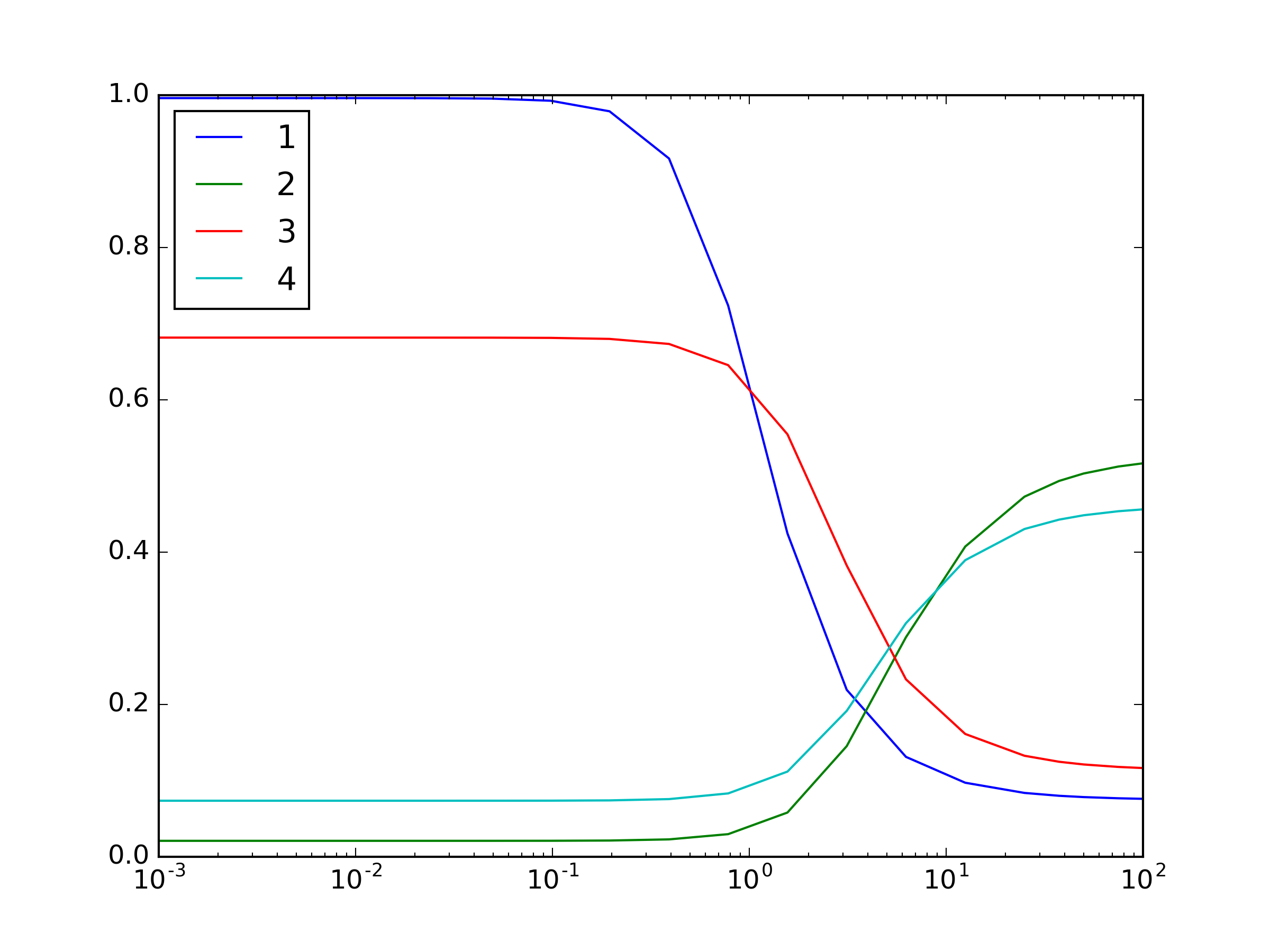

Supplement: Supplementary Software 1 — R cytometry data processing scripts and mathematical modeling scripts [file ncomms15459-s3.zip › Supplementary Software 1/FittingScripts/Results/Output/FittingScript_DoseExp2_20160330.py_model_image_2016-04-04-01-58-49_1459760329041702.png]

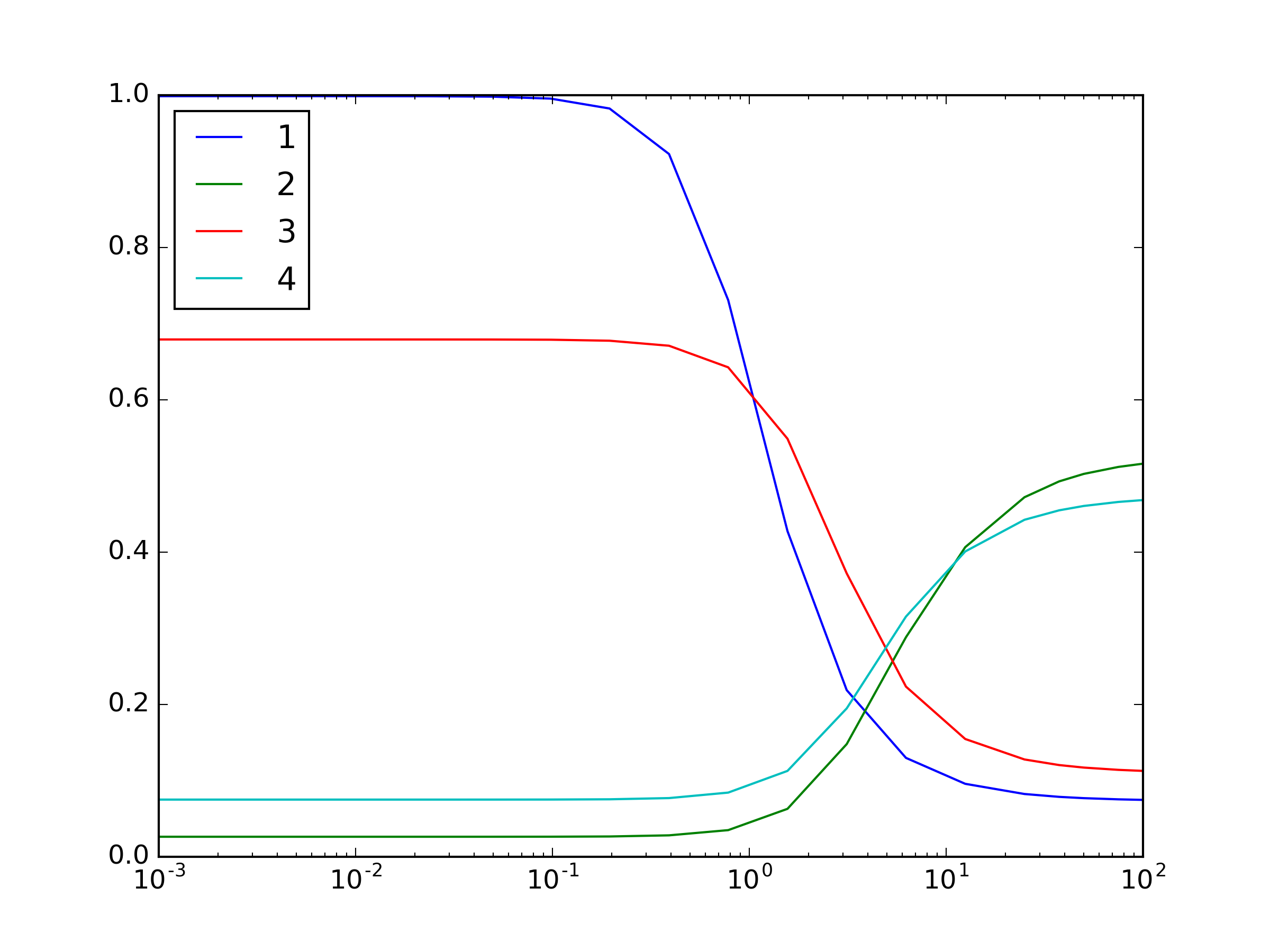

Supplement: Supplementary Software 1 — R cytometry data processing scripts and mathematical modeling scripts [file ncomms15459-s3.zip › Supplementary Software 1/FittingScripts/Results/Output/FittingScript_DoseExp2_20160330.py_model_image_2016-04-04-06-49-25_1459777765712797.png]

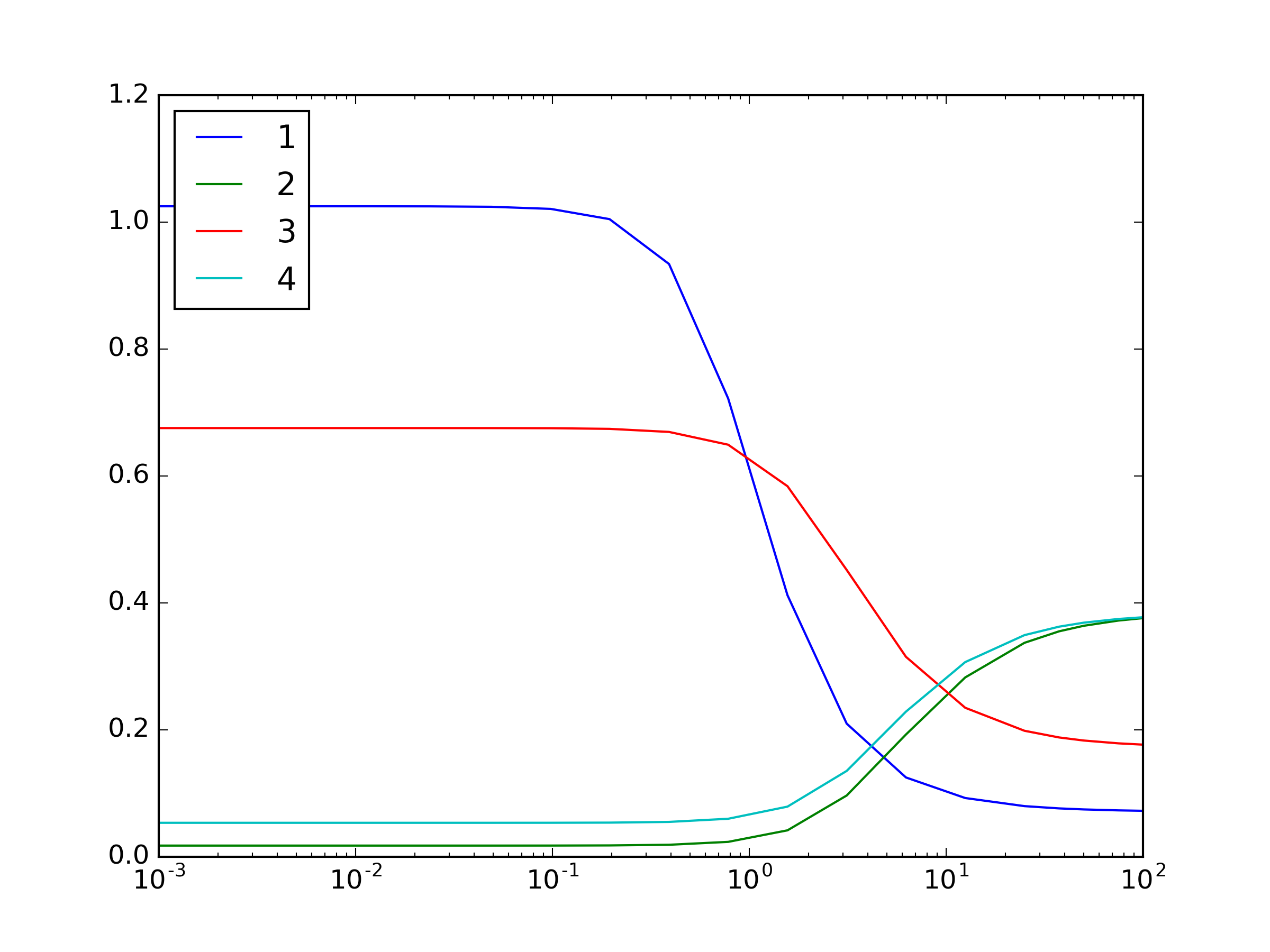

Supplement: Supplementary Software 1 — R cytometry data processing scripts and mathematical modeling scripts [file ncomms15459-s3.zip › Supplementary Software 1/FittingScripts/Results/Output/FittingScript_DoseExp3_20160330.py_model_image_2016-04-01-12-26-52_1459538812737452.png]

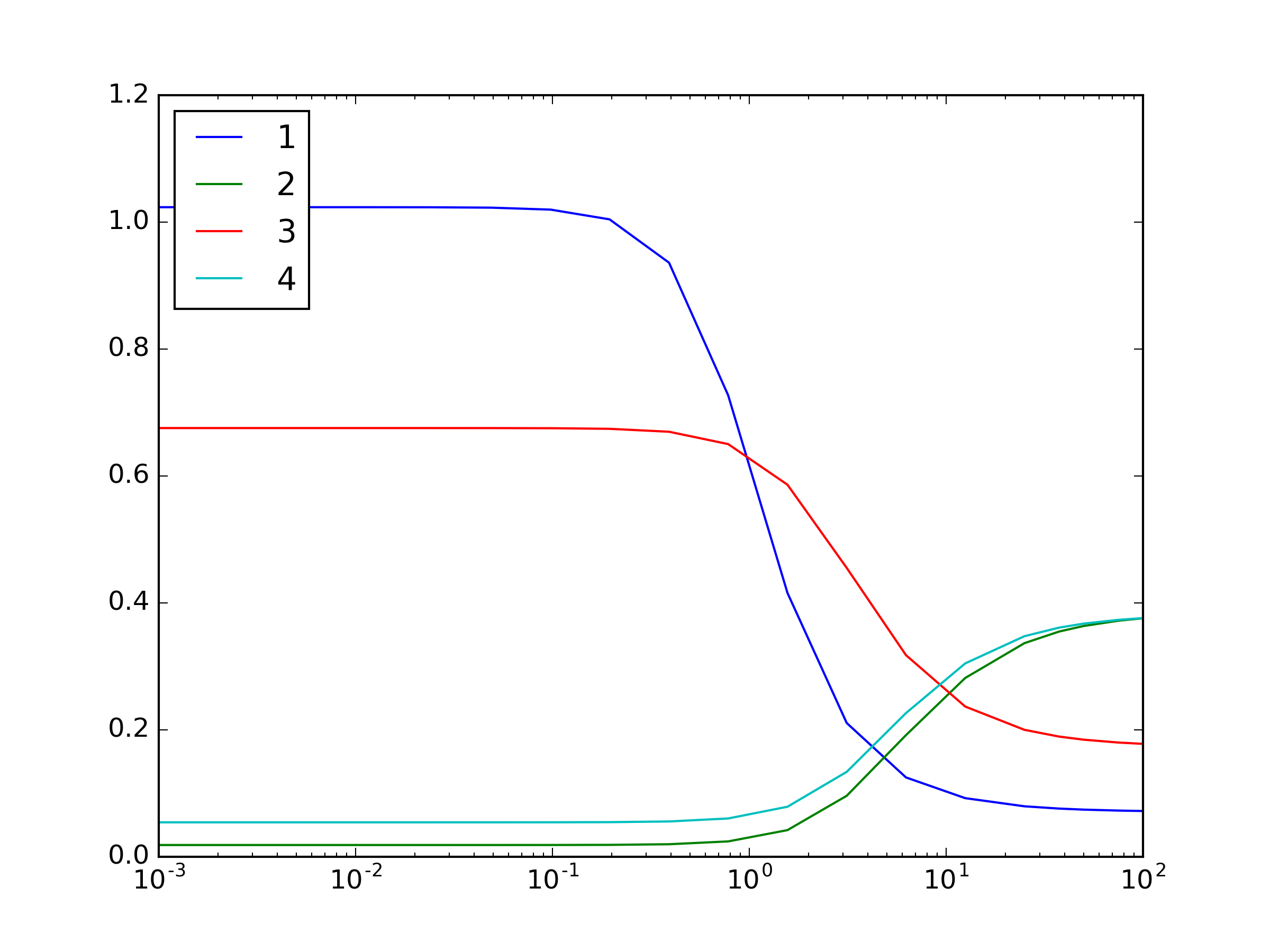

Supplement: Supplementary Software 1 — R cytometry data processing scripts and mathematical modeling scripts [file ncomms15459-s3.zip › Supplementary Software 1/FittingScripts/Results/Output/FittingScript_DoseExp3_20160330.py_model_image_2016-04-01-17-01-35_1459555295622418.png]

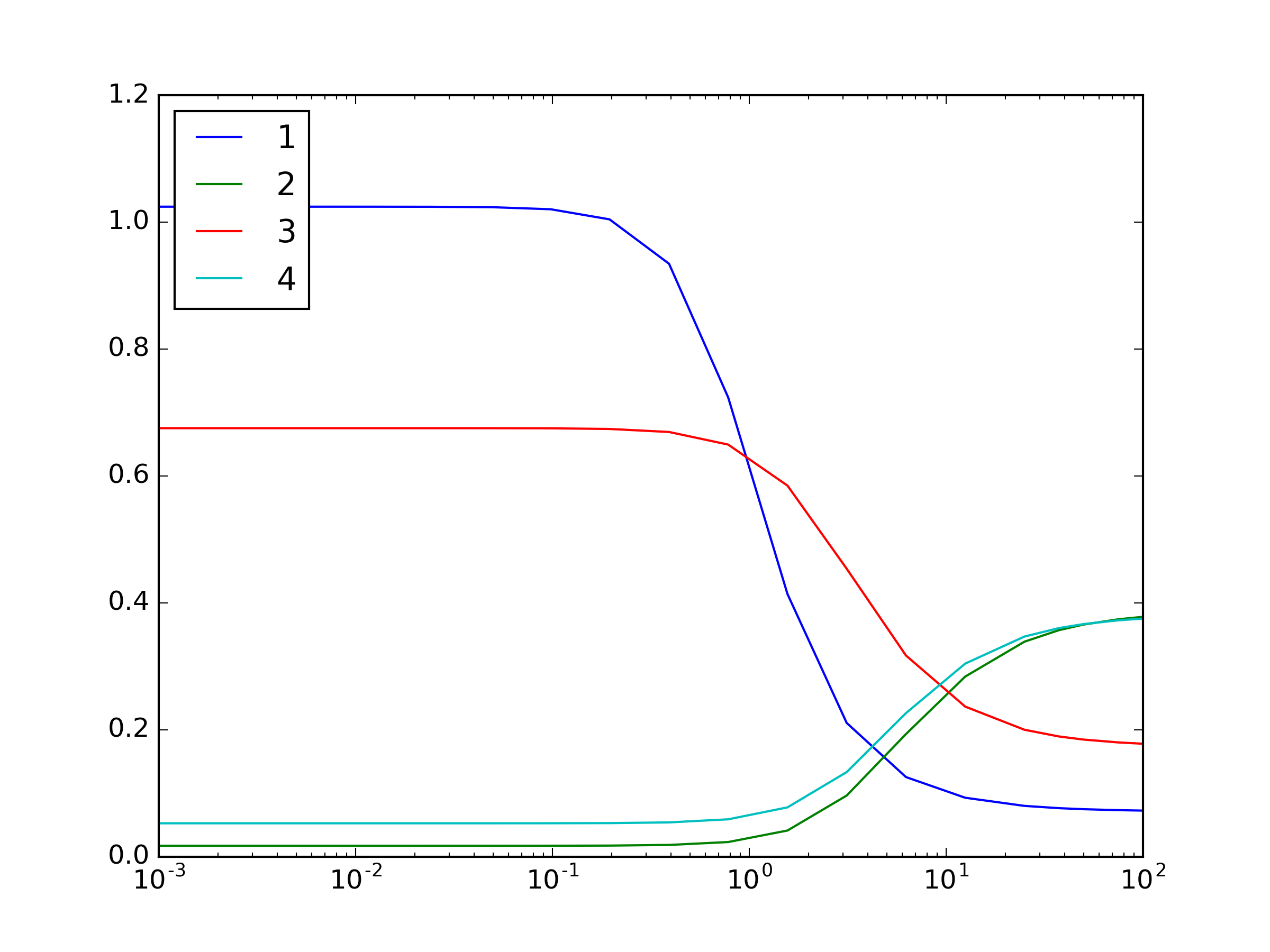

Supplement: Supplementary Software 1 — R cytometry data processing scripts and mathematical modeling scripts [file ncomms15459-s3.zip › Supplementary Software 1/FittingScripts/Results/Output/FittingScript_DoseExp3_20160330.py_model_image_2016-04-01-19-34-23_1459564463699416.png]

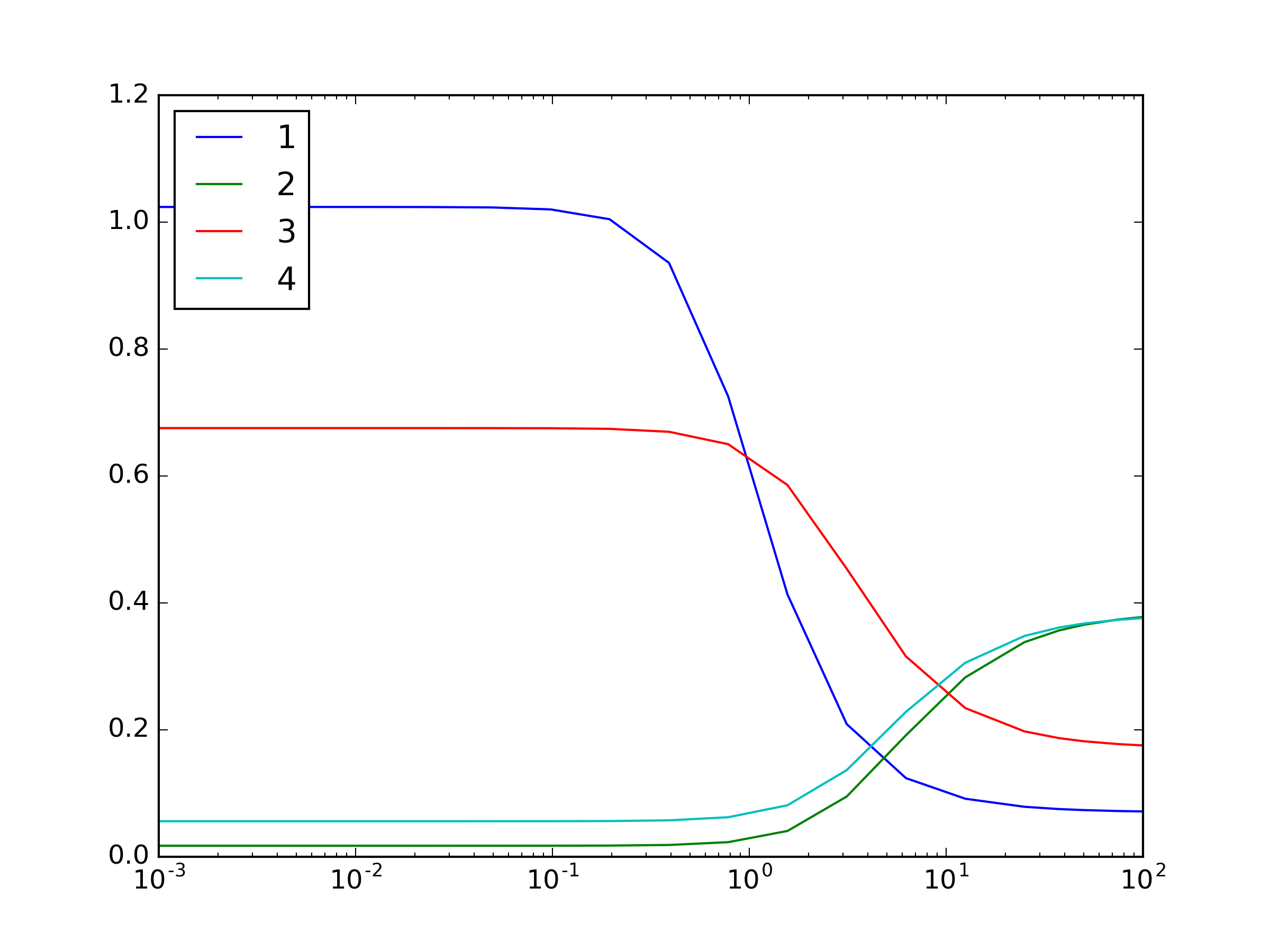

Supplement: Supplementary Software 1 — R cytometry data processing scripts and mathematical modeling scripts [file ncomms15459-s3.zip › Supplementary Software 1/FittingScripts/Results/Output/FittingScript_DoseExp3_20160330.py_model_image_2016-04-01-22-33-12_1459575192191198.png]

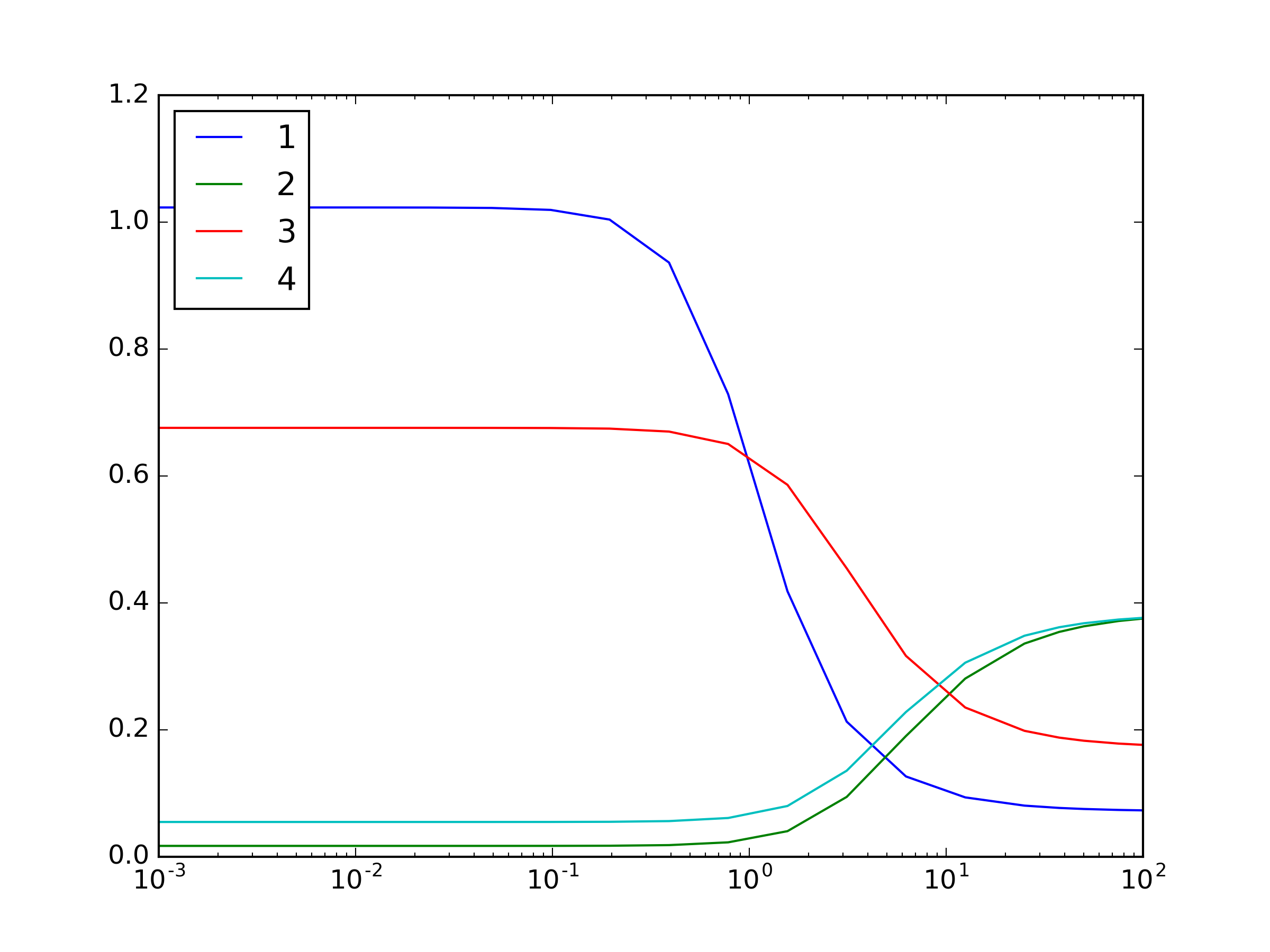

Supplement: Supplementary Software 1 — R cytometry data processing scripts and mathematical modeling scripts [file ncomms15459-s3.zip › Supplementary Software 1/FittingScripts/Results/Output/FittingScript_DoseExp3_20160330.py_model_image_2016-04-02-02-24-34_1459589074500830.png]

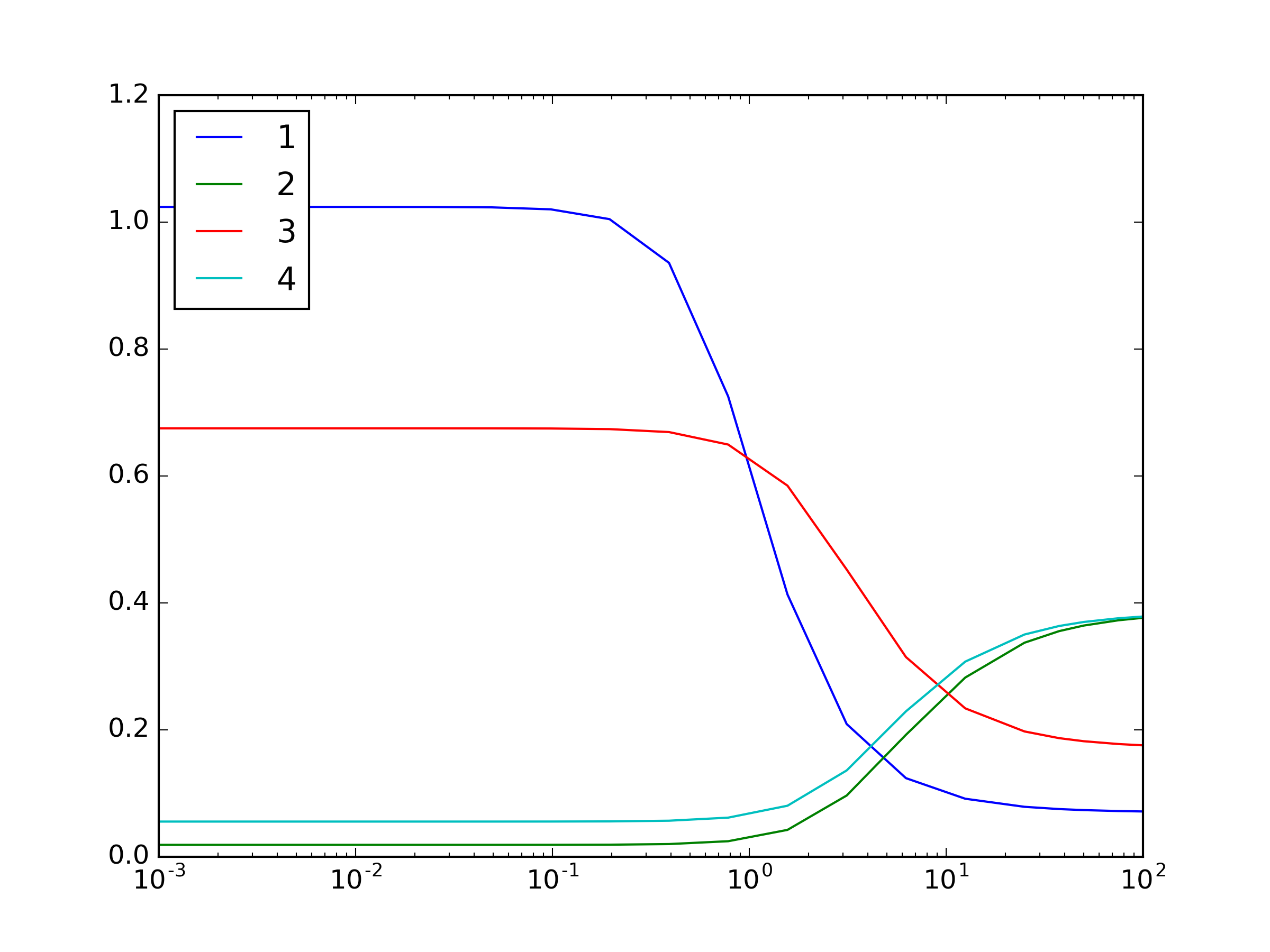

Supplement: Supplementary Software 1 — R cytometry data processing scripts and mathematical modeling scripts [file ncomms15459-s3.zip › Supplementary Software 1/FittingScripts/Results/Output/FittingScript_DoseExp3_20160330.py_model_image_2016-04-02-05-37-05_1459600625505524.png]

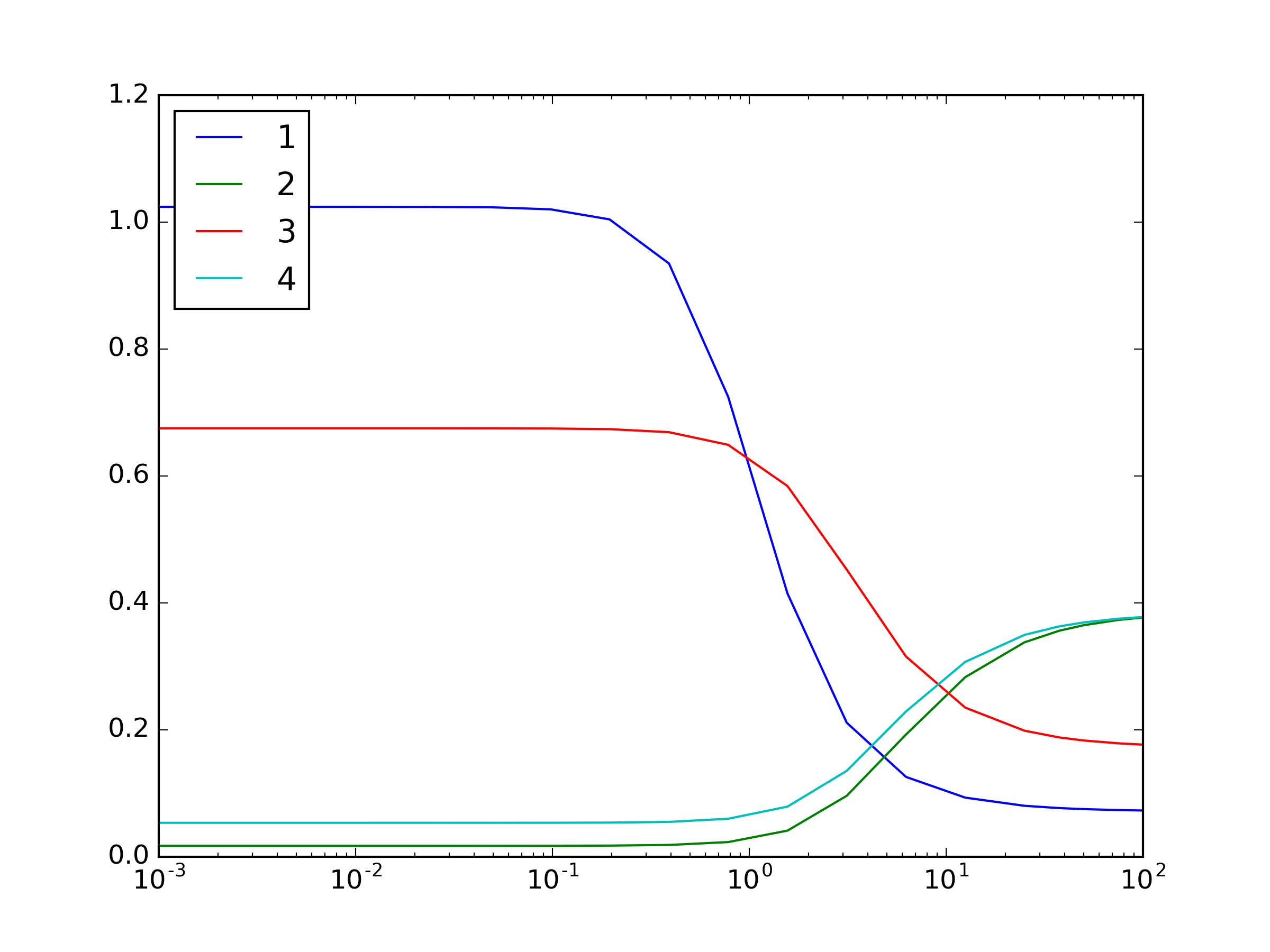

Supplement: Supplementary Software 1 — R cytometry data processing scripts and mathematical modeling scripts [file ncomms15459-s3.zip › Supplementary Software 1/FittingScripts/Results/Output/FittingScript_DoseExp3_20160330.py_model_image_2016-04-02-08-59-53_1459612793640802.png]

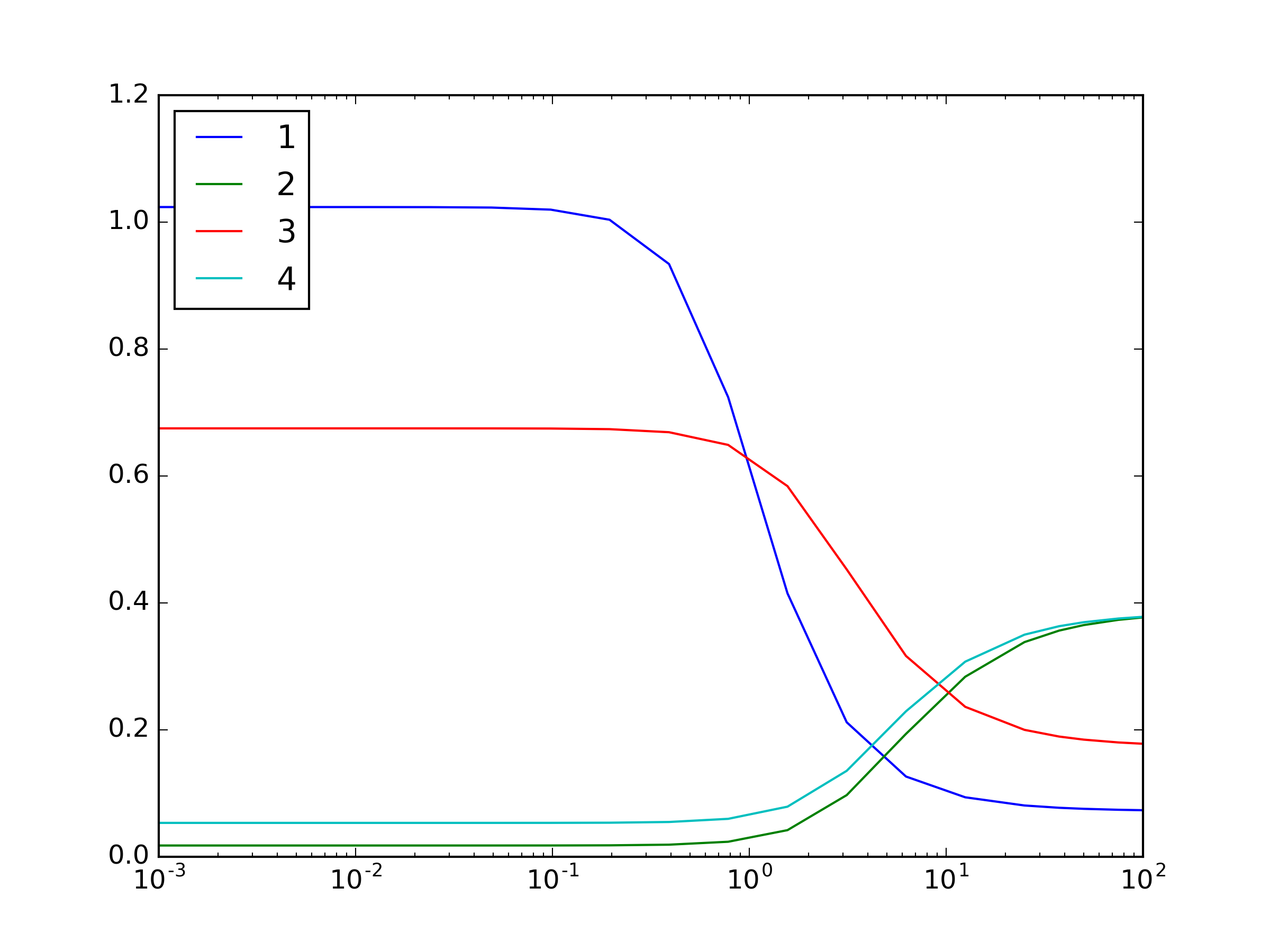

Supplement: Supplementary Software 1 — R cytometry data processing scripts and mathematical modeling scripts [file ncomms15459-s3.zip › Supplementary Software 1/FittingScripts/Results/Output/FittingScript_DoseExp3_20160330.py_model_image_2016-04-02-12-09-33_1459624173742716.png]

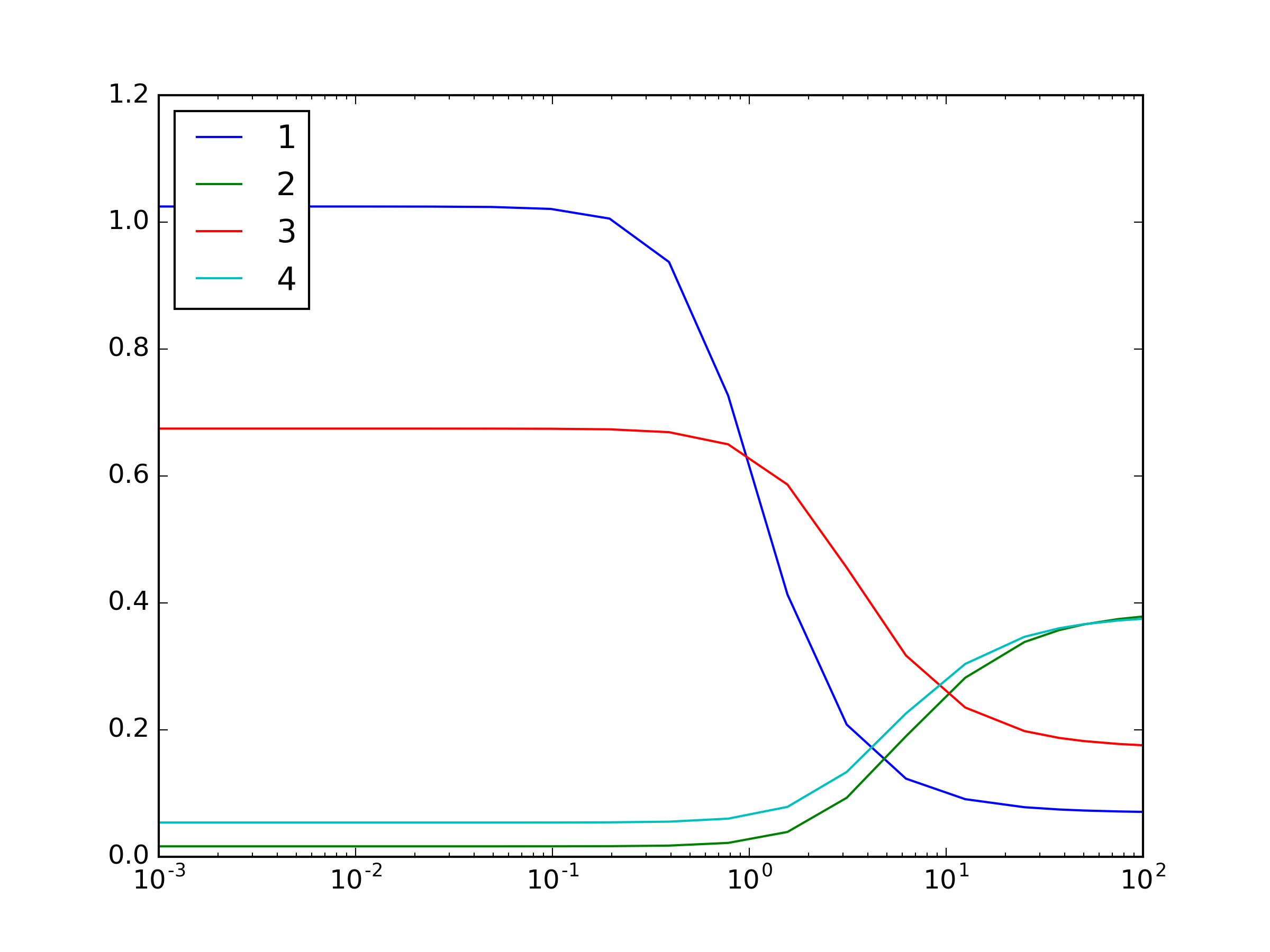

Supplement: Supplementary Software 1 — R cytometry data processing scripts and mathematical modeling scripts [file ncomms15459-s3.zip › Supplementary Software 1/FittingScripts/Results/Output/FittingScript_DoseExp3_20160330.py_model_image_2016-04-02-15-29-51_1459636191234952.png]

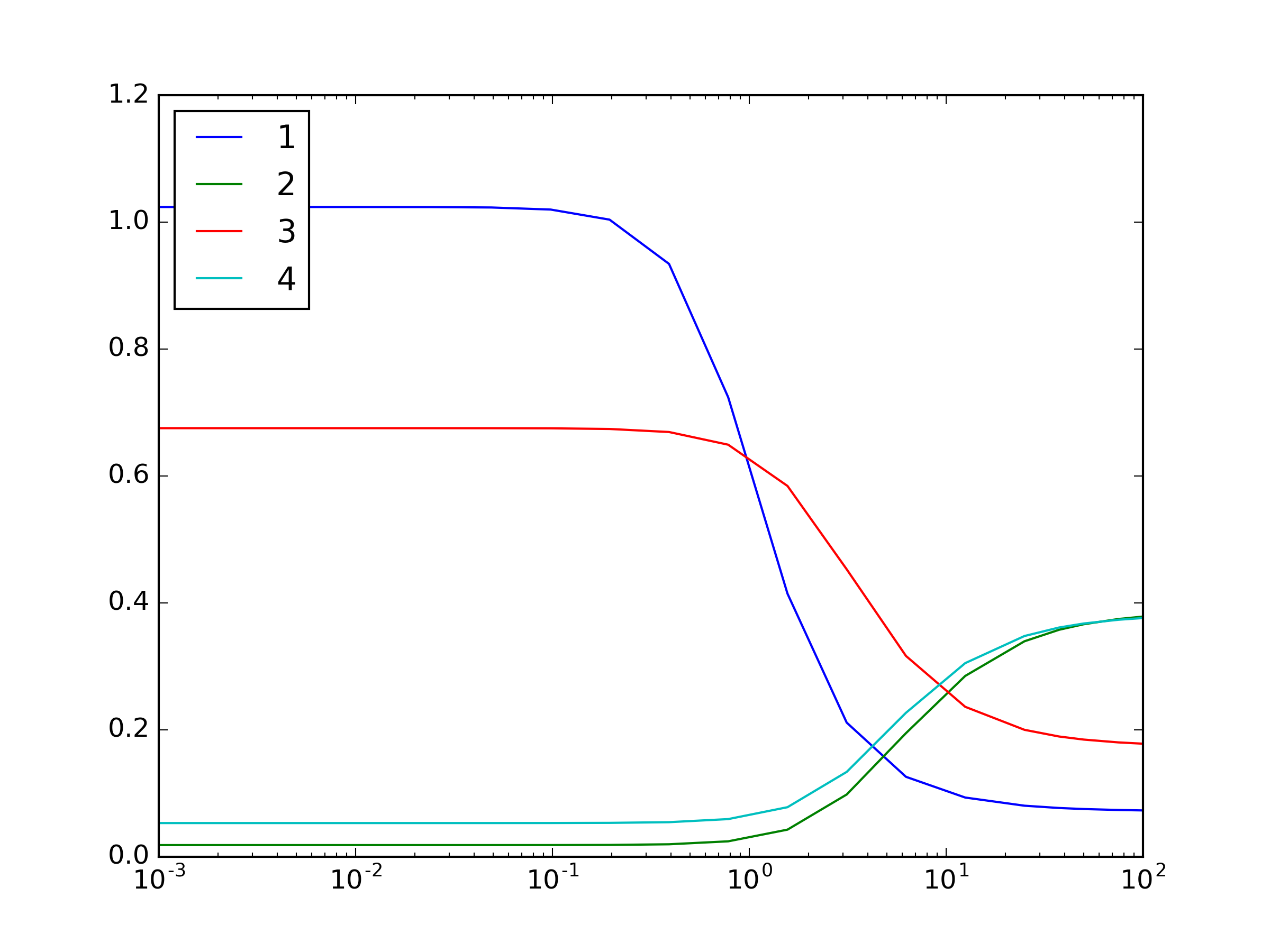

Supplement: Supplementary Software 1 — R cytometry data processing scripts and mathematical modeling scripts [file ncomms15459-s3.zip › Supplementary Software 1/FittingScripts/Results/Output/FittingScript_DoseExp3_20160330.py_model_image_2016-04-02-18-32-13_1459647133625355.png]

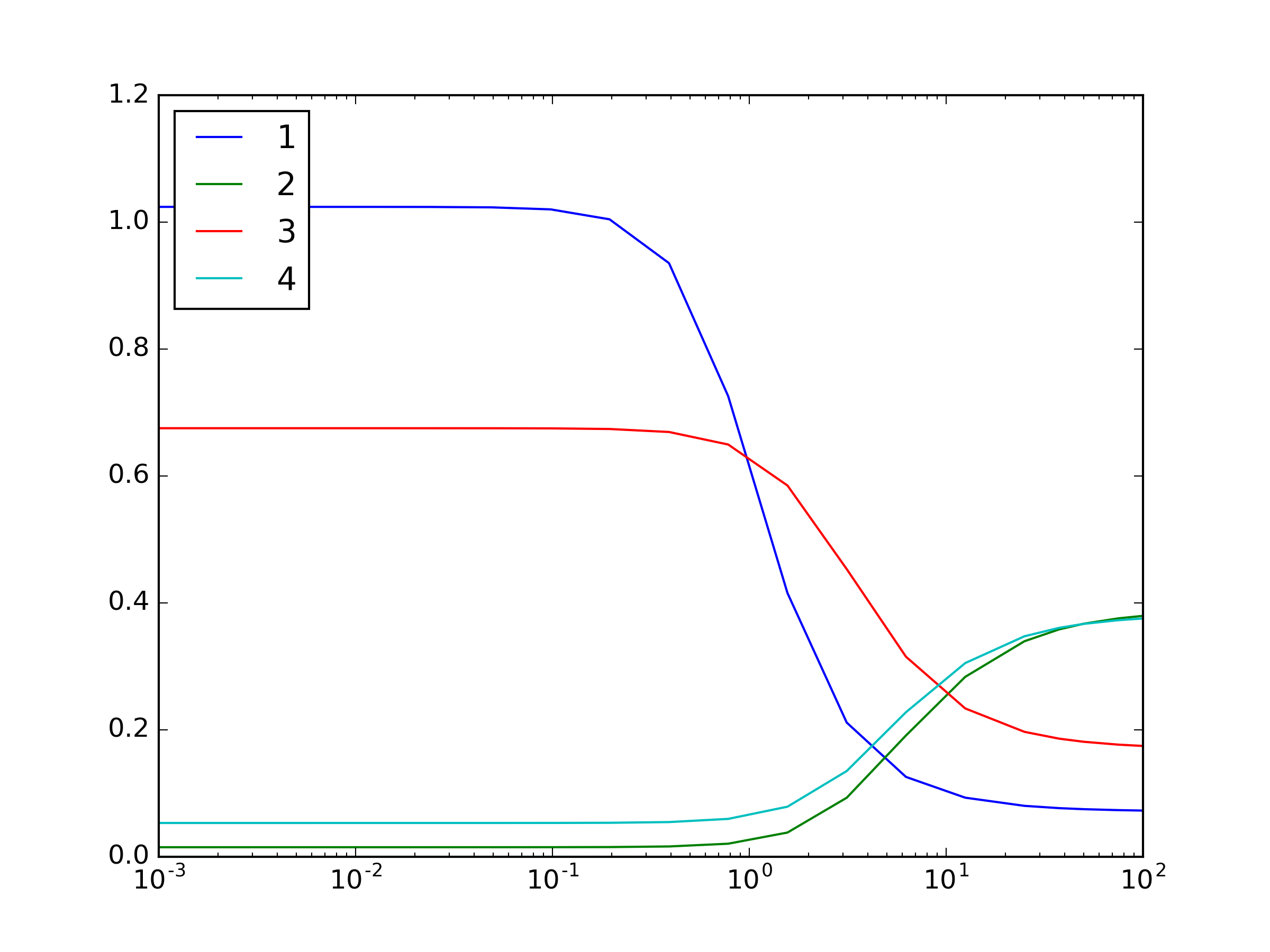

Supplement: Supplementary Software 1 — R cytometry data processing scripts and mathematical modeling scripts [file ncomms15459-s3.zip › Supplementary Software 1/FittingScripts/Results/Output/FittingScript_DoseExp3_20160330.py_model_image_2016-04-02-21-36-05_1459658165038971.png]

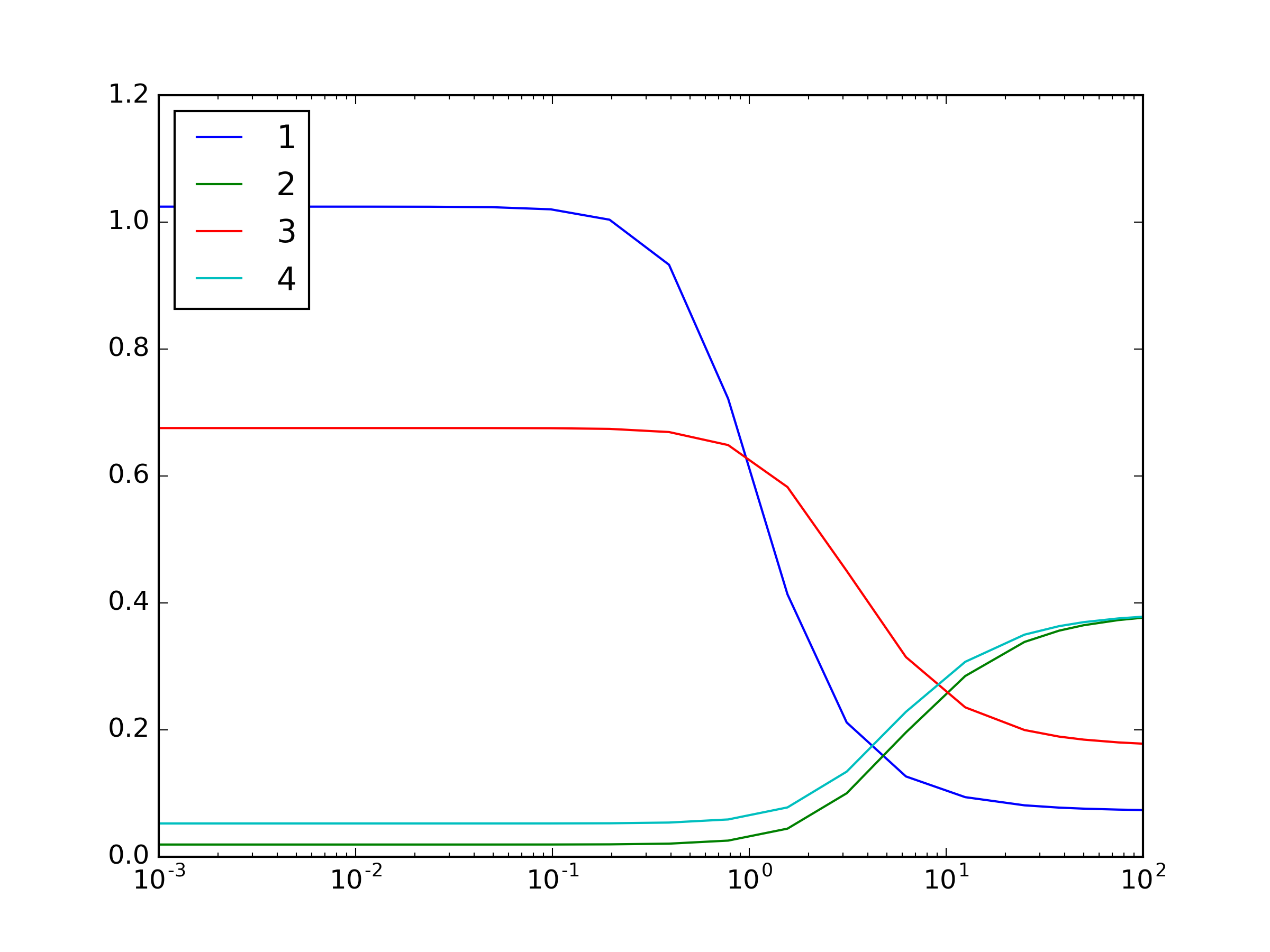

Supplement: Supplementary Software 1 — R cytometry data processing scripts and mathematical modeling scripts [file ncomms15459-s3.zip › Supplementary Software 1/FittingScripts/Results/Output/FittingScript_DoseExp3_20160330.py_model_image_2016-04-03-02-29-52_1459675792975787.png]

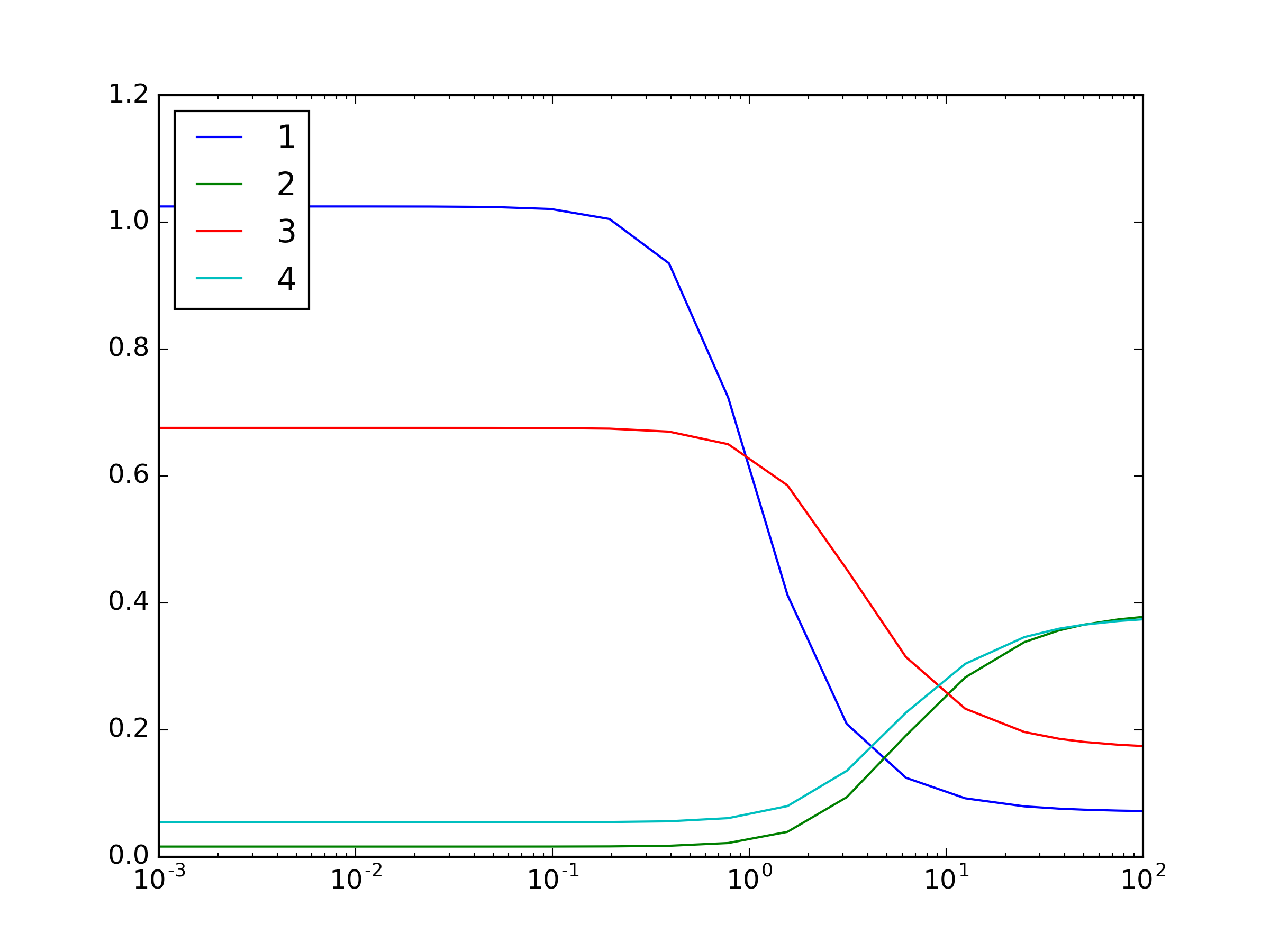

Supplement: Supplementary Software 1 — R cytometry data processing scripts and mathematical modeling scripts [file ncomms15459-s3.zip › Supplementary Software 1/FittingScripts/Results/Output/FittingScript_DoseExp3_20160330.py_model_image_2016-04-03-05-50-29_1459687829480170.png]

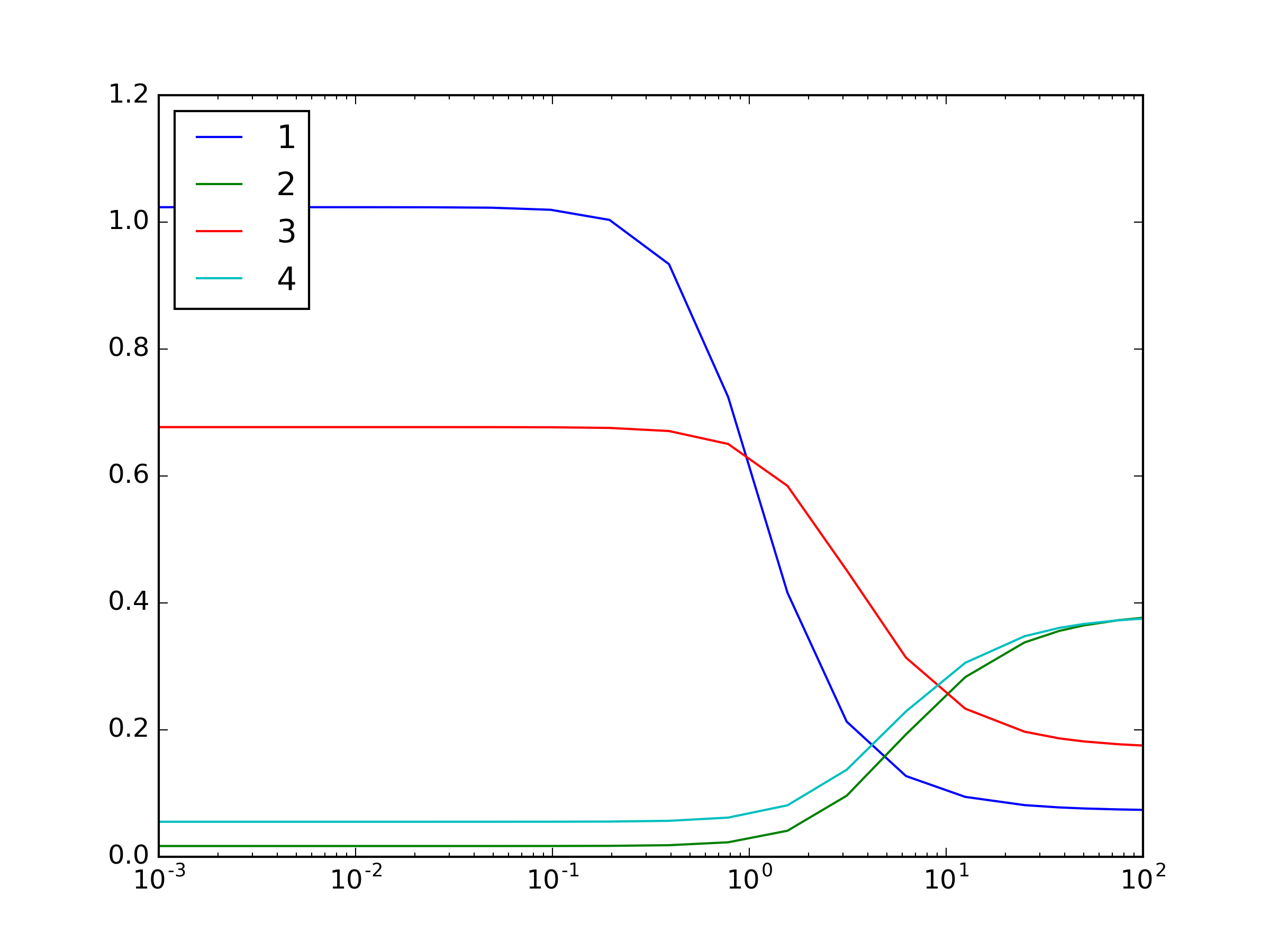

Supplement: Supplementary Software 1 — R cytometry data processing scripts and mathematical modeling scripts [file ncomms15459-s3.zip › Supplementary Software 1/FittingScripts/Results/Output/FittingScript_DoseExp3_20160330.py_model_image_2016-04-03-09-42-04_1459701724976278.png]

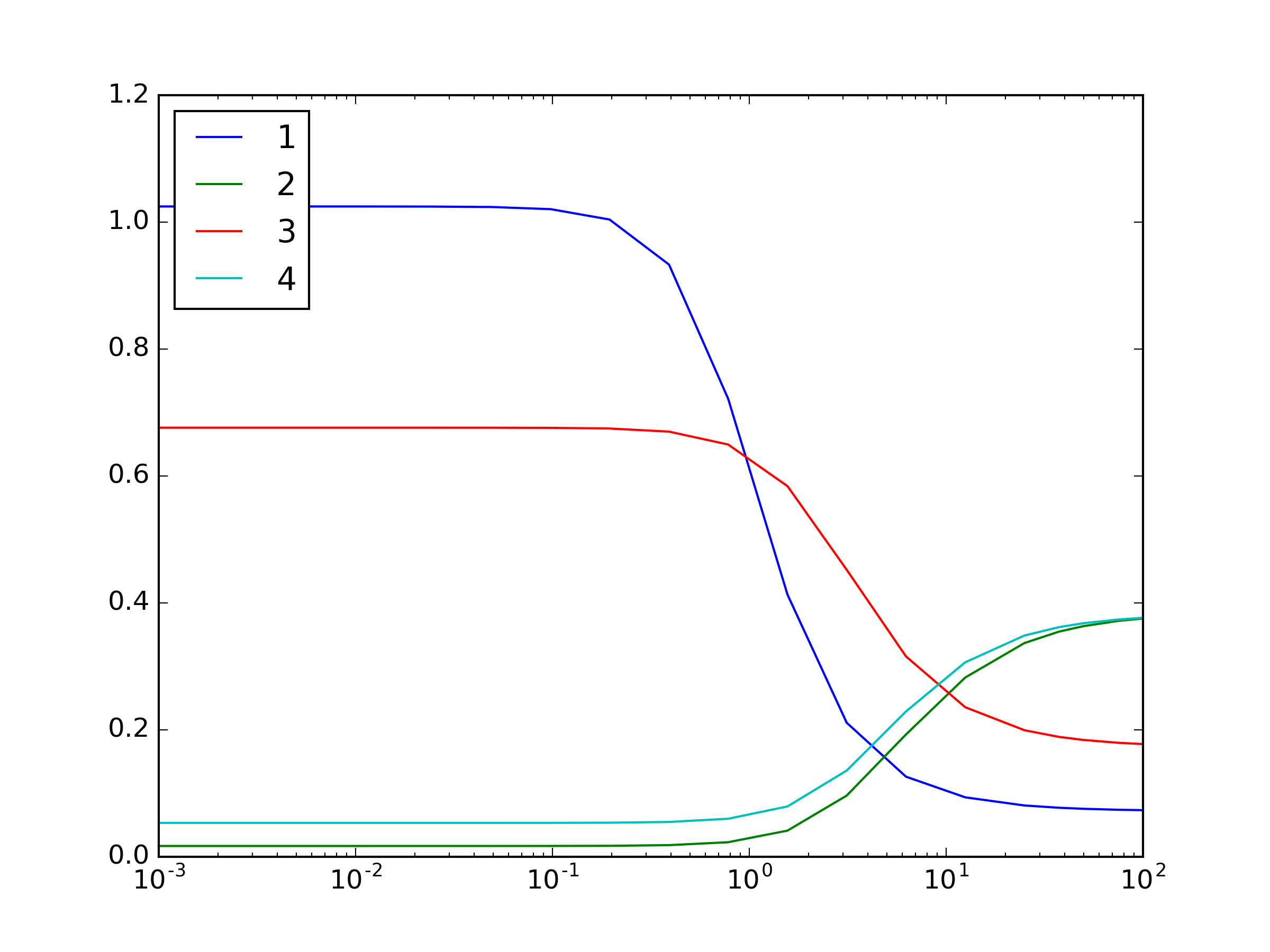

Supplement: Supplementary Software 1 — R cytometry data processing scripts and mathematical modeling scripts [file ncomms15459-s3.zip › Supplementary Software 1/FittingScripts/Results/Output/FittingScript_DoseExp3_20160330.py_model_image_2016-04-03-12-33-27_1459712007464696.png]

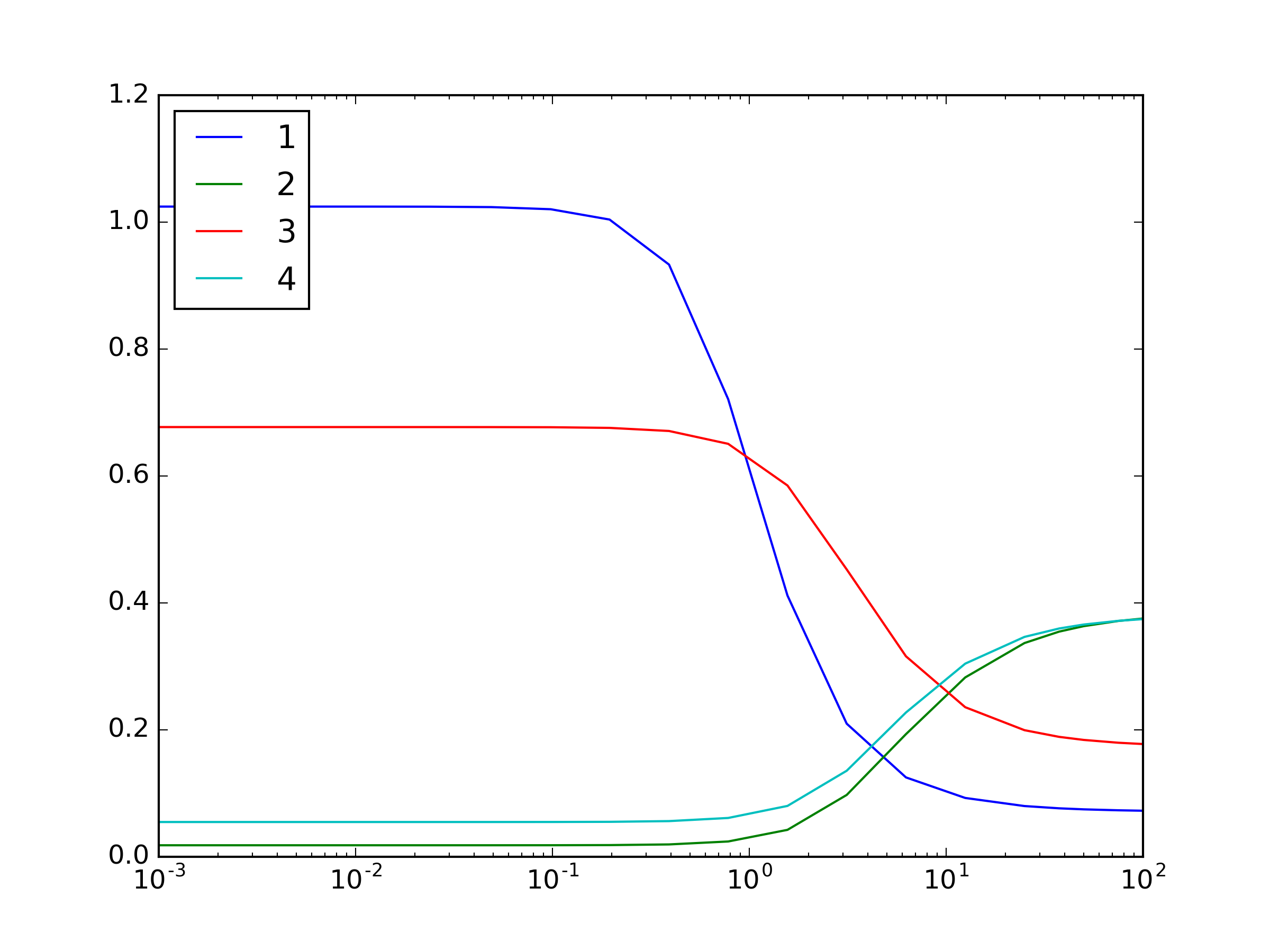

Supplement: Supplementary Software 1 — R cytometry data processing scripts and mathematical modeling scripts [file ncomms15459-s3.zip › Supplementary Software 1/FittingScripts/Results/Output/FittingScript_DoseExp3_20160330.py_model_image_2016-04-03-15-27-53_1459722473591413.png]

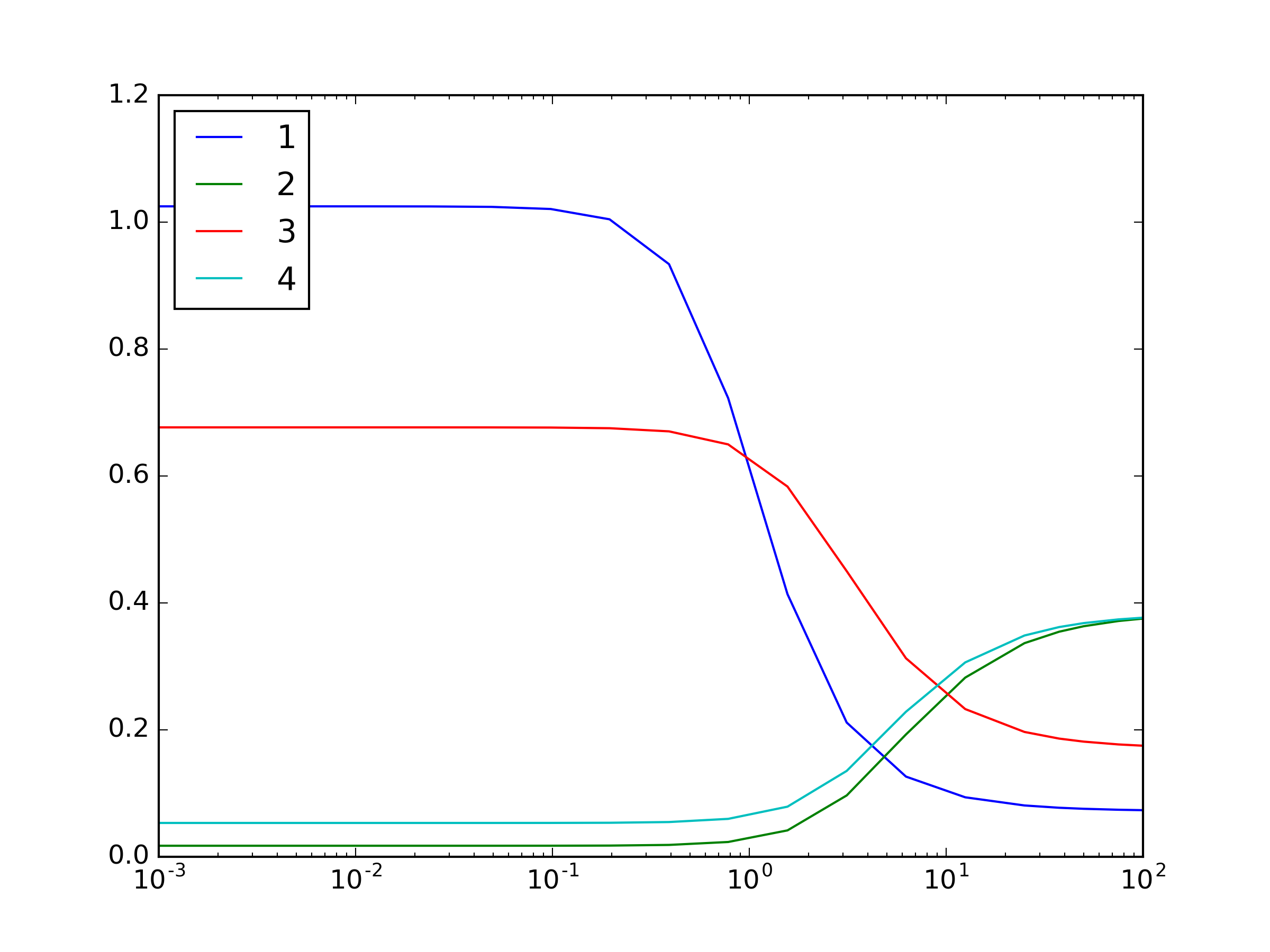

Supplement: Supplementary Software 1 — R cytometry data processing scripts and mathematical modeling scripts [file ncomms15459-s3.zip › Supplementary Software 1/FittingScripts/Results/Output/FittingScript_DoseExp3_20160330.py_model_image_2016-04-03-18-19-51_1459732791911169.png]

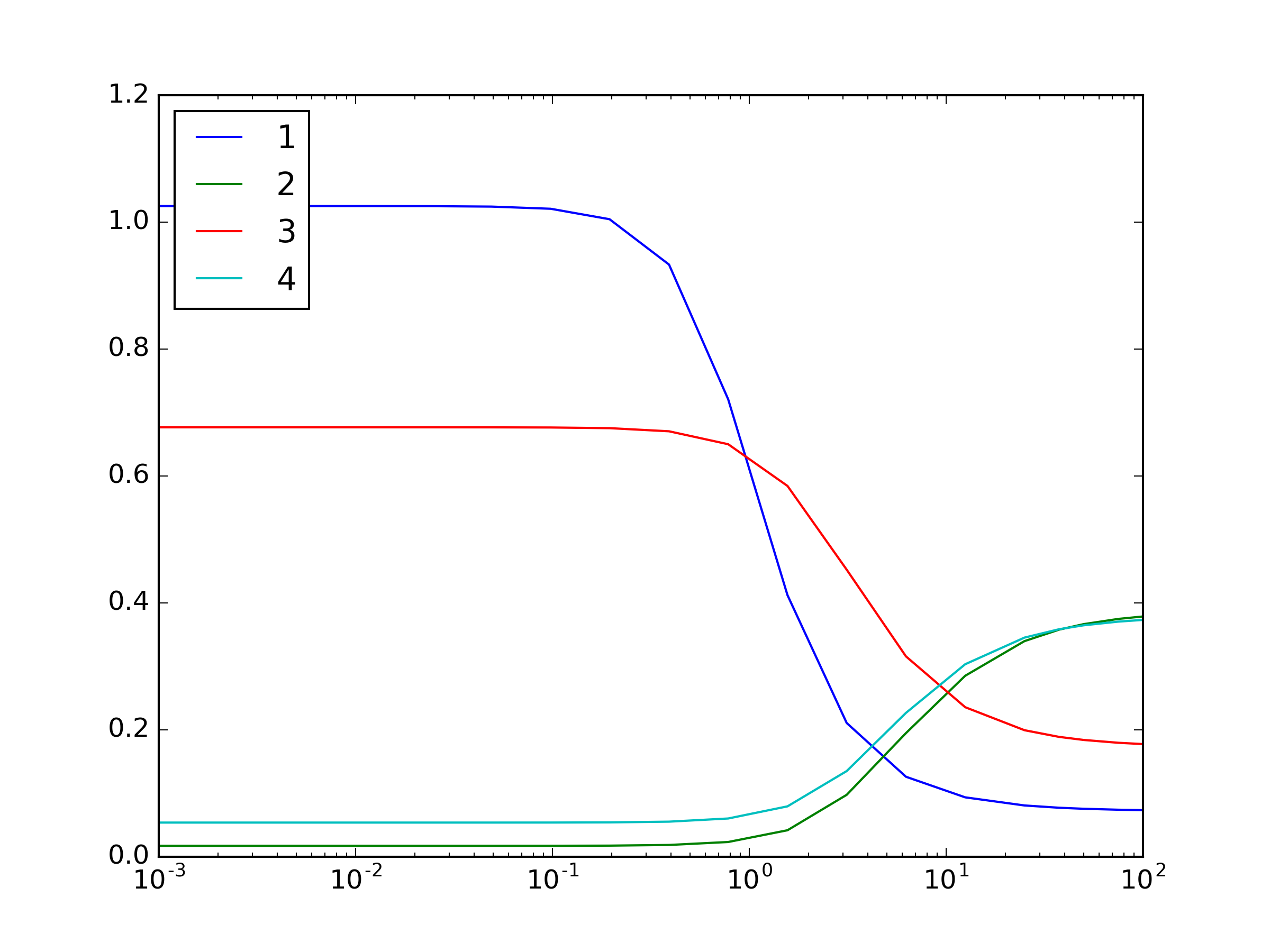

Supplement: Supplementary Software 1 — R cytometry data processing scripts and mathematical modeling scripts [file ncomms15459-s3.zip › Supplementary Software 1/FittingScripts/Results/Output/FittingScript_DoseExp3_20160330.py_model_image_2016-04-03-21-45-09_1459745109829235.png]

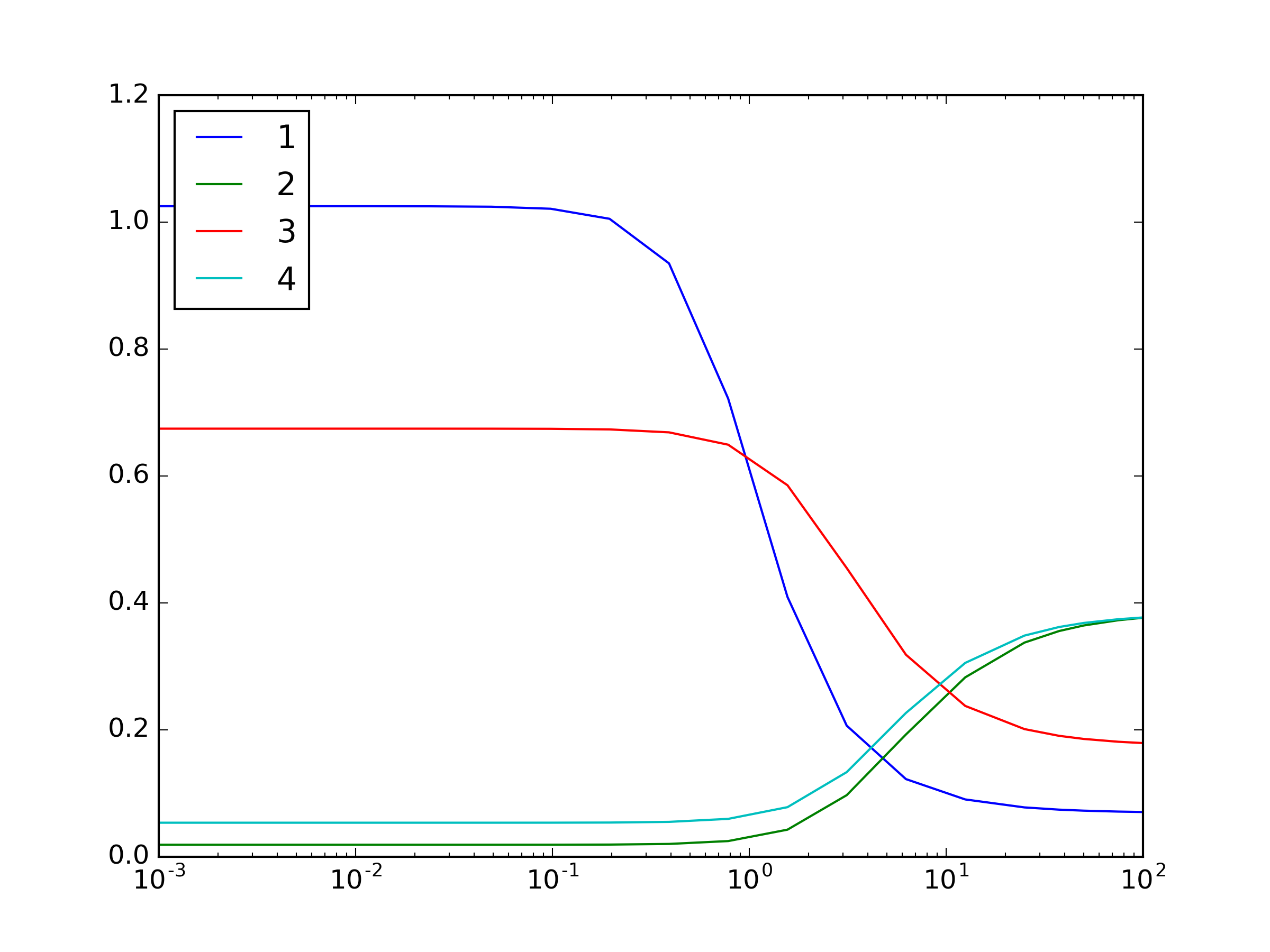

Supplement: Supplementary Software 1 — R cytometry data processing scripts and mathematical modeling scripts [file ncomms15459-s3.zip › Supplementary Software 1/FittingScripts/Results/Output/FittingScript_DoseExp3_20160330.py_model_image_2016-04-04-00-27-37_1459754857945709.png]

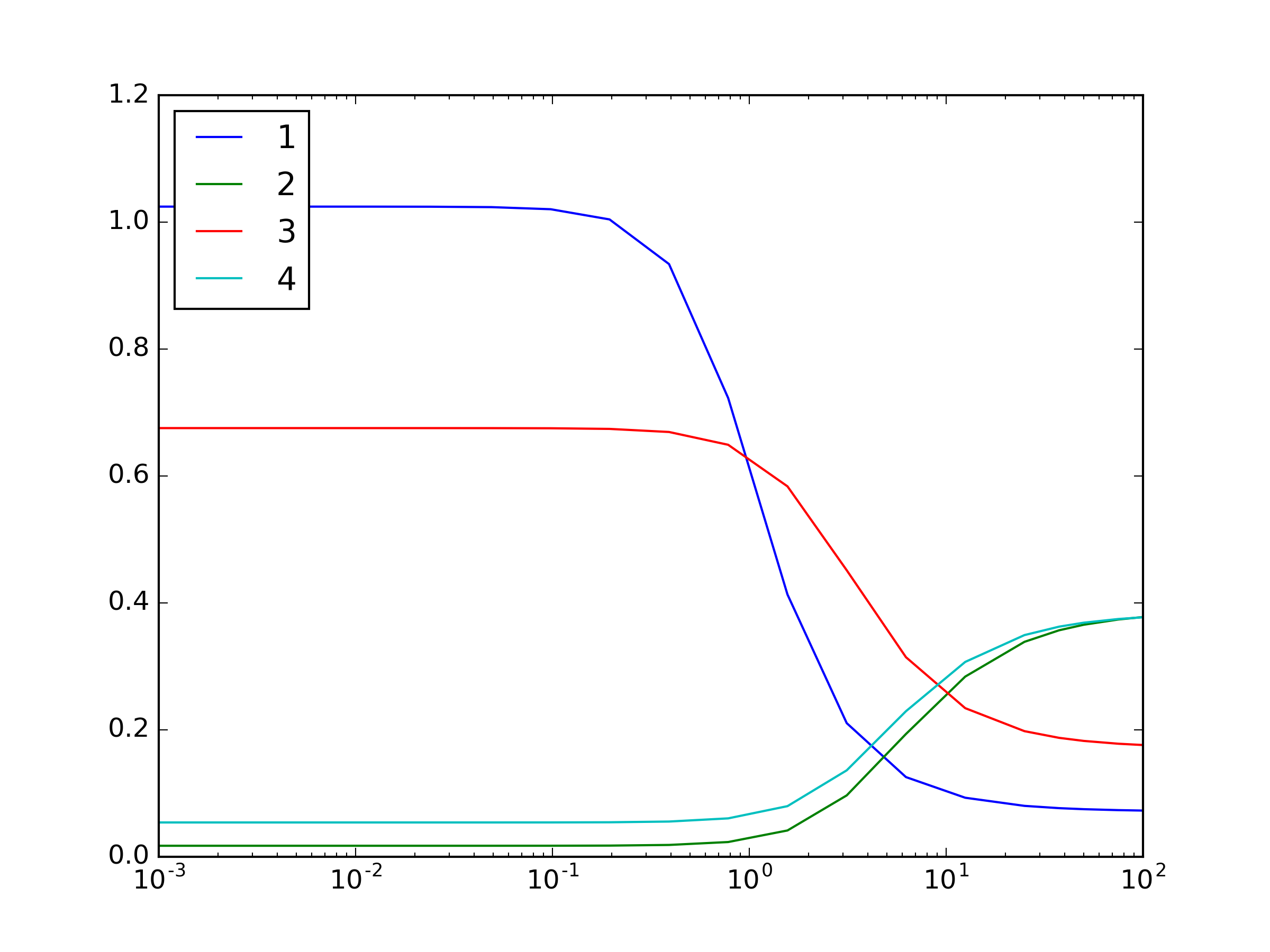

Supplement: Supplementary Software 1 — R cytometry data processing scripts and mathematical modeling scripts [file ncomms15459-s3.zip › Supplementary Software 1/FittingScripts/Results/Output/FittingScript_DoseExp3_20160330.py_model_image_2016-04-04-02-57-06_1459763826329661.png]

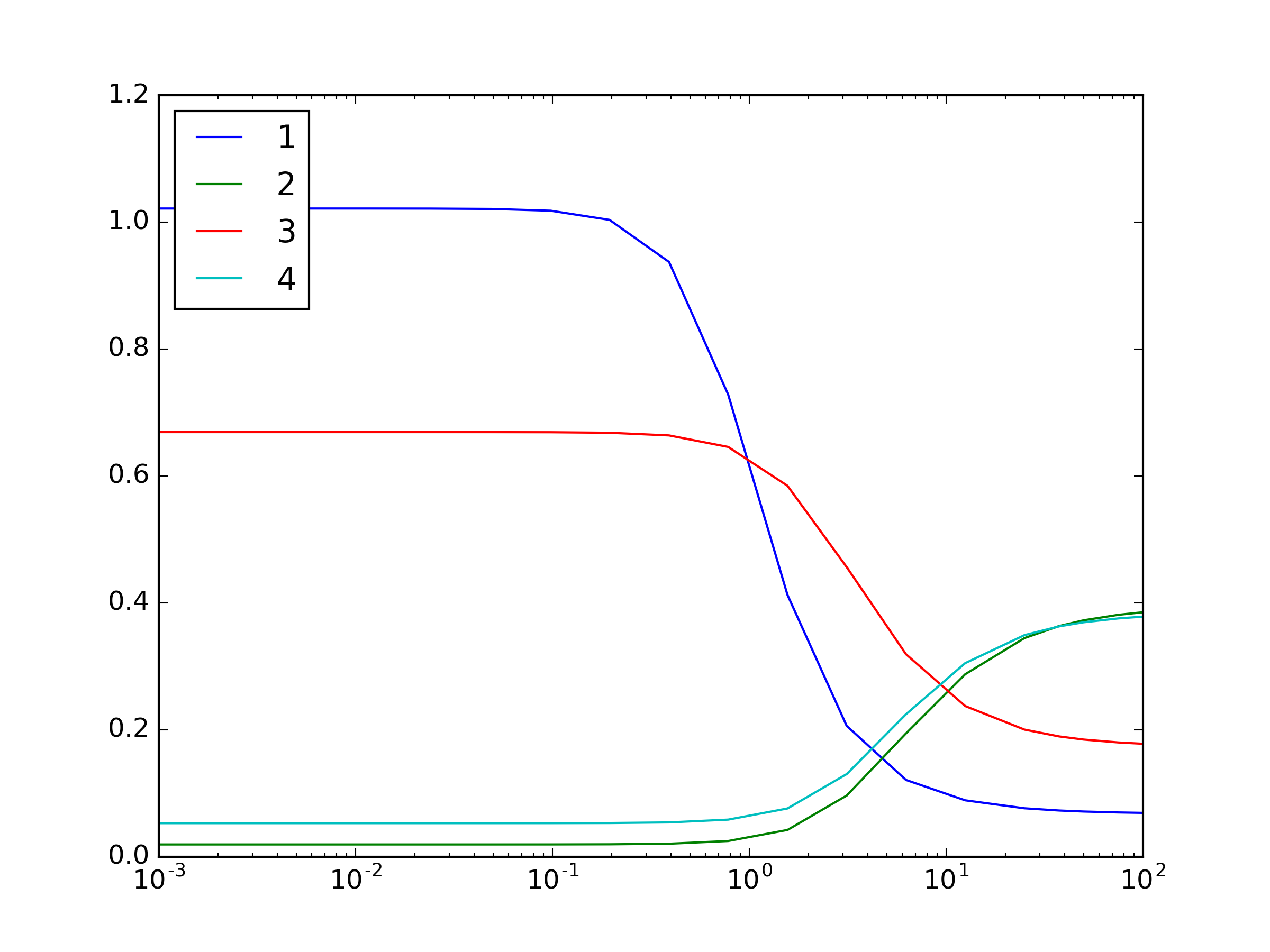

Supplement: Supplementary Software 1 — R cytometry data processing scripts and mathematical modeling scripts [file ncomms15459-s3.zip › Supplementary Software 1/FittingScripts/Results/Output/FittingScript_DoseExp3_20160330.py_model_image_2016-04-04-07-34-11_1459780451121117.png]
