# Supplementary material for: Digital logic circuits in yeast with CRISPR-dCas9 NOR gates
Source: Nat Commun. 2017 May 25;8:15459. doi: 10.1038/ncomms15459 (PMC5458518; doi:10.1038/ncomms15459)
Supplement: Supplementary Software 1 — R cytometry data processing scripts and mathematical modeling scripts [file ncomms15459-s3.zip › Supplementary Software 1/FittingScripts/Results/Output_20160415/Fitting_kinetics.pdf]

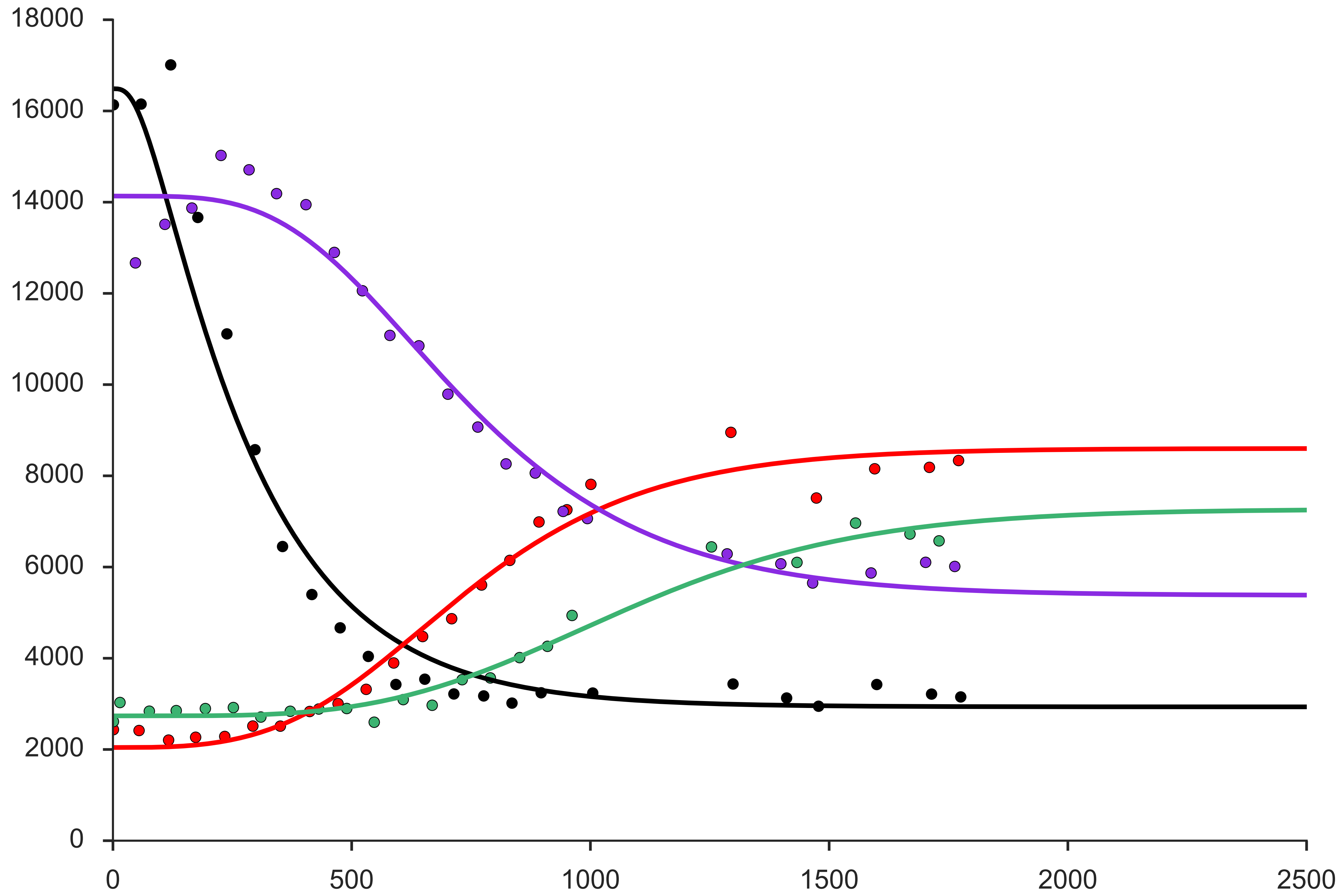

18000
16000
14000
12000
10000
8000
6000
4000
2000
0
0
500
1000
1500
2000
2500
