# Supplementary material for: Digital logic circuits in yeast with CRISPR-dCas9 NOR gates
Source: Nat Commun. 2017 May 25;8:15459. doi: 10.1038/ncomms15459 (PMC5458518; doi:10.1038/ncomms15459)
Supplement: Supplementary Software 1 — R cytometry data processing scripts and mathematical modeling scripts [file ncomms15459-s3.zip › Supplementary Software 1/FittingScripts/Results/Output_20160415/Fitting_steadystate.pdf]

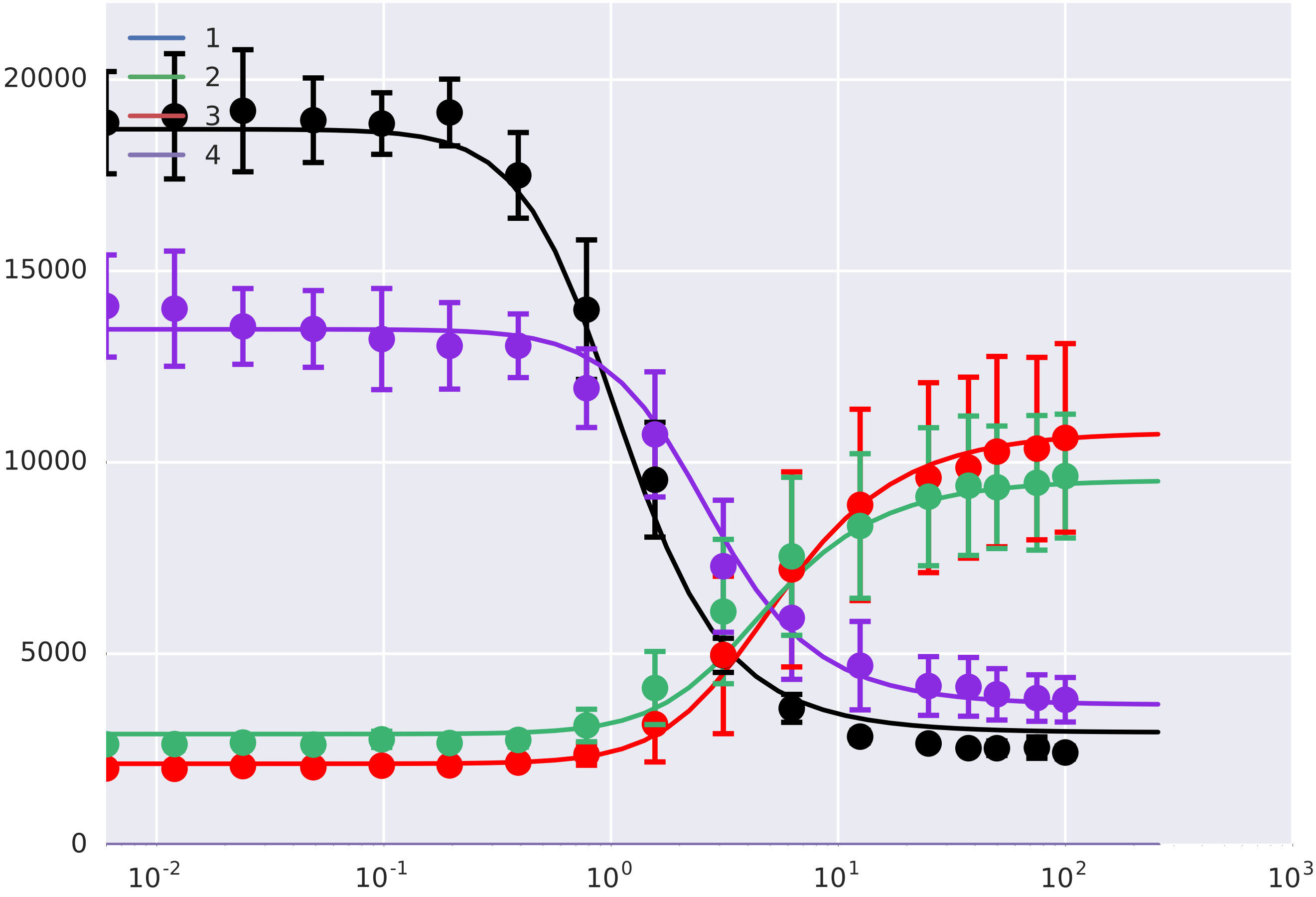
1
2
3
4
20000
15000
10000
5000
0
10^{-2}
10^{-1}
10^{0}
10^{1}
10^{2}
10^{3}
