# Supplementary material for: Digital logic circuits in yeast with CRISPR-dCas9 NOR gates
Source: Nat Commun. 2017 May 25;8:15459. doi: 10.1038/ncomms15459 (PMC5458518; doi:10.1038/ncomms15459)
Supplement: Supplementary Software 1 — R cytometry data processing scripts and mathematical modeling scripts [file ncomms15459-s3.zip › Supplementary Software 1/FittingScripts/Results/Output_20160415/PRCC_Rough_Fig_20160518.pdf]

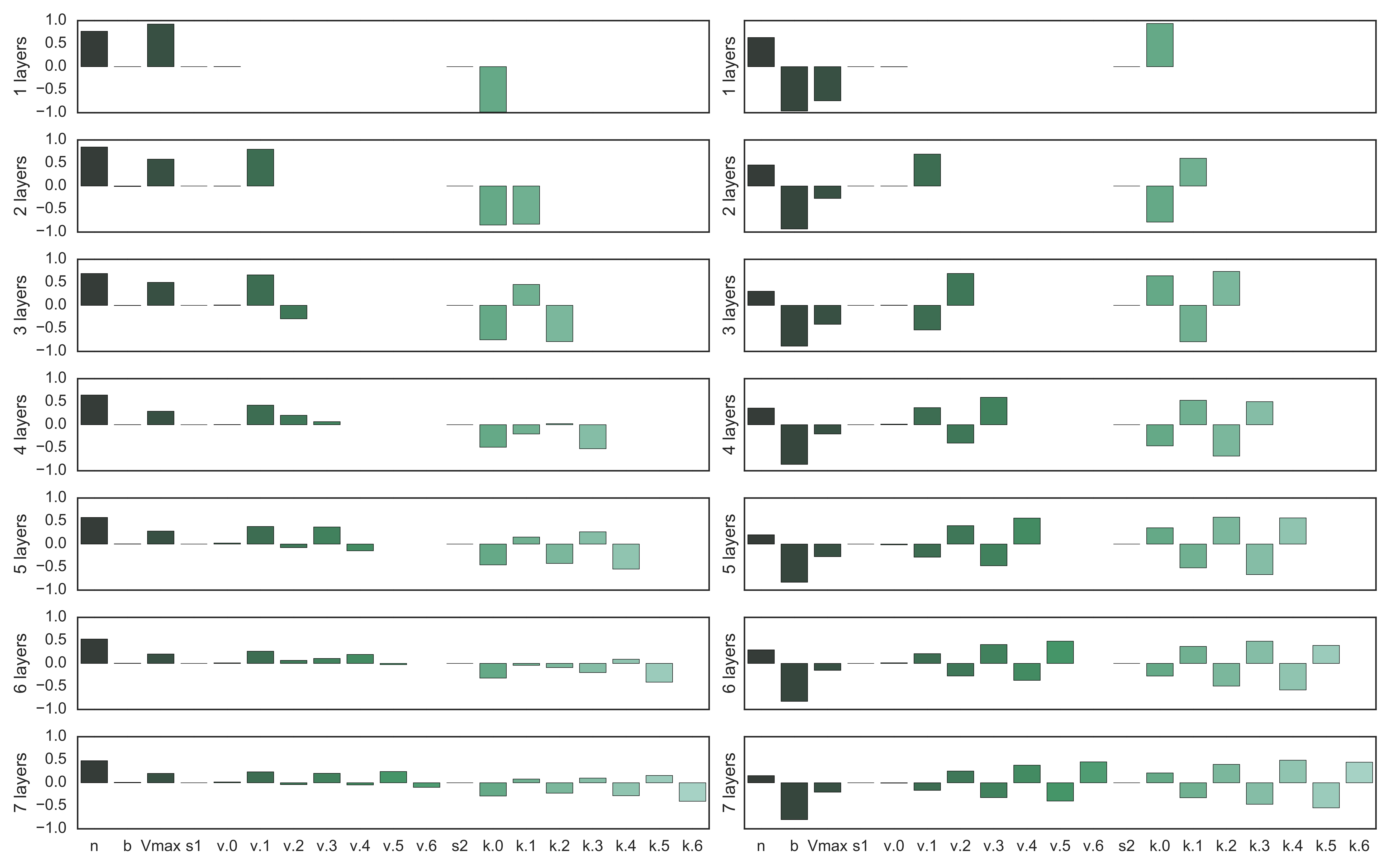
1 layers
2 layers
3 layers
4 layers
5 layers
6 layers
7 layers
1.0
0.5
0.0
−0.5
−1.0
n
b
Vmax
s1
v.0
v.1
v.2
v.3
v.4
v.5
v.6
s2
k.0
k.1
k.2
k.3
k.4
k.5
k.6
