# Supplementary material for: Digital logic circuits in yeast with CRISPR-dCas9 NOR gates
Source: Nat Commun. 2017 May 25;8:15459. doi: 10.1038/ncomms15459 (PMC5458518; doi:10.1038/ncomms15459)
Supplement: Supplementary Software 1 — R cytometry data processing scripts and mathematical modeling scripts [file ncomms15459-s3.zip › Supplementary Software 1/PRCC_Results/PRCC_combined_20161208.csv_Fig.pdf]

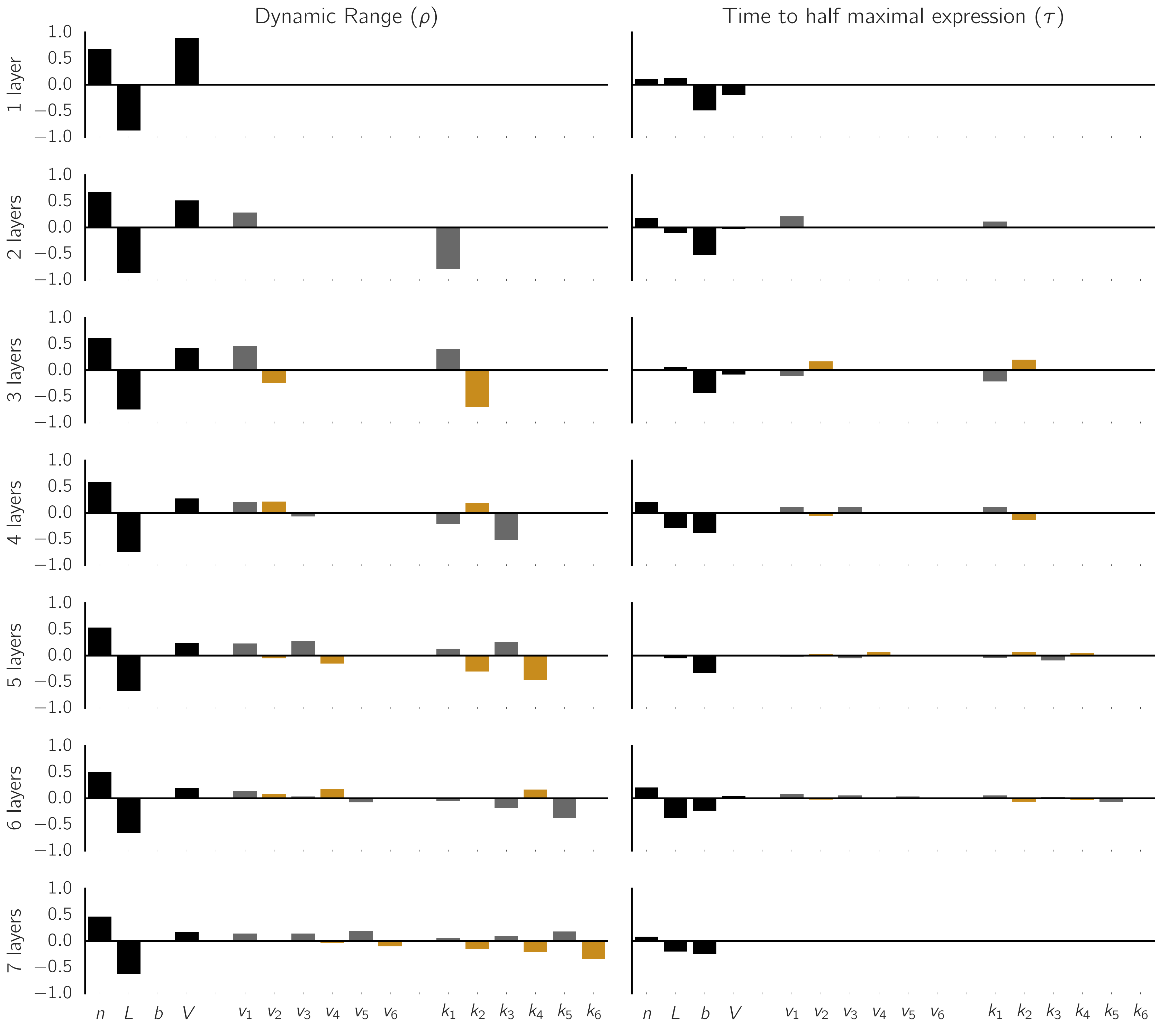

Dynamic Range (ρ)
Time to half maximal expression (τ)
1 layer
2 layers
3 layers
4 layers
5 layers
6 layers
7 layers
1.0
0.5
0.0
−0.5
−1.0
n
L
b
V
v1
v2
v3
v4
v5
v6
k1
k2
k3
k4
k5
k6
