# Supplementary material for: Digital logic circuits in yeast with CRISPR-dCas9 NOR gates
Source: Nat Commun. 2017 May 25;8:15459. doi: 10.1038/ncomms15459 (PMC5458518; doi:10.1038/ncomms15459)
Supplement: Peer Review File [file ncomms15459-s4.pdf]

Editorial Note: this manuscript has been previously reviewed at another journal that is not operating a transparent peer review scheme. This document only contains reviewer comments and rebuttal letters for versions considered at Nature Communications.
